# A systematic review of bone graft products used in lumbar interbody fusion procedures for degenerative disc disease

**DOI:** 10.1016/j.xnsj.2024.100579

**Published:** 2025-01-11

**Authors:** Anita Fitzgerald, Rachael McCool, Emma Carr, Paul Miller, Katie Reddish, Cynthia C Lohr, Elena Annoni, Brandon Lawrence

**Affiliations:** a York Health Economics Consortium, Enterprise House, University of York, York, United Kingdom; b Medtronic, Office of Medical Affairs, Memphis, TN, United States; c Health Economics, Policy & Reimbursement, Medtronic International Trading Sàrl, 1131 Tolochenaz, Switzerland; d Department of Orthopaedics, University of Utah, UT, United States

**Keywords:** Bone graft, Degenerative disc disease, Iliac crest bone graft, Infuse™, Lumbar interbody fusion, Spinal fusion, Bone morphogenetic protein, BMP-2, rhBMP-2

## Abstract

**Background:**

Degenerative disc disease (DDD) is associated with chronic lower back pain that may have impacts on individual's quality of life and functional ability. Lumbar interbody fusion can be carried out with a variety of bone grafting products, the choice depends on several factors including the patient, site, procedure, cost and indication. This systematic review (SR) intends to validate and consolidate the existing evidence base supporting bone graft materials related to lumbar interbody fusion procedures for DDD, specifically anterior lumbar interbody fusion (ALIF) and oblique lumbar interbody fusion (OLIF).

**Methods:**

An SR was conducted in February 2023. Clinical and economic studies of adults with DDD in regions L2 to S1 undergoing lumbar interbody fusion with Infuse™, allograft, synthetic bone grafts, demineralized bone matrices or cell-based matrices were eligible for inclusion.

**Results:**

Twenty-one studies (reported in 25 publications) were included in the review. Eighteen studies (reported in 22 publications) reported clinical outcomes, while 4 studies reported economic outcomes. Nine studies (in 5 publications) investigated Infuse™, including 3 randomized controlled trials (RCTs), one cohort study and 4 case series. Ten studies investigated allograft bone, bone harvested from the vertebral spur combined with apacerum powder, or tricalcium phosphate soaked in autologous bone marrow aspirate, including one RCT, 2 cohort studies, and 7 case series.

**Conclusions:**

The SR shows that Infuse™ offers comparable results to iliac crest bone graft with the benefit of not requiring harvested bone and offers significant benefits in surgical time and blood loss. There is a lack of comparative evidence for any other bone grafts identified in this SR, highlighting the need for further well-designed studies to be conducted in this area.

## Introduction and background

Degenerative disc disease (DDD) in the lumbar spine is associated with chronic lower back pain that can radiate to the lower limbs, joint stiffness, paraesthesia and weakness [1]. The prevalence of lumbar DDD ranges from 33% to 90% depending on age and geography [[2], [3], [4]]. In Europe, the incidence of symptomatic lumbar DDD is estimated to be 8,586 new cases per 100,000 annually, which may notably increase when asymptomatic cases are considered [5]. In the US and Canada, incidence is estimated to be 6,818 new cases per 100,000 annually [5], and lower back pain in general is thought to cost over US$100 million annually, including direct and indirect costs and loss of productivity [6].

Patients with DDD are often managed conservatively at first. The quality of life and functional ability of patients with therapy-resistant (conservatively treated) severe DDD is greatly impaired due to the intensity of pain and limited mobility [[7], [8], [9]]. Several studies have reported evidence recommending lumbar interbody fusion for people with DDD after 6 months to one year of failed conservative management [[10], [11], [12], [13], [14]]. Guidelines and policy recommendations from several professional societies affirm the use of lumbar interbody fusion with various bone graft materials and autografts for patients with DDD. Such endorsements come from esteemed organizations including the International Society for the Advancement of Spine Surgery, the North American Spine Society, and NHS England [[15], [16], [17], [18]].

There are different approaches for lumbar interbody fusion procedures, including anterior lumbar interbody fusion (ALIF) and oblique lateral interbody fusion (OLIF). In the Anterior Lumbar Interbody Fusion (ALIF) procedure, surgeons gain access from the front, inserting an implant filled with bone grafts into the disc space between 2 vertebrae. The Oblique Lumbar Interbody Fusion (OLIF), a minimally invasive technique, also involves a similar placement of an implant, filled with bone graft material. Here, the lumbar spine is accessed from both the front and side of the body. Despite their different surgical approaches, both procedures essentially use an implant placed within the disc space [10].

The choice of bone graft used during the fusion procedure depends on several factors, including: product indications, the likelihood of a successful fusion, patient morbidity relating to potential autograft harvest, patient biological status, comorbidities and habits, and the cost of any bone graft materials [19]. Autologous iliac crest bone graft (ICBG) is considered the gold standard for lumbar interbody fusion but there are associated limitations [20]. Alternative graft materials, such as bone graft extenders and recombinant human bone morphogenetic protein-2 (rhBMP-2) were developed as alternative graft materials [21].

Infuse™ Bone Graft consists of rhBMP-2 or dibotermin alpha applied to an absorbable collagen sponge (ACS) carrier scaffold. rhBMP-2 on ACS is a highly osteoinductive replacement for autograft bone that can be used for certain lumbar interbody fusion procedures in conjunction with certain interbody devices and has been approved by the Food and Drug Administration (FDA) since 2002 [22].

Given that Infuse™ has been in the market for over 20 years, it is important to validate existing knowledge by reviewing the published evidence base for all bone grafts in this indication. Therefore, a systematic review was conducted to identify and consolidate the evidence base supporting bone graft materials in adult lumbar spinal fusion procedures for DDD (in regions L2 to S1), specifically ALIF and OLIF.

## Material and methods

This SR was undertaken according to the principles of systematic reviewing embodied in the Cochrane handbook [23] and guidance published by the Centre for Reviews and Dissemination [24]. The review was reported in line with the Preferred Reporting Items for Systematic Reviews and Meta-Analyses (PRISMA) guidelines [25].

### Eligibility criteria

Studies of adults with DDD in regions L2 to S1 undergoing ALIF or OLIF (treating a single level) with Infuse™ (on-label use only), synthetic bone grafts, demineralized bone matrices or cell-based matrices alone or compared with any other intervention (including ICBG) were eligible for inclusion. Eligible outcomes included clinical outcomes, patient-reported outcomes, utilities, direct elicitation methods, direct and indirect monetary cost data, indirect nonmonetary cost and resource use data, and economic evaluation outcomes. The full eligibility criteria are detailed in Appendix A.

### Searches

A MEDLINE (OvidSP) search strategy was designed by an Information Specialist to identify studies of eligible interventions in DDD patients undergoing ALIF or OLIF. Full search strategies and methods are presented in Appendix B.

The strategy comprised 2 concepts which were combined as follows: (DDD, ALIF or OLIF) AND interventions. The strategy excluded animal studies, other ineligible publication types which were unlikely to yield relevant study reports (editorials and news items) and records with the phrase ‘case report’ in the title. Reflecting the eligibility criteria, the strategy was restricted to studies published in English language and from 2000 to date, to cover studies published since the launch of Infuse™.

The literature searches were conducted in February 2023 in a range of databases, trial registers and HTA webpages. Full details can be found in Appendix C.

### Study selection, data extraction, risk of bias, and synthesis

Two independent researchers screened the search results at (both title and abstract and at full text review) against the eligibility criteria in the protocol. Any disagreements were discussed with a third reviewer. Studies excluded following full-text review are listed in Appendix D.

One researcher extracted data and carried out the risk of bias assessment for each of the eligible studies and a second researcher checked all data points. A data extraction sheet was developed in Excel and piloted on a number of studies before progressing to full data extraction. The following elements were extracted from all eligible studies: bibliographic details, study characteristics, patient baseline characteristics, details of intervention, details of statistical analyses and prespecified outcomes (including unit of measurement and effect size). Randomized controlled trials were assessed using the Cochrane risk of bias tool for RCTs (version 2.0) [23], while the cohort studies and case series were assessed using the Joanna Briggs Institute checklists [26,27]. Economic evaluations were assessed using the Checklist specified in NICE STA guidance, adapted from Drummond (1996) [28]. A risk of bias assessment was not conducted for studies reporting utilities, patient-reported outcome measures or costs. The full list of data extraction elements can be found in Appendix E and detailed risk of bias assessments are reported in Appendix F.

Included studies were summarized through a narrative synthesis alongside tables that provide data on the methods and results. Options for indirect comparisons were explored, but no connected networks were identified.

## Results

**Figure 1:**
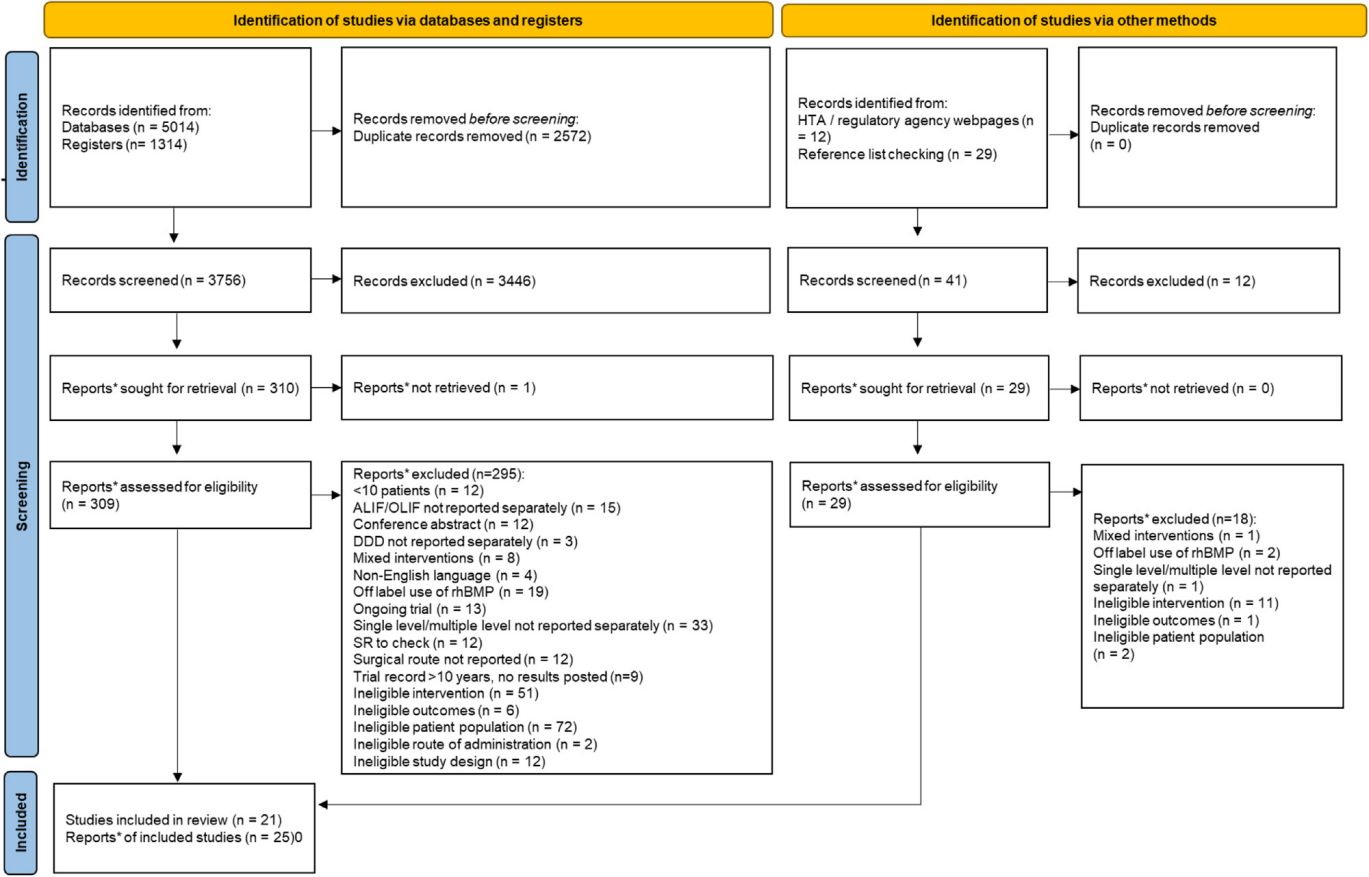
PRISMA flow diagram. *"Note that a “report” could be a journal article, preprint, conference abstract, study register entry, clinical study report, dissertation, unpublished manuscript, government report or any other document providing relevant information": https://www.bmj.com/content/372/bmj.n71. *Adapted from:* Page MJ, McKenzie JE, Bossuyt PM, Boutron I, Hoffmann TC, Mulrow CD, et al. The PRISMA 2020 statement: an updated guideline for reporting systematic reviews. BMJ 2021;372:n71. doi: 10.1136/bmj.n71. For more information, visit: http://www.prisma-statement.org/

Of the 3,797 records assessed for relevance, 3,458 records were excluded following the first pass and title and abstract review. The full texts for 339 records were sought for retrieval; one record was unobtainable, and 338 records were retrieved and assessed for relevance. twenty-one studies (reported in 25 documents) were included in the review reporting data for 3321 patients. Eighteen studies (reported in 22 documents) reported clinical outcomes, while 4 studies reported economic outcomes. A PRISMA flow diagram is reported in Fig. 1.

Nine studies investigated Infuse™, including 3 RCTs [[29], [30], [31], [32]] one cohort study [33], and 4 case series (in 5 publications) [21,[34], [35], [36], [37], [38]]. Two RCTs compared Infuse™ to iliac crest bone graft (ICBG) [[29], [30], [31]], and one compared Infuse™ to artificial disc replacement [32]. The cohort study compared different types of supplemental fixation in ALIF using Infuse™ [33]. Ten studies investigated other bone graft interventions, including one RCT [39] which compared ICBG used via different surgical approaches, 2 cohort studies of allograft bone (in which only one treatment arm of patients having ALIF were eligible and are, therefore, considered as case series for the purpose of this review) [40,41] and 7 case series (of which 5 assessed allograft bone [[42], [43], [44], [45], [46]], one assessed bone harvested from the vertebral spur combined with apacerum powder [47] and one assessed tricalcium phosphate soaked in autologous bone marrow aspirate [48]. Patient characteristics were reported inconsistently across the identified studies with several key baseline characteristics either not reported or inadequately reported, making it difficult to draw conclusions about the comparability of the study populations. All RCTs were assessed as having a high risk of bias, detailed data are reported in Appendix F.

RCT evidence has been prioritized in the following sections as this is the most robust evidence identified. This includes 2 RCTs comparing Infuse™ with ICBG, one comparing Infuse™ with artificial disc replacement and one comparing mini-open and laparoscopic approaches for ICBG. No RCT evidence was identified for any other bone graft. Observational evidence is discussed briefly for each outcome and results tables are available in the supplementary appendix.

### Fusion outcomes

Four RCTs reported data on fusion outcomes. Two RCTs comparing Infuse™ with ICBG reported comparable fusion rates at various time points. The first RCT with a large patient population (n=252) found comparable fusion rates between Infuse™ and ICBG at 6, 12, or 24 months [30]. The second RCT, albeit with a small sample size (n=14), yielded similar results [29].

Another RCT, comparing Infuse™ with artificial disc replacement, reported a nonunion rate of 4.1% (7/172) among Infuse™ patients [32]. A fourth RCT, comparing laparoscopic ALIF to mini-open ALIF with autologous bone grafts found no significant difference in fusion rates between groups, with a nonunion rate of 9.1% observed in both groups [39].

Fusion outcomes for RCTs are reported in Table 1.

**Table 1:** Fusion outcomes reported in randomized controlled trials

Study	Intervention	Timepoint of assessment	Fusion success n (%)	p-value
Boden, 2000 [29]	Infuse™	3 months	10/11 (90.9)	p=.3956
	ICBG		2/3 (66.7)	
	Infuse™	6 months	11/11 (100)	p=.2143
	ICBG		2/3 (66.7)	
	Infuse™	12 months	11/11 (100)	p=.2143
	ICBG		2/3 (66.7)	
	Infuse™	24 months	11/11 (100)	p=.2143
	ICBG		2/3 (66.7)	
Burkus, 2002 [30]	Infuse™	6 months	128/132 (97)	p=.903
	ICBG		115/120 (95.8)	
	Infuse™	12 months	127/131 (96.9)	p=.116
	ICBG		112/121 (92.6)	
	Infuse™	24 months	120/127 (94.5)	p=.102
	ICBG		102/115 (88.7)	
Chung, 2003 [39]	ABG, Lap ALIF	Mean: 43 months (R 36-49)	20/22 (91)	NR
	ABG, MO ALIF	Mean: 30 months (R 24-40)	20 /22 (91)	

Abbreviations: ABG: autologous bone graft; ALIF: anterior lumbar interbody fusion; ICBG: iliac crest bone graft; MO: mini-open; NR: not reported.; R: Range.

In addition to the RCTs, one cohort study and 11 case series reported fusion outcomes. A cohort study of Infuse™ reported failed fusion as evidence of pseudoarthrosis; no patient was reported to have failed fusion [33]. while 4 case series reported fusion rates between 94.1 and 100% when using Infuse™ [35,36,37, 38]. One study using autologous bone grafts and apacerum powder reported fusion rates of 90.1% [47] and 6 separate studies assessing allografts reported rates between 86.7% and 100% [[40], [41], [42], [43],^45^, ^46^]. Detailed data for the case series can be found in Appendix G.

Insufficient data regarding nonunion rates were reported to allow definitive conclusions to be drawn. Please refer to Table 1 for a summary of fusion outcomes in RCTs.

### Secondary surgical outcomes

In the RCTs identified, there was inconsistent reporting of the number of patients undergoing secondary surgeries (implant removals, supplemental fixation, revisions, reoperations). Two RCTs provided data on secondary surgery outcomes, with no significant differences reported between Infuse™ and ICBG for most outcomes, including implant removals, supplemental fixation, revisions, and reoperations [30,38]. A third RCT reported significantly higher reoperation rates among patient receiving artificial disc replacement compared to Infuse™ (5.4% vs. 1.7%, p=.046) but no significant differences for other outcomes including implant removal, supplemental fixation and revision surgery [32].

One RCT, comparing laparoscopic ALIF to mini-open ALIF with autologous bone grafts, found no difference in the number of conversions to open surgery between groups [39].

In the observational studies identified, one case series supported the findings of Infuse™ RCTs reporting supplementary surgery rates of between 0.7% (1/134) and 5.2% (7/134) [38]. Four case series investigating allograft bone [41,46,49] and autograft bone with apacerum powder [47] reported low rates of reoperations or revisions (0% to 3.8%). Detailed data for the case series can be found in Appendix G.

### Procedural outcomes

Data from 2 RCTs reported significantly shorter operative time among Infuse™ patients compared to ICBG [30] and artificial disc replacement [32], however the mean difference in both studies was 24 minutes and was not considered to be clinically significant [30,32]. Another RCT compared laparoscopic ALIF to mini-open ALIF among patients receiving autologous grafts. Mean operative time was significantly shorter in the mini-open ALIF group compared to the laparoscopic group (83 minutes vs 158 minutes, p=.001) [39].

Data from 2 RCTs reported significantly less blood loss among Infuse™ patients compared with ICBG [29,30] and one RCT reported significantly less blood loss for Infuse™ compared with artificial disc replacement [32], however the mean difference in these studies was less than 150 ml and it is not considered to be clinically significant. One RCT compared laparoscopic ALIF to mini-open ALIF among patients receiving autologous grafts and found no significant differences in mean blood loss between groups [39].

The largest RCT reported no significant differences in length of stay in the Infuse™ group compared with the ICBG group (3.1 days vs 3.3 days, p=.254) [30]; a second RCT with very small patient numbers (n=14) reported similar results [29,31]. A third RCT reported no difference in length of stay between Infuse™ and artificial disc replacement (2.3 days vs 2.2 days) [32]. One RCT compared laparoscopic ALIF to mini-open ALIF among patients receiving autologous grafts and reported no significant differences in mean length of stay between groups [39].

RCT data are reported in Table 2.

**Table 2:** Procedural outcomes reported in randomized controlled trials

Study ID	Intervention	n	Operative time Mean hours/minutes (SD)	Blood loss Mean ml (SD)	Length of stay Mean days (SD)			
Boden 2000 [29]	Infuse™	11	1.9 hrs (0.2)	p=.006	95 (31)	p=.400	2.0 (0.6)	p=.18
	ICBG	3	3.3 hrs (0.6)		167 (117)		3.3 (1.4)	
Burkus 2002 [30]	Infuse™	143	1.6 hrs (0.6)	p<.001	109.8 (117.3)	p= .017	3.1 (1.6)	p=.254
	ICBG	136	2.0 hrs (0.7)		153.1 (179.1)		3.3 (1.3)	
Gornet 2011 [32]	Infuse™	172	1.4 hrs (0.6)	p<.001	95.2 (106.9)	p<.001	2.3 (1.1)	p=.581
	ADR	405	1.8 hrs (0.6)		240.7 (301.1)		2.2 (1.3)	
Chung 2003 [39]	ABG Lap ALIF	27	158 mins (R 90-330)	p=.001	85 (R10-300)	NS	3.9 (R 2-7)	NS
	ABG, MO ALIF	24	83 mins (R 40-150)		68 (R 50-150)		3.4 (R 2-6)	

Abbreviations: ABG: autologous bone graft; ADR: artificial disc replacement; ALIF: anterior lumbar interbody fusion; hrs: Hours; ICBG: Iliac crest bone graft; Lap –Laparoscopic, ml –Millilitres; MO: Mini-open; NS: not significant; R: Range; SD: Standard deviation.

In the observational studies identified, 3 case series reported varying operative times for Infuse™ patients. In one study, a mean operative time of 1.9 hours was reported [38], another study reported a mean operative time of 102 minutes [36] while the third reported median operative times of between 164 and 250 minutes across 3 different types of fixation devices used with Infuse™ [33].

Four observational studies reported varying volumes of blood loss for Infuse™ patients; one cohort study reported median blood loss of between 75 and 150ml across 3 different types of fixation devices used with Infuse™ [33]. One case series reported a mean blood loss of 33, however no units were reported so it is unclear whether this measurement was in millilitres [36]. A second case series reported a mean blood loss of 146.1ml (SD 406.2) [38], while the third reported a mean blood loss of 115ml (SD 151) [37].

Three observational studies reported varying length of stay (LoS) durations for Infuse™ patients; one study reported a mean LoS of 1.2 days (SD 1.1) [38], a second study reported a mean LoS of 1.0 days [36] while the other reported a median LoS of between 3 and 4 days across 3 different types of fixation devices used with Infuse™ [33].

A further 5 case series evaluating other bone graft interventions reported varied operating times, blood loss and length of stay data [[40], [41], [42],^47^,^48^].

In patients who received tricalcium phosphate mean operative time was 69.2 minutes, mean blood loss was 29.4ml and LoS was 2.6 days [48]. In patients who received autogenous vertebral spur and apacerum powder mean operative time 155.5 minutes, mean blood loss was 63.7ml and mean LoS was 6.9 days [47]. Across the 3 studies of patients who received allograft bone mean operative time ranged from 76.5 to 159.35 minutes, blood loss ranged from 90.3 to 461.54 and mean LoS ranged from 4 to 6.44 days [[40], [41], [42]].

Detailed case series data is reported in Appendix G

### Patient reported outcomes

Data from 2 RCTs indicate that Oswestry disability index (ODI) scores were comparable between Infuse™ and ICBG in [[29], [30], [31]] while data from one RCT showed significantly greater improvements in ODI among artificial disc replacement patients compared with Infuse™ patients at all time points from baseline to 2 years [32]. One RCT comparing laparoscopic ALIF to mini-open ALIF among patients receiving autologous bone grafts reported no significant differences in mean change in ODI or back pain between groups [39]. RCT data for ODI scores are reported in Table 3.

**Table 3:** ODI scores reported in randomized controlled trials

Study	Timepoint of assessment	Intervention	n	Mean ODI score (SD)	p-value	Mean change from baseline (SD)	p-value	Improvement in ODI n (%)	p-value
Boden 2000 [29]	Baseline	Infuse™	11	38.9 (3.5)	NR	NR	NR	0 (0)^1^	NS
		ICBG	3	34.7 (7.7)	NR	NR	NR	0 (0)	
	3 months	Infuse™	11	29.8 (6.3)	NR	NR	NR	6 (54.7)	p=.091
		ICBG	3	42.7 (8.4)	NR	NR	NR	0 (0)	
	6 months	Infuse™	11	26.9 (6)	NR	NR	NR	7 (63.6)	p=.923
		ICBG	3	28 (15)	NR	NR	NR	2 (66.7)	
	12 months	Infuse™	11	17.7 (5.1)	NR	NR	NR	10 (90.9)	p=.288
		ICBG	3	27.3 (15.6)	NR	NR	NR	2 (66.7)	
	24 months	Infuse™	11	13.5 (5.1)	NR	-25 (NR)	NR	10 (90.9)	p=.288
		ICBG	3	20 (12.9)	NR	-15 (NR)	NR	2 (66.7)	
Burkus 2002 [30]	Baseline	Infuse™	143	53.7 (12.7)	NR	NR	NR	NR	NA
		ICBG	136	55.1 (11.8)	NR	NR	NR	NR	NA
	6 weeks	Infuse™	140	42.1 (NR)	NR	-11.4 (NR)	NR	NR	NA
		ICBG	131	41.4 (NR)	NR	-13.6 (NR)	NR	NR	NA
	3 months	Infuse™	141	33.5 (17.6)	NR	-19.9 (NR)	NR	NR	NA
		ICBG	134	34.2 (18.5)	NR	-20.8 (NR)	NR	NR	NA
	6 months	Infuse™	136	29.3 (18.8)	NR	-24.4 (NR)	NR	NR	NA
		ICBG	131	29.4 (18.2)	NR	-25.4 (NR)	NR	NR	NA
	12 months	Infuse™	130	25.5 (18.2)	NR	-27.7 (NR)	NR	NR	NA
		ICBG	125	25.6 (19.1)	NR	-28.9 (NR)	NR	NR	NA
	24 months	Infuse™	122	23.9 (18.8)	NR	-29.0 (NR)	NR	NR	NA
		ICBG	108	23.8 (20.7)	NR	-29.5 (NR)	NR	NR	NA
Burkus 2009 [21]	4 years	Infuse™	48	20.6 (19.2)	NR	NR	NR	NR	NR
	6 years	Infuse™	70	25.8 (19.3)	NR	NR	NR	NR	NR
Gornet 2011 [32]	Baseline	Infuse™	172	54.5 (12.6)	p=.288	NR (NR)	NR	NR	NA
		ADR	405	53.3 (13)		NR (NR)	NR	NR	NA
	6 weeks	Infuse™	169	41.4 (17.1)	p<.001	13.1 (16.8)	p<.001	72 (42.8)^2^	p<.001
		ADR	401	31.2 (19.5)		22.1 (19.7)		258 (64.3)	
	3 months	Infuse™	166	32 (16.8)	p<.001	22.4 (18.4)	p<.001	107 (64.2)	p=.004
		ADR	393	23.4 (18.8)		29.8 (19.4)		298 (75.9)	
	6 months	Infuse™	163	26.8 (17.3)	p<.001	27.4 (17.5)	p<.001	120 (73.4)	p=.014
		ADR	391	20.1 (18.3)		33.2 (19.2)		322 (82.3)	
	12 months	Infuse™	163	25.3 (19.8)	p<.001	29 (21.1)	p<.001	117 (71.8)	p=.004
		ADR	393	19.2 (18.2)		33.9 (19.8)		324 (82.5)	
	24 months	Infuse™	145	24.8 (19.6)	p=.004	29.2 (19.4)	p=.004	108 (74.6)	p=.041
		ADR	379	19.4 (20.2)		33.8 (21.1)		312 (82.2)	
Chung 2003 [39]	Baseline	ABG Lap ALIF	22	41 (NR)	NR	NR	NR	NA	NR
		ABG MO ALIF	22	43 (NR)	NR	NR	NR	NA	
	Mean: 43 months (Range 36-49)	ABG Lap ALIF	22	25 (NR)	NR	38 (NR)	NS	NA	NR
	Mean: 30 months (Range 24-40)	ABG MO ALIF	22	23 (NR)	NR	47 (NR)		NA	NR

Abbreviations: ABG: autologous bone graft; ADR: artificial disc replacement; ALIFL: anterior lumbar interbody fusion; ICBG: iliac crest bone graft; Lap: laparoscopy; MO: mini-open; n: number of participants; NA: not applicable; NR: not reported; NS: not significant; ODI: Oswestry Disability Index; SD: standard deviation.

Notes:

1. Clinical success was defined as an improvement of at least a 15% over the preoperative score.

2. Improvement of at least 15 points on the Oswestry score.

Increased ODI scores denote a reduction in disability.

In terms of other measurements, data from one RCT reported no significant differences in either back or leg pain between Infuse™ and ICBG patients at any time point [30,31]. This study also found comparable SF-36 scores. A second RCT reported significantly a greater reduction in back pain among artificial disc replacement patients compared with Infuse™ patients at 6 weeks, 3 months, 6 months, 12 months and 24 months, but no significant difference in leg pain [32]. The same study also found significantly greater improvements in the SF-36 physical component scores among artificial disc replacement patients compared to Infuse™ patients. Early differences in SF-36 mental component improvements reported in the artificial disc replacement group compared to the Infuse™ group were not sustained at later time points [32].

One further RCT comparing laparoscopic ALIF to mini-open ALIF among patients receiving autologous grafts reported similar mean change in back pain between groups [39].

No studies reported data for the PROMIS measure.

In the observational studies, 4 case series reporting Infuse™ data supported the RCT data in that Infuse™ may reduce ODI rates from baseline [34,[36], [37], [38]]. Three case series reported a range of ODI back and leg pain scores across different interventions [41,42,44,46,47]. Data from the RCTs are reported in Table 3; detailed case series data can be found in Appendix G.

### Complications

Two RCTs reported data in peri-operative complications, with mixed results; one trial reported significantly more peri-operative complications among Infuse™ patients compared with artificial disc replacement [32], while the other showed no differences between Infuse™ and ICBG [30]. One RCT compared laparoscopic ALIF to mini-open ALIF among patients receiving autologous grafts reported no significant differences in the number of peri-operative complications between groups were reported [39].

Three RCTs reported data on long-term complications (further details can be found in Table 4). Data from 2 RCTs reported no significant differences between Infuse™ patients and ICBG [29] or artificial disc replacement [29,32]. One RCT reported donor site complications in ICBG patients at 24 months [30] among 8 patients (5.9%) including injuries to the lateral femoral cutaneous nerve, avulsion fractures of the anterior superior iliac crest, infection and haematoma [30].

**Table 4:** Complications reported in RCTs

Study ID	Timepoint	Intervention	Complication description	Number (%) of peri-operative and long-term complications	p-value for difference between study arms
Boden 2000 [29]	24 months	Infuse™	Total clinical adverse events	3/11 (27.3)	p=.207
		ICBG		2/3 (66.7)	
Burkus 2002 [30]	24 months	Infuse™	Retrograde ejaculation	6/146 (4.1)	NR
		Infuse™	Intra-operative vascular event^1^	6/143 (4.2)	p=.50
		ICBG		5/136 (3.7)	
Gornet 2011 [32]	24 months	Infuse™	At least one adverse event	153/172 (89.0)	p=.289
		ADR		345/405 (85.2)	
		Infuse™	Any serious adverse event^2^	71/172 (41.3)	p=.714
		ADR		174/405 (43.0)	
		Infuse™	Possibly device-related adverse event^3^	22/172 (12.8)	p<.001
		ADR		17/405 (4.2)	
		Infuse™	Serious possibly device related adverse events	12/172 (7.0)	p<.001
		ADR		4/405 (1.0)	
Chung 2003 [39]	Peri-operative	ABG Lap ALIF	Any peri-operative complication	3/27 (11.1)	p=.7392
		ABG, MO ALIF		2/24 (8.3)	

Abbreviations: ABG: autologous bone graft; ADR: artificial disc replacement; ALIF: anterior lumbar interbody fusion; ICBG: Iliac crest bone graft; Lap, laparoscopic; MO, mini-open; NR: not reported; RCT: randomized controlled trial.

Notes

1. Eleven intraoperative vascular events occurred: 6 were in the rhBMP-2 group (4.2%) and 5 were in the autograft group (3.7%). The most common injury (6 of 11) was a laceration of the iliac vein. Two control group patients developed deep venous thrombosis and were treated with anticoagulation medications. No patients in the investigational group developed a deep venous thrombosis. This may, in part, be secondary to the reduced surgical times.

2. WHO Grade 3 or 4 adverse events were considered as serious.

3. These were deemed to be associated with the device, or the surgical procedure.

In the observational studies, 1 cohort study [33] and 2 case series [[34], [35], [36]] were identified reporting varying numbers of complications. Two case series reported long-term complications associated with Infuse ™ [35,37]. One study reported that 12% of patients had a postoperative complication [35], while the other reported that 5.8% had a major complication [37].

Four case series assessing allograft bone [40,42,44, 46] and one assessing autogenous vertebral spur and apacerum powder [47] reported long term complications or general adverse events and reported varied numbers of long-term complications ranging from 0% [46] to 15.4% [40].There were insufficient data reported about medication usage to draw conclusions about the effectiveness of Infuse™ or other bone graft products. RCT data are reported in Table 4; detailed case series data are reported in Appendix G.

### Economic outcomes

One study published in 2009 reported the overall savings per case for Infuse™ compared with autograft in ALIF surgery in France, Germany and the UK, ranging from €8,483 to €9,191 [50]. Cost savings offset the upfront price for Infuse™ driven mainly by reduced productivity loss due to an earlier return-to-work for patients (43 days faster than ICBG).

One cost minimization analysis, published in 2007, in a US setting compared the direct costs of lumbar total disc replacement with CHARITE Artificial Disc versus 3 different techniques for lumbar fusion in DDD including ALIF with Infuse™. The total cost per patient for ALIF with Infuse™ and LT-Cages was $22,668 from the hospital perspective; and $28,892 to $32,196 from the payer perspective. These made ALIF with Infuse™ the costliest option compared to total disc replacement with CHARITE Artificial Disc ($16,601; and $17,614 to $24,885 respectively); ALIF with ICBG ($18,596; and $23,778 to $32,960); and IPLIF with ICBG ($22,662; and $25,052 to $31,620).

One prospective case series in Australia reported the direct cost of ALIF with Infuse™ (the study was conducted from 2009 to 2012, no cost year was reported) was approximately AU$10,500 per level and comprised a PEEK cage, anterior plate, 3 or 4 bone screws, the plate cover and a small rhBMP-2 kit [37]. While the authors concluded that ALIF was cost-effective, no cost-effectiveness analysis was presented.

Polly 2003 reported the total direct medical cost of Infuse™ in the US: $3,380. This was $9 cheaper than spinal fusion care for ICBG. The study claimed operation time savings for Infuse™ compared with ICBG, as well as 0.5 fewer days in hospital.

No economic studies were identified for grafts other than infuse. No studies reported utilities data associated with Infuse™ or other grafts.

## Discussion

This SR identified 21 studies reporting clinical or economic outcomes in adults with DDD in regions L2 to S1, undergoing ALIF or OLIF with eligible bone graft products. Nine studies investigated Infuse™, including 3 RCTs comparing with ICBG [[29], [30], [31]] or artificial disc replacement [32], one cohort study comparing different types of supplemental fixation in ALIF with Infuse™ [33] and a 4 case series [21,[34], [35], [36], [37], [38]]. Ten studies investigated other bone graft interventions including one RCT [39] comparing ICBG used via different surgical approaches, and 9 case series (of which 7 assessed allograft bone [[40], [41], [42], [43], [44], [45], [46]], one assessed bone harvested from the vertebral spur combined with apacerum powder [47] and one assessed tricalcium phosphate soaked in autologous bone marrow aspirate [48]).

The RCT data identified in this SR indicate that Infuse™ bone graft is associated with fusion rates ranging from 90.9 to 100% [29,30]. When compared with the gold standard, ICBG, Infuse™ demonstrated comparable in fusion rates, suggesting that it is an effective treatment option for patients with DDD. Findings indicate that Infuse™ was also associated with shorter operating time and less blood loss, although these were not clinically significant. Secondary surgeries (including implant removals, supplemental fixation, revisions and re-operations) reported inconclusive results. Perioperative complications and long-term complications also showed inconclusive or conflicting results. Infuse™ demonstrated an improvement in patient-reported measures from baseline, a finding supported by 2 RCTs and single-arm evidence. Safety data indicated a higher occurrence of peri-operative-related complications in patients undergoing ALIF with Infuse™ compared with those undergoing artificial disc replacement.

Burkus 2003 [38] reported an integrated analysis of 679 patients combining data from the Burkus 2002 RCT and a further 2 single arm trials of Infuse™ and ICBG. While the integrated analysis was not eligible for this review, both the RCT [30] and the single arm trial of Infuse™ [38] have been included. The results of the integrated analysis generally support the findings of this review; Infuse™ was associated with a statistically significant reduction in operative time and blood loss and comparable rates of secondary surgeries. The integrated analysis also indicated a statistically significant fusion rate for Infuse™ compare with ICBG at 2 years (94.4% vs 89.4%, p=.022) and concluded overall superiority of Infuse™ [38].

A recent classification system developed the AOSpine Knowledge Forum reported a 3-tier system for the levels of evidence for osteobiologics based on the type of data available (human, animal or in vitro) and the quality of the available evidence. Application of this system showed that Infuse™ is currently the only bone graft product identified that is supported with A1 grade evidence [51]. Single arm and case series evidence, which is the level of evidence identified for the other graft products, is considered to be A4 grade evidence.

This review has several strengths; it was informed by extensive searches conducted in a range of databases and searches of the reference lists of relevant reviews and studies to ensure that as many relevant studies as possible were identified. No search limits were placed on date, or study design. However, the review's findings should be interpreted in light of several limitations. The scope and depth of the SR were constrained by the limited number and methodological shortcomings of existing studies, especially for those bone grafts that were only supported by single arm evidence. Despite exhaustive searches across multiple databases and reference lists, the review only revealed A1 evidence supporting the use of Infuse^TM^ in adults with DDD in regions L2 to S1 undergoing lumbar interbody fusion. An overall lack of high-quality, comparative trials means that the superiority of any single bone graft product over another cannot be concluded. Variability in study design, patient characteristics, interventions, and outcomes further complicated the interpretation and application of the findings.

## Conclusion

The SR shows that Infuse™ offers comparable results to iliac crest bone graft with the benefit of not requiring harvested bone and offers significant benefits in surgical time and blood loss. Infuse is currently the only commercially available bone graft product indicated for the treatment of patients with DDD undergoing ALIF or OLIF in regions L2 to S1 supported by RCTs. There is a lack of comparative evidence for other bone grafts identified in this SR, highlighting the need for further well-designed studies to be conducted in this area. Such research should focus on trials with larger sample sizes, adequately powered to detect differences in primary outcomes, to reinforce the evidence base and enable more definitive clinical decision making.

## Declaration of competing interest

One or more authors declare potential competing financial interests or personal relationships as specified on required ICMJE-NASSJ Disclosure Forms.

## Appendix Supplementary materials

Image, application 1
