# Supplementary material for: A systematic review of bone graft products used in lumbar interbody fusion procedures for degenerative disc disease
Source: N Am Spine Soc J. 2025 Jan 11;21:100579. doi: 10.1016/j.xnsj.2024.100579 (PMC11800106; doi:10.1016/j.xnsj.2024.100579)
Supplement: Supplementary file 1 [file mmc1.docx]

SUPPLEMENTARY MATERIAL

# Appendix A: Eligibility Criteria

**Table A1: Eligibility criteria**

| **Population** | Adults with lumbar DDD in spinal regions L2 to S1 managed with ALIF or OLIF surgery (treating a single level). Studies assessing single and multi-level treatment were eligible if more than 80% of patients are receiving treatment for a single level, or subgroup data were available for this population. |
| --- | --- |
| **Interventions** | Studies investigating patients undergoing ALIF or OLIF for DDD performed using:   - Infuse™ Bone Graft used as a stand-alone graft material on an absorbable collagen sponge - Synthetic bone grafts - Demineralized bone matrices - Cell-based matrices |
| **Comparators** | Studies comparing the above interventions to any or no comparator were eligible. |
| **Outcomes** | - Clinical outcomes: fusion success rate, non-union rate, time to complete fusion, proportion of patients with fusion at specific time points, need for harvest surgery, surgery for failed fusion/revision surgery, donor site complications, peri-operative/inpatient complications, long-term complications, medication usage. - Patient-reported outcome measures: ODI, SRS, VAS back pain, VAS leg pain, NRS pain, SF-36, SF-12, PROMIS Measures. - Utilities: EQ-5D, SF-6D, 15D, HUI2, HUI. - Direct elicitation methods - Direct costs data (monetary): surgery, staff time, downstream costs. - Indirect costs data (monetary or non-monetary): number of workdays missed. - Resource use data (non-monetary): number of days in hospital, staff time. - Economic evaluation outcomes: effectiveness outcomes (e.g. QALYs), total costs, incremental analyses outcomes (e.g. ICERs). |

**Abbreviations**: ALIF – anterior lumbar interbody fusion, DDD – degenerative disc disease, EQ5D – EuroQol 5 dimensions, HUI – Health Utilities Index, ICER – incremental cost-effectiveness ratio, NRS – numerical rating scale, ODI – Oswestry Disability Index, OLIF – oblique lumbar interbody fusion, PROMIS – Patient-Reported Outcomes Measurement Information System, QALY – quality adjusted life year, rhBMP-2 – recombinant human bone morphogenetic protein-2, SF – short form, SRS – Scoliosis Research Society, VAS – visual analogue scale.

# Appendix B: Search Strategy

The MEDLINE strategy was devised using a combination of subject indexing terms and free text search terms in the Title, Abstract, Keyword Heading Word, Registry Number, Name of Substance Word and Original Title fields.

The population terms in the strategy were designed to retrieve database records that explicitly refer to DDD / ALIF / OLIF and the lumbar context.

The final Ovid MEDLINE strategy was peer-reviewed before execution by a second Information Specialist. Peer review considered the appropriateness of the strategy for the review scope and eligibility criteria, inclusion of key search terms, errors in spelling, syntax and line combinations, and application of exclusions.

We conducted searches using each database or resource listed, translating the agreed Ovid MEDLINE strategy appropriately. Translation included consideration of differences in database interfaces and functionality, in addition to variation in indexing languages and thesauri. The final translated database strategies were peer-reviewed by a second Information Specialist. Peer review considered the appropriateness of the translation for the database being searched, errors in syntax and line combinations, and application of exclusions.

Where possible, we downloaded the results of searches in a tagged format and loaded them into bibliographic management software (EndNote). The results were deduplicated using several algorithms and the deduplicated references held in a duplicates EndNote database for checking if required. Results from resources which did not allow export in a format compatible with EndNote were saved in Word or Excel documents as appropriate and manually deduplicated.

1. **Source:** **MEDLINE (MEDALL)**

Interface / URL: OvidSP

Database coverage dates: 1946 to 23 February 2023

Search date: 24 February 2023

Retrieved records: 1883

Search strategy:

1 intervertebral disc degeneration/ 7343

2 exp spondylosis/ 8575

3 radiculopathy/ 5767

4 spinal cord compression/ 11805

5 spinal cord diseases/ 13727

6 osteophyte/ 1059

7 intervertebral disc displacement/ 20094

8 spinal stenosis/ 7122

9 ((disc* or disk* or intervertebra* or inter-vertebra*) adj5 (degenerat* or degrad*)).ti,ab,kf. 17533

10 (spondylos* or spondylolys* or spondylolisthes* or discopath* or diskopath* or spondylochondros* or spondylo-chondros* or spondylodiscitis or spondylo-discitis or spondylodiskitis or spondylo-diskitis or spondylo-listhes* or spondylo-lysthes* or olisthes*).ti,ab,kf. 14096

11 ((disc* or disk* or intervertebra* or inter-vertebra*) adj5 (disease* or hernia* or protrud* or protrus* or prolaps* or slip* or displace* or chondrosis)).ti,ab,kf. 86313

12 (radiculopath* or radiculitis or polyradiculopath* or polyradiculitis or myelopath* or neuromyelopath* or osteophy* or spondylot* or retrolisthes*).ti,ab,kf. 30696

13 ((spine*1 or spinal or cord*1) adj5 (compress* or disease* or disorder*)).ti,ab,kf. 36458

14 ((spine*1 or spinal) adj5 stenos*).ti,ab,kf. 8329

15 spinal fusion/ 30890

16 arthrodesis/ 10117

17 (spondylodes* or arthrodes* or syndes* or ((spinal or spine*1 or vertebra*) adj5 fus*)).ti,ab,kf. 32720

18 ((anterior or oblique or psoas or prepsoas or interbody) adj5 (fus* or infus*)).ti,ab,kf. 15563

19 or/1-18 233137

20 lumbar vertebrae/ 60114

21 lumbosacral region/ 14038

22 (lumbar or lumbalis).ti,ab,kf. 125068

23 lumbo*.ti,ab,kf. 19438

24 or/20-23 158010

25 19 and 24 52117

26 (OLIF51 or OLIF25 or OLIF or OLLIF* or ALIF).ti,ab,kf. 1168

27 (lumbar adj3 fus*).ti,ab,kf. 9513

28 or/25-27 52499

29 bone morphogenetic proteins/ 13080

30 bone morphogenetic protein 2/ 8483

31 (dibotermin alfa or dibotermin alpha or dibotermine alfa or dibotermine alpha).ti,ab,kf,rn,nm,ot. 9

32 ((bone morphogen* or osteogen* or bone morphologic or bone morphological) adj3 (protein* or factor* or polypeptide* or poly-peptide*)).ti,ab,kf. 25270

33 (bmp or bmp2 or rhbmp or rhbmp2).ti,ab,kf,rn,nm,ot. 26733

34 bone transplantation/ 32859

35 bone matrix/ 4260

36 bone regeneration/ 18078

37 bone substitutes/ 10290

38 ilium/tr 2275

39 ((iliac or ilium or ilial) adj5 (transplant* or graft* or autograft* or allograft* or autotransplant* or homotransplant* or heterotransplant*)).ti,ab,kf,ot. 6893

40 (bone* adj5 (transplant* or graft* or autograft* or allograft* or autotransplant* or homotransplant* or heterotransplant* or cage* or scaffold* or demineraliz* or demineralis* or regenerat* or matrix or matrices or decalcif* or reimplant* or substitut* or replac*)).ti,ab,kf,ot. 129662

41 (ICBG or ICBGs).ti,ab,kf,ot. 220

42 (osteotransplant* or osteo-transplant*).ti,ab,kf,ot. 2

43 (osteograft* or osteo-graft*).ti,ab,kf,ot. 3

44 (osteoconduct* or osteo-conduct*).ti,ab,kf,ot. 4392

45 (osteoinduct* or osteo-induct*).ti,ab,kf,ot. 5297

46 (inductos* or induct os or induct ostm or induct osr or infuse*2 or truscient* or nebotermin* or 246539-15-1 or T472P45MG6).ti,ab,kf,rn,nm,ot. 56266

47 (i-factor* or mastergraft* or vitoss* or actifuse* or bonus* or chronos* or fibergraft* or magnetos* or grafton* or magnifuse* or DBF* or DBX* or osteosurge* or puros* or stagraft* or intergrow* or allocraft* or vesuvius* or trinity* or osteocel* or vivigen* or bonus triad*).ti,ab,kf,ot. 11855

48 or/29-47 253541

49 28 and 48 3466

50 exp animals/ not humans/ 5095326

51 (news or editorial or case reports).pt. or case report.ti. 3217861

52 or/50-51 8249260

53 49 not 52 2597

54 limit 53 to english language 2213

55 limit 54 to yr="2000 -Current" 1883

1. **Source: Embase**

Interface / URL: OvidSP

Database coverage dates: 1974 to 23 February 2023

Search date: 24 February 2023

Retrieved records: 2630

Search strategy:

1 intervertebral disk degeneration/ 15055

2 spondylosis/ 5464

3 spondylolysis/ 2136

4 spondylolisthesis/ 10518

5 radiculopathy/ 11653

6 spinal cord compression/ 18067

7 spinal cord disease/ 17421

8 osteophyte/ 10000

9 intervertebral disk hernia/ 17984

10 lumbar disk hernia/ 8910

11 exp vertebral canal stenosis/ 15900

12 radiculitis/ 1079

13 retrolisthesis/ 110

14 ((disc* or disk* or intervertebra* or inter-vertebra*) adj5 (degenerat* or degrad*)).ti,ab,kf,dq. 23608

15 (spondylos* or spondylolys* or spondylolisthes* or discopath* or diskopath* or spondylochondros* or spondylo-chondros* or spondylodiscitis or spondylo-discitis or spondylodiskitis or spondylo-diskitis or spondylo-listhes* or spondylo-lysthes* or olisthes*).ti,ab,kf,dq. 18798

16 ((disc* or disk* or intervertebra* or inter-vertebra*) adj5 (disease* or hernia* or protrud* or protrus* or prolaps* or slip* or displace* or chondrosis)).ti,ab,kf,dq. 119202

17 (radiculopath* or radiculitis or polyradiculopath* or polyradiculitis or myelopath* or neuromyelopath* or osteophy* or spondylot* or retrolisthes*).ti,ab,kf,dq. 42874

18 ((spine*1 or spinal or cord*1) adj5 (compress* or disease* or disorder*)).ti,ab,kf,dq. 47405

19 ((spine*1 or spinal) adj5 stenos*).ti,ab,kf,dq. 11868

20 spine fusion/ 29917

21 arthrodesis/ 14140

22 spondylodesis/ 1563

23 anterior spine fusion/ 3863

24 (spondylodes* or arthrodes* or syndes* or ((spinal or spine*1 or vertebra*) adj5 fus*)).ti,ab,kf,dq. 42195

25 ((anterior or oblique or psoas or prepsoas or interbody) adj5 (fus* or infus*)).ti,ab,kf,dq. 20739

26 or/1-25 311243

27 exp lumbar vertebra/ 24235

28 lumbar spine/ 57443

29 lumbar disk/ 3930

30 lumbar region/ 5863

31 lumbar spinal cord/ 5130

32 lumbosacral region/ 1877

33 lumbosacral spine/ 5102

34 (lumbar or lumbalis).ti,ab,kf,dq. 177907

35 lumbo*.ti,ab,kf,dq. 24171

36 or/27-35 212207

37 26 and 36 64518

38 (OLIF51 or OLIF25 or OLIF or OLLIF* or ALIF).ti,ab,kf,dq. 1657

39 (lumbar* adj3 fus*).ti,ab,kf,dq. 13143

40 or/37-39 65265

41 bone morphogenetic protein/ 17695

42 bone morphogenetic protein 2/ 17243

43 recombinant bone morphogenetic protein/ 174

44 recombinant bone morphogenetic protein 2/ 3154

45 (dibotermin alfa or dibotermin alpha or dibotermine alfa or dibotermine alpha).ti,ab,kf,dq,rn,tn,dy,ot. 17

46 ((bone morphogen* or osteogen* or bone morphologic or bone morphological) adj3 (protein* or factor* or polypeptide* or poly-peptide*)).ti,ab,kf,dq. 32050

47 (bmp or bmp2 or rhbmp or rhbmp2).ti,ab,kf,dq,rn,tn,dy,ot. 35680

48 bone transplantation/ 20236

49 exp bone graft/ 40952

50 bone matrix/ 9486

51 bone regeneration/ 32026

52 bone prosthesis/ 10709

53 ((iliac or ilium or ilial) adj5 (transplant* or graft* or autograft* or allograft* or autotransplant* or homotransplant* or heterotransplant*)).ti,ab,kf,dq,dv,my,ot. 8779

54 (bone* adj5 (transplant* or graft* or autograft* or allograft* or autotransplant* or homotransplant* or heterotransplant* or cage* or scaffold* or demineraliz* or demineralis* or regenerat* or matrix or matrices or decalcif* or reimplant* or substitut* or replac*)).ti,ab,kf,dq,dv,my,ot. 162757

55 (ICBG or ICBGs).ti,ab,kf,dq,dv,my,ot. 324

56 (osteotransplant* or osteo-transplant*).ti,ab,kf,dq,dv,my,ot. 5

57 (osteograft* or osteo-graft*).ti,ab,kf,dq,dv,my,ot. 3

58 (osteoconduct* or osteo-conduct*).ti,ab,kf,dq,dv,my,ot. 5036

59 (osteoinduct* or osteo-induct*).ti,ab,kf,dq,dv,my,ot. 6384

60 (inductos* or induct os or induct ostm or induct osr or infuse*2 or truscient* or nebotermin* or 246539-15-1 or T472P45MG6).ti,ab,kf,dq,rn,tn,dy,ot. 80473

61 (i-factor* or mastergraft* or vitoss* or actifuse* or bonus* or chronos* or fibergraft* or magnetos* or grafton* or magnifuse* or DBF* or DBX* or osteosurge* or puros* or stagraft* or intergrow* or allocraft* or vesuvius* or trinity* or osteocel* or vivigen* or bonus triad*).ti,ab,kf,dq,dv,my,ot. 13917

62 or/41-61 345323

63 40 and 62 4847

64 (animal/ or animal experiment/ or animal model/ or animal tissue/ or nonhuman/) not exp human/ 6743996

65 editorial.pt. or case report.ti. 1137314

66 conference abstract.pt. 4687091

67 preprint.pt. 57184

68 or/64-67 12275858

69 63 not 68 3440

70 limit 69 to english language 2941

71 limit 70 to yr="2000 -Current" 2630

1. **Source:** **Cochrane Database of Systematic Reviews (CDSR)**

Interface / URL: Cochrane Library / Wiley

Database coverage dates: Information not found. Issue searched: Issue 2 of 12, February 2023

Search date: 24 February 2023

Retrieved records: 2

Search strategy:

#1 [mh ^"intervertebral disc degeneration"] 521

#2 [mh "spondylosis"] 507

#3 [mh ^"radiculopathy"] 599

#4 [mh ^"spinal cord compression"] 133

#5 [mh ^"spinal cord diseases"] 193

#6 [mh ^"osteophyte"] 32

#7 [mh ^"intervertebral disc displacement"] 1150

#8 [mh ^"spinal stenosis"] 551

#9 ((disc* or disk* or intervertebra* or inter-vertebra*) near/5 (degenerat* or degrad*)):ti,ab,kw 1465

#10 (spondylos* or spondylolys* or spondylolisthes* or discopath* or diskopath* or spondylochondros* or spondylo-chondros* or spondylodiscitis or spondylo-discitis or spondylodiskitis or spondylo-diskitis or spondylo-listhes* or spondylo-lysthes* or olisthes*):ti,ab,kw 2076

#11 ((disc* or disk* or intervertebra* or inter-vertebra*) near/5 (disease* or hernia* or protrud* or protrus* or prolaps* or slip* or displace* or chondrosis)):ti,ab,kw 7491

#12 (radiculopath* or radiculitis or polyradiculopath* or polyradiculitis or myelopath* or neuromyelopath* or osteophy* or spondylot* or retrolisthes*):ti,ab,kw 2833

#13 ((spine* or spinal or cord*) near/5 (compress* or disease* or disorder*)):ti,ab,kw 3870

#14 ((spine* or spinal) near/5 stenos*):ti,ab,kw 1460

#15 [mh ^"spinal fusion"] 1326

#16 [mh ^"arthrodesis"] 115

#17 (spondylodes* or arthrodes* or syndes* or ((spinal or spine* or vertebra*) near/5 fus*)):ti,ab,kw 3269

#18 ((anterior or oblique or psoas or prepsoas or interbody) near/5 (fus* or infus*)):ti,ab,kw 1669

#19 #1 OR #2 OR #3 OR #4 OR #5 OR #6 OR #7 OR #8 OR #9 OR #10 OR #11 OR #12 OR #13 OR #14 OR #15 OR #16 OR #17 OR #18 17282

#20 [mh ^"lumbar vertebrae"] 3290

#21 [mh ^"lumbosacral region"] 605

#22 (lumbar or lumbalis):ti,ab,kw 18901

#23 lumbo*:ti,ab,kw 3062

#24 #20 OR #21 OR #22 OR #23 20673

#25 #19 AND #24 6297

#26 (OLIF51 or OLIF25 or OLIF or OLLIF* or ALIF):ti,ab,kw 78

#27 (lumbar near/3 fus*):ti,ab,kw 1491

#28 #25 OR #26 OR #27 6408

#29 [mh ^"bone morphogenetic proteins"] 136

#30 [mh ^"bone morphogenetic protein 2"] 92

#31 ("dibotermin alfa" or "dibotermin alpha" or "dibotermine alfa" or "dibotermine alpha"):ti,ab,kw 10

#32 ((bone next morphogen* or osteogen* or "bone morphologic" or "bone morphological") near/3 (protein* or factor* or polypeptide* or poly-peptide*)):ti,ab,kw 481

#33 (bmp or bmp2 or rhbmp or rhbmp2):ti,ab,kw 518

#34 [mh ^"bone transplantation"] 1102

#35 [mh ^"bone matrix"] 165

#36 [mh ^"bone regeneration"] 603

#37 [mh ^"bone substitutes"] 640

#38 [mh ^"ilium"/TR] 136

#39 ((iliac or ilium or ilial) near/5 (transplant* or graft* or autograft* or allograft* or autotransplant* or homotransplant* or heterotransplant*)):ti,ab,kw 571

#40 (bone* near/5 (transplant* or graft* or autograft* or allograft* or autotransplant* or homotransplant* or heterotransplant* or cage* or scaffold* or demineraliz* or demineralis* or regenerat* or matrix or matrices or decalcif* or reimplant* or substitut* or replac*)):ti,ab,kw 9970

#41 (ICBG or ICBGs):ti,ab,kw 49

#42 (osteotransplant* or osteo-transplant*):ti,ab,kw 0

#43 (osteograft* or osteo-graft*):ti,ab,kw 0

#44 (osteoconduct* or osteo-conduct*):ti,ab,kw 145

#45 (osteoinduct* or osteo-induct*):ti,ab,kw 132

#46 (inductos* or "induct os" or "induct ostm" or "induct osr" or infuse* or truscient* or nebotermin* or "246539-15-1" or T472P45MG6):ti,ab,kw 8402

#47 (i-factor* or mastergraft* or vitoss* or actifuse* or bonus* or chronos* or fibergraft* or magnetos* or grafton* or magnifuse* or DBF* or DBX* or osteosurge* or puros* or stagraft* or intergrow* or allocraft* or vesuvius* or trinity* or osteocel* or vivigen* or bonus next triad*):ti,ab,kw 729

#48 #29 or #30 or #31 or #32 or #33 or #34 or #35 or #36 or #37 or #38 or #39 or #40 or #41 or #42 or #43 or #44 or #45 or #46 or #47 19432

#49 #28 and #48 with Cochrane Library publication date Between Jan 2000 and Dec 2023, in Cochrane Reviews, Cochrane Protocols 2

1. **Source:** **Cochrane Central Register of Controlled Trials (CENTRAL)**

Interface / URL: Cochrane Library / Wiley

Database coverage dates: Information not found. Issue searched: Issue 2 of 12, February 2023

Search date: 24 February 2023

Retrieved records: 473

Search strategy:

#1 [mh ^"intervertebral disc degeneration"] 521

#2 [mh "spondylosis"] 507

#3 [mh ^"radiculopathy"] 599

#4 [mh ^"spinal cord compression"] 133

#5 [mh ^"spinal cord diseases"] 193

#6 [mh ^"osteophyte"] 32

#7 [mh ^"intervertebral disc displacement"] 1150

#8 [mh ^"spinal stenosis"] 551

#9 ((disc* or disk* or intervertebra* or inter-vertebra*) near/5 (degenerat* or degrad*)) 1535

#10 (spondylos* or spondylolys* or spondylolisthes* or discopath* or diskopath* or spondylochondros* or spondylo-chondros* or spondylodiscitis or spondylo-discitis or spondylodiskitis or spondylo-diskitis or spondylo-listhes* or spondylo-lysthes* or olisthes*) 2187

#11 ((disc* or disk* or intervertebra* or inter-vertebra*) near/5 (disease* or hernia* or protrud* or protrus* or prolaps* or slip* or displace* or chondrosis)) 8110

#12 (radiculopath* or radiculitis or polyradiculopath* or polyradiculitis or myelopath* or neuromyelopath* or osteophy* or spondylot* or retrolisthes*) 2989

#13 ((spine* or spinal or cord*) near/5 (compress* or disease* or disorder*)) 4777

#14 ((spine* or spinal) near/5 stenos*) 1520

#15 [mh ^"spinal fusion"] 1326

#16 [mh ^"arthrodesis"] 115

#17 (spondylodes* or arthrodes* or syndes* or ((spinal or spine* or vertebra*) near/5 fus*)) 3373

#18 ((anterior or oblique or psoas or prepsoas or interbody) near/5 (fus* or infus*)) 1713

#19 #1 OR #2 OR #3 OR #4 OR #5 OR #6 OR #7 OR #8 OR #9 OR #10 OR #11 OR #12 OR #13 OR #14 OR #15 OR #16 OR #17 OR #18 18789

#20 [mh ^"lumbar vertebrae"] 3290

#21 [mh ^"lumbosacral region"] 605

#22 (lumbar or lumbalis) 19394

#23 lumbo* 3195

#24 #20 OR #21 OR #22 OR #23 21217

#25 #19 AND #24 6603

#26 (OLIF51 or OLIF25 or OLIF or OLLIF* or ALIF) 94

#27 (lumbar near/3 fus*) 1535

#28 #25 OR #26 OR #27 6721

#29 [mh ^"bone morphogenetic proteins"] 136

#30 [mh ^"bone morphogenetic protein 2"] 92

#31 ("dibotermin alfa" or "dibotermin alpha" or "dibotermine alfa" or "dibotermine alpha") 10

#32 ((bone next morphogen* or osteogen* or "bone morphologic" or "bone morphological") near/3 (protein* or factor* or polypeptide* or poly-peptide*)) 541

#33 (bmp or bmp2 or rhbmp or rhbmp2) 562

#34 [mh ^"bone transplantation"] 1102

#35 [mh ^"bone matrix"] 165

#36 [mh ^"bone regeneration"] 603

#37 [mh ^"bone substitutes"] 640

#38 [mh ^"ilium"/TR] 136

#39 ((iliac or ilium or ilial) near/5 (transplant* or graft* or autograft* or allograft* or autotransplant* or homotransplant* or heterotransplant*)) 607

#40 (bone* near/5 (transplant* or graft* or autograft* or allograft* or autotransplant* or homotransplant* or heterotransplant* or cage* or scaffold* or demineraliz* or demineralis* or regenerat* or matrix or matrices or decalcif* or reimplant* or substitut* or replac*)) 12077

#41 (ICBG or ICBGs) 49

#42 (osteotransplant* or osteo-transplant*) 0

#43 (osteograft* or osteo-graft*) 0

#44 (osteoconduct* or osteo-conduct*) 155

#45 (osteoinduct* or osteo-induct*) 138

#46 (inductos* or "induct os" or "induct ostm" or "induct osr" or infuse* or truscient* or nebotermin* or "246539-15-1" or T472P45MG6) 8679

#47 (i-factor* or mastergraft* or vitoss* or actifuse* or bonus* or chronos* or fibergraft* or magnetos* or grafton* or magnifuse* or DBF* or DBX* or osteosurge* or puros* or stagraft* or intergrow* or allocraft* or vesuvius* or trinity* or osteocel* or vivigen* or bonus next triad*) 1406

#48 #29 or #30 or #31 or #32 or #33 or #34 or #35 or #36 or #37 or #38 or #39 or #40 or #41 or #42 or #43 or #44 or #45 or #46 or #47 22484

#49 #28 and #48 with Publication Year from 2000 to 2023, in Trials 473

1. **Source: HTA database**

Interface / URL: https://database.inahta.org/

Database coverage dates: Information not found. The former database was produced by the CRD until March 2018, at which time the addition of records was stopped as INAHTA was in the process of rebuilding the new database platform. In July 2019, the database records were exported from the CRD platform and imported into the new platform that was developed by INAHTA. The rebuild of the new platform was launched in June 2020.

Search date: 24 February 2023

Retrieved records: 12

Search strategy:

Search lines were entered into the search box as written below. Line combinations were carried out via the Search history. The following limits were applied using the filters on the results page:

Limited by publication year: 2000 to 2023

Limited by language: English

The above limits reduced the number of records retrieved from 13 to 12.

1 "intervertebral disc degeneration"[mh] 25

2 "spondylosis"[mhe] 3

3 "radiculopathy"[mh] 4

4 "spinal cord compression"[mh] 6

5 "spinal cord diseases"[mh] 0

6 "osteophyte"[mh] 0

7 "intervertebral disc displacement"[mh] 13

8 "spinal stenosis"[mh] 22

9 (disc* OR disk* OR inter*) AND (degenerat* OR degrad*) 127

10 spondylo* OR discopath* OR diskopath* OR olisthes* 32

11 (disc* OR disk* OR inter*) AND (disease* OR hernia* OR protrud* OR protrus* OR prolaps* OR slip* OR displace* OR chondrosis) 1296

12 radiculopath* OR radiculitis OR polyradiculopath* OR polyradiculitis OR myelopath* OR neuromyelopath* OR osteophy* OR retrolisthes* 17

13 (spine* OR spinal OR cord*) AND (compress* OR disease* OR disorder*) 162

14 (spine* OR spinal) AND stenos* 27

15 "spinal fusion"[mh] 48

16 arthrodes* OR syndes* OR ((spinal OR spine* OR vertebra*) AND fus*) 72

17 "arthrodesis"[mh] 6

18 (anterior OR oblique OR psoas OR prepsoas OR interbody) AND (fus* OR infus*) 22

19 #18 OR #17 OR #16 OR #15 OR #14 OR #13 OR #12 OR #11 OR #10 OR #9 OR #8 OR #7 OR #6 OR #5 OR #4 OR #3 OR #2 OR #1 1492

20 "lumbar vertebrae"[mh] 93

21 "lumbosacral region"[mh] 15

22 lumbar OR lumbalis 131

23 lumbo* 11

24 #23 OR #22 OR #21 OR #20 167

25 #24 AND #19 107

26 OLIF51 OR OLIF25 OR OLIF OR OLLIF* OR ALIF 2

27 lumbar AND fus* 40

28 #27 OR #26 OR #25 109

29 "bone morphogenetic proteins"[mh] 13

30 "bone morphogenetic protein 2"[mh] 2

31 "dibotermin alfa" OR "dibotermin alpha" OR "dibotermine alfa" OR "dibotermine alpha" 0

32 ((bone AND morphogen*) OR osteogen* OR "bone morphologic" OR "bone morphological") AND (protein* OR factor* OR poly*) 21

33 bmp OR bmp2 OR rhbmp OR rhbmp2 9

34 "bone transplantation"[mh] 17

35 "bone matrix"[mh] 1

36 "bone regeneration"[mh] 7

37 "bone substitutes"[mh] 4

38 "ilium"[mh] 0

39 (iliac OR ilium OR ilial) AND (transplant* OR graft* OR autograft* OR allograft* OR autotransplant* OR homotransplant* OR heterotransplant*) 7

40 bone* AND (transplant* OR graft* OR autograft* OR allograft* OR autotransplant* OR homotransplant* OR heterotransplant* OR cage* OR scaffold* OR demineraliz* OR demineralis* OR regenerat* OR matrix OR matrices OR decalcif* OR reimplant* OR substitut* OR replac*) 165

41 ICBG OR ICBGs 0

42 osteotransplant* OR (osteo AND transplant*) 2

43 osteograft* OR (osteo AND graft*) 1

44 osteoconduct* OR (osteo AND conduct*) 0

45 osteoinduct* OR (osteo AND induct*) 2

46 inductos* OR "induct os" OR "induct ostm" OR "induct osr" OR infuse* OR truscient* OR nebotermin* OR 246539-15-1 OR T472P45MG6 14

47 factor* OR mastergraft* OR vitoss* OR actifuse* OR bonus* OR chronos* OR fibergraft* OR magnetos* OR grafton* OR magnifuse* OR DBF* OR DBX* OR osteosurge* OR puros* OR stagraft* OR intergrow* OR allocraft* OR vesuvius* OR trinity* OR osteocel* OR vivigen* OR "bonus triad" OR "bonus triadr" OR "bonus triadtm" 1400

48 #47 OR #46 OR #45 OR #44 OR #43 OR #42 OR #41 OR #40 OR #39 OR #38 OR #37 OR #36 OR #35 OR #34 OR #33 OR #32 OR #31 OR #30 OR #29 1581

49 #48 AND #28 13

1. **Source: NHS Economic Evaluation Database (NHS EED)**

Interface / URL: https://www.crd.york.ac.uk/CRDWeb

Database coverage dates: Information not found. Bibliographic records were published on NHS EED until 31st March 2015. Searches of MEDLINE, Embase, CINAHL, PsycINFO and PubMed were continued until the end of the 2014.

Search date: 24 February 2023

Retrieved records: 14

Search strategy:

1 MeSH DESCRIPTOR intervertebral disc degeneration 68

2 MeSH DESCRIPTOR spondylosis EXPLODE ALL TREES 67

3 MeSH DESCRIPTOR radiculopathy 41

4 MeSH DESCRIPTOR spinal cord compression 31

5 MeSH DESCRIPTOR spinal cord diseases 17

6 MeSH DESCRIPTOR osteophyte 1

7 MeSH DESCRIPTOR intervertebral disc displacement 95

8 MeSH DESCRIPTOR spinal stenosis 64

9 (((disc* OR disk* OR intervertebra* OR inter-vertebra*) AND (degenerat* OR degrad*))) 295

10 ((spondylos* OR spondylolys* OR spondylolisthes* OR discopath* OR diskopath* OR spondylochondros* OR spondylo-chondros* OR spondylodiscitis OR spondylo-discitis OR spondylodiskitis OR spondylo-diskitis OR spondylo-listhes* OR spondylo-lysthes* OR olisthes*)) 117

11 (((disc* OR disk* OR intervertebra* OR inter-vertebra*) AND (disease* OR hernia* OR protrud* OR protrus* OR prolaps* OR slip* OR displace* OR chondrosis))) 9775

12 ((radiculopath* OR radiculitis OR polyradiculopath* OR polyradiculitis OR myelopath* OR neuromyelopath* OR osteophy* OR spondylot* OR retrolisthes*)) 151

13 (((spine* OR spinal OR cord*) AND (compress* OR disease* OR disorder*))) 980

14 (((spine* OR spinal) AND stenos*)) 105

15 MeSH DESCRIPTOR spinal fusion 301

16 MeSH DESCRIPTOR arthrodesis 31

17 ((spondylodes* OR arthrodes* OR syndes* OR ((spinal OR spine* OR vertebra*) AND fus*))) 420

18 (((anterior OR oblique OR psoas OR prepsoas OR interbody) AND (fus* OR infus*))) 139

19 #1 OR #2 OR #3 OR #4 OR #5 OR #6 OR #7 OR #8 OR #9 OR #10 OR #11 OR #12 OR #13 OR #14 OR #15 OR #16 OR #17 OR #18 10746

20 MeSH DESCRIPTOR lumbar vertebrae 383

21 MeSH DESCRIPTOR lumbosacral region 41

22 ((lumbar OR lumbalis)) 765

23 (lumbo*) 108

24 #20 OR #21 OR #22 OR #23 827

25 #19 AND #24 516

26 ((OLIF51 OR OLIF25 OR OLIF OR OLLIF* OR ALIF)) 3

27 ((lumbar AND fus*)) 200

28 #25 OR #26 OR #27 517

29 MeSH DESCRIPTOR bone morphogenetic proteins 31

30 MeSH DESCRIPTOR bone morphogenetic protein 2 19

31 ((dibotermin alfa OR dibotermin alpha OR dibotermine alfa OR dibotermine alpha)) 0

32 (((bone morphogen* OR osteogen* OR bone morphologic OR bone morphological) adj3 (protein* OR factor* OR polypeptide* OR poly-peptide*))) 55

33 ((bmp OR bmp2 OR rhbmp OR rhbmp2)) 26

34 MeSH DESCRIPTOR bone transplantation 124

35 MeSH DESCRIPTOR bone matrix 1

36 MeSH DESCRIPTOR bone regeneration 34

37 MeSH DESCRIPTOR bone substitutes 45

38 MeSH DESCRIPTOR ilium WITH QUALIFIER TR 10

39 (((iliac OR ilium OR ilial) AND (transplant* OR graft* OR autograft* OR allograft* OR autotransplant* OR homotransplant* OR heterotransplant*))) 59

40 (((bone* AND (transplant* OR graft* OR autograft* OR allograft* OR autotransplant* OR homotransplant* OR heterotransplant* OR cage* OR scaffold* OR demineraliz* OR demineralis* OR regenerat* OR matrix OR matrices OR decalcif* OR reimplant* OR substitut* OR replac*)))) 996

41 (ICBG OR ICBGs) 0

42 (((osteotransplant* OR osteo-transplant*))) 0

43 (((osteograft* OR osteo-graft*))) 0

44 (((osteoconduct* OR osteo-conduct*))) 2

45 (((osteoinduct* OR osteo-induct*))) 4

46 (((inductos* OR induct os OR induct ostm OR induct osr OR infuse* OR truscient* OR nebotermin* OR 246539-15-1 OR T472P45MG6))) 85

47 (((i-factor* OR mastergraft* OR vitoss* OR actifuse* OR bonus* OR chronos* OR fibergraft* OR magnetos* OR grafton* OR magnifuse* OR DBF* OR DBX* OR osteosurge* OR puros* OR stagraft* OR intergrow* OR allocraft* OR vesuvius* OR trinity* OR osteocel* OR vivigen* OR bonus triad*))) 8

48 #29 OR #30 OR #31 OR #32 OR #33 OR #34 OR #35 OR #36 OR #37 OR #38 OR #39 OR #40 OR #41 OR #42 OR #43 OR #44 OR #45 OR #46 OR #47 1115

49 #28 AND #48 67

50 (#49) IN NHSEED FROM 2000 TO 2023 14

1. **Source: Econlit**

Interface / URL: OvidSP

Database coverage dates: 1886 to 16 February 2023

Search date: 24 February 2023

Retrieved records: 0

Search strategy:

1 ((disc* or disk* or intervertebra* or inter-vertebra*) adj5 (degenerat* or degrad*)).af. 80

2 (spondylos* or spondylolys* or spondylolisthes* or discopath* or diskopath* or spondylochondros* or spondylo-chondros* or spondylodiscitis or spondylo-discitis or spondylodiskitis or spondylo-diskitis or spondylo-listhes* or spondylo-lysthes* or olisthes*).af. 0

3 ((disc* or disk* or intervertebra* or inter-vertebra*) adj5 (disease* or hernia* or protrud* or protrus* or prolaps* or slip* or displace* or chondrosis)).af. 252

4 (radiculopath* or radiculitis or polyradiculopath* or polyradiculitis or myelopath* or neuromyelopath* or osteophy* or spondylot* or retrolisthes*).af. 2

5 ((spine*1 or spinal or cord*1) adj5 (compress* or disease* or disorder*)).af. 5

6 ((spine*1 or spinal) adj5 stenos*).af. 0

7 (spondylodes* or arthrodes* or syndes* or ((spinal or spine*1 or vertebra*) adj5 fus*)).af. 1

8 ((anterior or oblique or psoas or prepsoas or interbody) adj5 (fus* or infus*)).af. 0

9 or/1-8 334

10 (lumbar or lumbalis).af. 8

11 lumbo*.af. 1

12 or/10-11 9

13 9 and 12 2

14 (OLIF51 or OLIF25 or OLIF or OLLIF* or ALIF).af. 2

15 (lumbar adj3 fus*).af. 0

16 or/13-15 4

17 (dibotermin alfa or dibotermin alpha or dibotermine alfa or dibotermine alpha).af. 0

18 ((bone morphogen* or osteogen* or bone morphologic or bone morphological) adj3 (protein* or factor* or polypeptide* or poly-peptide*)).af. 0

19 (bmp or bmp2 or rhbmp or rhbmp2).af. 39

20 ((iliac or ilium or ilial) adj5 (transplant* or graft* or autograft* or allograft* or autotransplant* or homotransplant* or heterotransplant*)).af. 0

21 (bone* adj5 (transplant* or graft* or autograft* or allograft* or autotransplant* or homotransplant* or heterotransplant* or cage* or scaffold* or demineraliz* or demineralis* or regenerat* or matrix or matrices or decalcif* or reimplant* or substitut* or replac*)).af. 23

22 (ICBG or ICBGs).af. 1

23 (osteotransplant* or osteo-transplant*).af. 0

24 (osteograft* or osteo-graft*).af. 0

25 (osteoconduct* or osteo-conduct*).af. 0

26 (osteoinduct* or osteo-induct*).af. 0

27 (inductos* or induct os or induct ostm or induct osr or infuse*2 or truscient* or nebotermin* or 246539-15-1 or T472P45MG6).af. 164

28 (i-factor* or mastergraft* or vitoss* or actifuse* or bonus* or chronos* or fibergraft* or magnetos* or grafton* or magnifuse* or DBF* or DBX* or osteosurge* or puros* or stagraft* or intergrow* or allocraft* or vesuvius* or trinity* or osteocel* or vivigen* or bonus triad*).af. 5561

29 or/17-28 5788

30 16 and 29 0

31 limit 30 to (yr="2000 -Current" and english) 0

1. **Source:** **ClinicalTrials.gov**

Interface / URL: https://clinicaltrials.gov/ct2/home

Database coverage dates: Information not found. ClinicalTrials.gov was created as a result of the Food and Drug Administration Modernization Act of 1997 (FDAMA). The site was made available to the public in February 2000.

Search date: 28 February 2023

Retrieved records: 1120

Search strategy:

The following eight searches were conducted separately.

All search terms were entered using the Expert search interface at this URL: <https://clinicaltrials.gov/ct2/results/refine?show_xprt=Y>.

The results from each search were downloaded as individual sets. The total number of records retrieved represents the sum of all searches, and includes duplicates caused by the same record being retrieved in each search.

Search 1

((lumbar OR lumbalis OR lumbosacral OR lumbosacrum OR lumborum OR lumbovertebral OR lumbovertebra OR lumboverterbrae) AND (((disc OR discs OR disk OR disks OR intervertebra OR intervertebrae OR intervertebral OR inter-vertebra OR inter-vertebrae OR inter-vertebral) AND (degeneration OR degenerations OR degenerated OR degenerating OR degenerate OR degenerates OR degenerative OR degrade OR degraded OR degrades OR degradation OR degrading OR disease OR diseases OR hernia OR hernias OR herniation OR herniated OR herniations OR protrude OR protrudes OR protruding OR protruded OR protrusion OR protrusions OR prolapse OR prolapsed OR prolapsing OR prolapses OR slip OR slips OR slipped OR slipping OR displace OR displaces OR displacement OR displacements OR displacing OR displaced OR chondrosis)) OR ((spine OR spines OR spinal OR cord OR cords) AND (compress OR compresses OR compressed OR compressing OR compression OR compressions OR disease OR diseases OR disorder OR disorders)) OR ((spine OR spines OR spinal) AND (stenosis OR stenoses)) OR ((anterior OR oblique OR psoas OR prepsoas OR interbody OR spine OR spines OR spinal OR vertebra OR vertebral OR vertebrae) AND (fusion OR fusing OR fuse OR fused OR fusions OR infusion OR infusing OR infuse OR infused OR infusions)) OR (spondylosis OR spondyloses OR spondylolysis OR spondylolyses OR spondylolisthesis OR spondylolistheses OR discopathy OR discopathies OR diskopathy OR diskopathies OR spondylochondrosis OR spondylochondroses OR spondylo-chondrosis OR spondylo-chondroses OR spondylodiscitis OR spondylo-discitis OR spondylodiskitis OR spondylo-diskitis OR spondylo-listhesis OR spondylo-listheses OR spondylo-lysthesis OR spondylo-lystheses OR olisthesis OR olistheses OR radiculopathy OR radiculopathies OR radiculitis OR polyradiculopathy OR polyradiculopathies OR polyradiculitis OR myelopathy OR myelopathies OR neuromyelopathy OR neuromyelopathies OR osteophyte OR osteophytes OR spondylotic OR spondylotics OR retrolisthesis OR retrolistheses OR spondylodesis OR spondylodeses OR arthrodesis OR arthrodesis OR syndesis OR syndeses))) AND ("dibotermin alfa" OR "dibotermin alpha" OR "dibotermine alfa" OR "dibotermine alpha" OR bmp OR bmp2 OR rhbmp OR rhbmp2 OR ICBG OR ICBGs OR osteotransplant OR osteotransplants OR osteotransplantation OR osteotransplantations OR osteotransplanted OR osteotransplanting OR osteo-transplant OR osteo-transplants OR osteo-transplantation OR osteo-transplantations OR osteo-transplanted OR osteo-transplanting OR osteograft OR osteografts OR osteografting OR osteografted OR osteo-graft OR osteo-grafts OR osteo-grafting OR osteo-grafted OR osteoconduction OR osteoconductor OR osteoconductors OR osteoconducting OR osteoconducted OR osteo-conduction OR osteo-conductor OR osteo-conductors OR osteo-conducting OR osteo-conducted OR osteoinduction OR osteoinductor OR osteoinductors OR osteoinducting OR osteoinducted OR osteo-induction OR osteo-inductor OR osteo-inductors OR osteo-inducting OR osteo-inducted OR inductos OR inductosr OR inductostm OR induct OR infuse OR infuser OR infusetm OR truscient OR truscientr OR truscienttm OR nebotermin OR neboterminr OR nebotermintm OR 246539-15-1 OR T472P45MG6 OR i-factor OR i-factorr OR i-factortm OR mastergraft OR mastergraftr OR mastergrafttm OR vitoss OR vitossr OR vitosstm OR actifuse OR actifuser OR actifusetm OR bonus OR bonusr OR bonustm OR chronos OR chronosr OR chronostm OR fibergraft OR fibergraftr OR fibergrafttm OR magnetos OR magnetosr OR magnetostm OR grafton OR graftonr OR graftontm OR magnifuse OR magnifuser OR magnifusetm OR DBF OR DBFr OR DBFtm OR DBX OR DBXr OR DBXtm OR osteosurge OR osteosurger OR osteosurgetm OR puros OR purosr OR purostm OR stagraft OR stagraftr OR stagrafttm OR intergrow OR intergrowr OR intergrowtm OR allocraft OR allocraftr OR allocrafttm OR vesuvius OR vesuviusr OR vesuviustm OR trinity OR trinityr OR trinitytm OR osteocel OR osteocelr OR osteoceltm OR vivigen OR vivigenr OR vivigentm OR "bonus triad" OR "bonus triadr" OR "bonus triadtm")

= 86 studies

Search 2

((lumbar OR lumbalis OR lumbosacral OR lumbosacrum OR lumborum OR lumbovertebral OR lumbovertebra OR lumboverterbrae) AND (((disc OR discs OR disk OR disks OR intervertebra OR intervertebrae OR intervertebral OR inter-vertebra OR inter-vertebrae OR inter-vertebral) AND (degeneration OR degenerations OR degenerated OR degenerating OR degenerate OR degenerates OR degenerative OR degrade OR degraded OR degrades OR degradation OR degrading OR disease OR diseases OR hernia OR hernias OR herniation OR herniated OR herniations OR protrude OR protrudes OR protruding OR protruded OR protrusion OR protrusions OR prolapse OR prolapsed OR prolapsing OR prolapses OR slip OR slips OR slipped OR slipping OR displace OR displaces OR displacement OR displacements OR displacing OR displaced OR chondrosis)) OR ((spine OR spines OR spinal OR cord OR cords) AND (compress OR compresses OR compressed OR compressing OR compression OR compressions OR disease OR diseases OR disorder OR disorders)) OR ((spine OR spines OR spinal) AND (stenosis OR stenoses)) OR ((anterior OR oblique OR psoas OR prepsoas OR interbody OR spine OR spines OR spinal OR vertebra OR vertebral OR vertebrae) AND (fusion OR fusing OR fuse OR fused OR fusions OR infusion OR infusing OR infuse OR infused OR infusions)) OR (spondylosis OR spondyloses OR spondylolysis OR spondylolyses OR spondylolisthesis OR spondylolistheses OR discopathy OR discopathies OR diskopathy OR diskopathies OR spondylochondrosis OR spondylochondroses OR spondylo-chondrosis OR spondylo-chondroses OR spondylodiscitis OR spondylo-discitis OR spondylodiskitis OR spondylo-diskitis OR spondylo-listhesis OR spondylo-listheses OR spondylo-lysthesis OR spondylo-lystheses OR olisthesis OR olistheses OR radiculopathy OR radiculopathies OR radiculitis OR polyradiculopathy OR polyradiculopathies OR polyradiculitis OR myelopathy OR myelopathies OR neuromyelopathy OR neuromyelopathies OR osteophyte OR osteophytes OR spondylotic OR spondylotics OR retrolisthesis OR retrolistheses OR spondylodesis OR spondylodeses OR arthrodesis OR arthrodesis OR syndesis OR syndeses))) AND (("bone morphogenetic" OR "bone morphogenetics" OR "bone morphogenic" OR "bone morphogenics" OR "bone morphogenesis" OR osteogenesis OR osteogenic OR osteogenics OR osteogenetic OR osteogenetics OR "bone morphologic" OR "bone morphological") AND (protein OR proteins OR factor OR factors OR polypeptide OR polypeptides OR poly-peptide OR poly-peptides))

= 66 studies

Search 3

((lumbar OR lumbalis OR lumbosacral OR lumbosacrum OR lumborum OR lumbovertebral OR lumbovertebra OR lumboverterbrae) AND (((disc OR discs OR disk OR disks OR intervertebra OR intervertebrae OR intervertebral OR inter-vertebra OR inter-vertebrae OR inter-vertebral) AND (degeneration OR degenerations OR degenerated OR degenerating OR degenerate OR degenerates OR degenerative OR degrade OR degraded OR degrades OR degradation OR degrading OR disease OR diseases OR hernia OR hernias OR herniation OR herniated OR herniations OR protrude OR protrudes OR protruding OR protruded OR protrusion OR protrusions OR prolapse OR prolapsed OR prolapsing OR prolapses OR slip OR slips OR slipped OR slipping OR displace OR displaces OR displacement OR displacements OR displacing OR displaced OR chondrosis)) OR ((spine OR spines OR spinal OR cord OR cords) AND (compress OR compresses OR compressed OR compressing OR compression OR compressions OR disease OR diseases OR disorder OR disorders)) OR ((spine OR spines OR spinal) AND (stenosis OR stenoses)) OR ((anterior OR oblique OR psoas OR prepsoas OR interbody OR spine OR spines OR spinal OR vertebra OR vertebral OR vertebrae) AND (fusion OR fusing OR fuse OR fused OR fusions OR infusion OR infusing OR infuse OR infused OR infusions)) OR (spondylosis OR spondyloses OR spondylolysis OR spondylolyses OR spondylolisthesis OR spondylolistheses OR discopathy OR discopathies OR diskopathy OR diskopathies OR spondylochondrosis OR spondylochondroses OR spondylo-chondrosis OR spondylo-chondroses OR spondylodiscitis OR spondylo-discitis OR spondylodiskitis OR spondylo-diskitis OR spondylo-listhesis OR spondylo-listheses OR spondylo-lysthesis OR spondylo-lystheses OR olisthesis OR olistheses OR radiculopathy OR radiculopathies OR radiculitis OR polyradiculopathy OR polyradiculopathies OR polyradiculitis OR myelopathy OR myelopathies OR neuromyelopathy OR neuromyelopathies OR osteophyte OR osteophytes OR spondylotic OR spondylotics OR retrolisthesis OR retrolistheses OR spondylodesis OR spondylodeses OR arthrodesis OR arthrodesis OR syndesis OR syndeses))) AND ((iliac OR ilium OR ilial OR bone OR bones) AND (transplant OR transplants OR transplanted OR transplanting OR transplantation OR transplantations OR graft OR grafts OR grafted OR grafting OR autograft OR autografts OR autografted OR autografting OR allograft OR allografts OR allografted OR allografting OR autotransplant OR autotransplanted OR autotransplantation OR autotransplantation s OR autotransplants OR autotransplanting OR homotransplant OR homotransplanted OR homotransplantation OR homotransplantation s OR homotransplants OR homotransplanting OR heterotransplant OR heterotransplanted OR heterotransplantation OR heterotransplantations OR heterotransplants OR heterotransplanting))

= 199 studies

Search 4

((lumbar OR lumbalis OR lumbosacral OR lumbosacrum OR lumborum OR lumbovertebral OR lumbovertebra OR lumboverterbrae) AND (((disc OR discs OR disk OR disks OR intervertebra OR intervertebrae OR intervertebral OR inter-vertebra OR inter-vertebrae OR inter-vertebral) AND (degeneration OR degenerations OR degenerated OR degenerating OR degenerate OR degenerates OR degenerative OR degrade OR degraded OR degrades OR degradation OR degrading OR disease OR diseases OR hernia OR hernias OR herniation OR herniated OR herniations OR protrude OR protrudes OR protruding OR protruded OR protrusion OR protrusions OR prolapse OR prolapsed OR prolapsing OR prolapses OR slip OR slips OR slipped OR slipping OR displace OR displaces OR displacement OR displacements OR displacing OR displaced OR chondrosis)) OR ((spine OR spines OR spinal OR cord OR cords) AND (compress OR compresses OR compressed OR compressing OR compression OR compressions OR disease OR diseases OR disorder OR disorders)) OR ((spine OR spines OR spinal) AND (stenosis OR stenoses)) OR ((anterior OR oblique OR psoas OR prepsoas OR interbody OR spine OR spines OR spinal OR vertebra OR vertebral OR vertebrae) AND (fusion OR fusing OR fuse OR fused OR fusions OR infusion OR infusing OR infuse OR infused OR infusions)) OR (spondylosis OR spondyloses OR spondylolysis OR spondylolyses OR spondylolisthesis OR spondylolistheses OR discopathy OR discopathies OR diskopathy OR diskopathies OR spondylochondrosis OR spondylochondroses OR spondylo-chondrosis OR spondylo-chondroses OR spondylodiscitis OR spondylo-discitis OR spondylodiskitis OR spondylo-diskitis OR spondylo-listhesis OR spondylo-listheses OR spondylo-lysthesis OR spondylo-lystheses OR olisthesis OR olistheses OR radiculopathy OR radiculopathies OR radiculitis OR polyradiculopathy OR polyradiculopathies OR polyradiculitis OR myelopathy OR myelopathies OR neuromyelopathy OR neuromyelopathies OR osteophyte OR osteophytes OR spondylotic OR spondylotics OR retrolisthesis OR retrolistheses OR spondylodesis OR spondylodeses OR arthrodesis OR arthrodesis OR syndesis OR syndeses))) AND ((bone OR bones) AND (cage OR cages OR scaffold OR scaffolds OR scaffolding OR scaffolded OR demineralized OR demineralization OR demineralizing OR demineralised OR demineralisation OR demineralising OR regenerate OR regenerated OR regeneration OR regenerating OR matrix OR matrices OR decalcified OR decalcify OR decalcification OR decalcifies OR decalcifying OR reimplantation OR reimplanted OR reimplant OR reimplants OR reimplanting OR substitute OR substitutes OR substitution OR substitutions OR substituted OR substituting OR replace OR replacement OR replacements OR replacing OR replaced OR replaces))

= 342 studies

Search 5

((OLIF51 OR OLIF25 OR OLIF OR OLLIF OR OLLIF51 OR OLLIF25 OR ALIF) OR (lumbar AND (fusion OR fusing OR fuse OR fused OR fusions OR infusion OR infusing OR infuse OR infused OR infusions))) AND ("dibotermin alfa" OR "dibotermin alpha" OR "dibotermine alfa" OR "dibotermine alpha" OR bmp OR bmp2 OR rhbmp OR rhbmp2 OR ICBG OR ICBGs OR osteotransplant OR osteotransplants OR osteotransplantation OR osteotransplantations OR osteotransplanted OR osteotransplanting OR osteo-transplant OR osteo-transplants OR osteo-transplantation OR osteo-transplantations OR osteo-transplanted OR osteo-transplanting OR osteograft OR osteografts OR osteografting OR osteografted OR osteo-graft OR osteo-grafts OR osteo-grafting OR osteo-grafted OR osteoconduction OR osteoconductor OR osteoconductors OR osteoconducting OR osteoconducted OR osteo-conduction OR osteo-conductor OR osteo-conductors OR osteo-conducting OR osteo-conducted OR osteoinduction OR osteoinductor OR osteoinductors OR osteoinducting OR osteoinducted OR osteo-induction OR osteo-inductor OR osteo-inductors OR osteo-inducting OR osteo-inducted OR inductos OR inductosr OR inductostm OR induct OR infuse OR infuser OR infusetm OR truscient OR truscientr OR truscienttm OR nebotermin OR neboterminr OR nebotermintm OR 246539-15-1 OR T472P45MG6 OR i-factor OR i-factorr OR i-factortm OR mastergraft OR mastergraftr OR mastergrafttm OR vitoss OR vitossr OR vitosstm OR actifuse OR actifuser OR actifusetm OR bonus OR bonusr OR bonustm OR chronos OR chronosr OR chronostm OR fibergraft OR fibergraftr OR fibergrafttm OR magnetos OR magnetosr OR magnetostm OR grafton OR graftonr OR graftontm OR magnifuse OR magnifuser OR magnifusetm OR DBF OR DBFr OR DBFtm OR DBX OR DBXr OR DBXtm OR osteosurge OR osteosurger OR osteosurgetm OR puros OR purosr OR purostm OR stagraft OR stagraftr OR stagrafttm OR intergrow OR intergrowr OR intergrowtm OR allocraft OR allocraftr OR allocrafttm OR vesuvius OR vesuviusr OR vesuviustm OR trinity OR trinityr OR trinitytm OR osteocel OR osteocelr OR osteoceltm OR vivigen OR vivigenr OR vivigentm OR "bonus triad" OR "bonus triadr" OR "bonus triadtm")

=68 studies

Search 6

((OLIF51 OR OLIF25 OR OLIF OR OLLIF OR OLLIF51 OR OLLIF25 OR ALIF) OR (lumbar AND (fusion OR fusing OR fuse OR fused OR fusions OR infusion OR infusing OR infuse OR infused OR infusions))) AND (("bone morphogenetic" OR "bone morphogenetics" OR "bone morphogenic" OR "bone morphogenics" OR "bone morphogenesis" OR osteogenesis OR osteogenic OR osteogenics OR osteogenetic OR osteogenetics OR "bone morphologic" OR "bone morphological") AND (protein OR proteins OR factor OR factors OR polypeptide OR polypeptides OR poly-peptide OR poly-peptides))

=39 studies

Search 7

((OLIF51 OR OLIF25 OR OLIF OR OLLIF OR OLLIF51 OR OLLIF25 OR ALIF) OR (lumbar AND (fusion OR fusing OR fuse OR fused OR fusions OR infusion OR infusing OR infuse OR infused OR infusions))) AND ((iliac OR ilium OR ilial OR bone OR bones) AND (transplant OR transplants OR transplanted OR transplanting OR transplantation OR transplantations OR graft OR grafts OR grafted OR grafting OR autograft OR autografts OR autografted OR autografting OR allograft OR allografts OR allografted OR allografting OR autotransplant OR autotransplanted OR autotransplantation OR autotransplantation s OR autotransplants OR autotransplanting OR homotransplant OR homotransplanted OR homotransplantation OR homotransplantation s OR homotransplants OR homotransplanting OR heterotransplant OR heterotransplanted OR heterotransplantation OR heterotransplantations OR heterotransplants OR heterotransplanting))

= 154 studies

Search 8

((OLIF51 OR OLIF25 OR OLIF OR OLLIF OR OLLIF51 OR OLLIF25 OR ALIF) OR (lumbar AND (fusion OR fusing OR fuse OR fused OR fusions OR infusion OR infusing OR infuse OR infused OR infusions))) AND ((bone OR bones) AND (cage OR cages OR scaffold OR scaffolds OR scaffolding OR scaffolded OR demineralized OR demineralization OR demineralizing OR demineralised OR demineralisation OR demineralising OR regenerate OR regenerated OR regeneration OR regenerating OR matrix OR matrices OR decalcified OR decalcify OR decalcification OR decalcifies OR decalcifying OR reimplantation OR reimplanted OR reimplant OR reimplants OR reimplanting OR substitute OR substitutes OR substitution OR substitutions OR substituted OR substituting OR replace OR replacement OR replacements OR replacing OR replaced OR replaces))

= 166 studies

1. **Source:** **WHO International Clinical Trials Registry Portal (ICTRP)**

Interface / URL: https://trialsearch.who.int/

Database coverage dates: Information not found. On the date of search, files had been imported from data providers between May 2022 and February 2023

Search date: 27 February 2023

Retrieved records: 194

Search strategy:

The following four searches were conducted separately using the search interface at the above URL. 'Without Synonyms' was selected for all searches.

Searches were split into the following four separate searches to avoid issues experienced with the interface timing out when using long search strings.

The results from each search were downloaded as an individual set. The total number of records retrieved represents the sum of all searches, and includes duplicates caused by the same record being retrieved in each search.

Search 1:

(lumbar OR lumbalis OR lumbo* OR OLIF51 OR OLIF25 OR OLIF OR OLLIF* OR ALIF) AND (("dibotermin alfa" OR "dibotermin alpha" OR "dibotermine alfa" OR "dibotermine alpha") OR (("bone morphogen*" OR osteogen* OR "bone morphologic" OR "bone morphological") AND (protein* OR factor* OR polypeptide* OR poly-peptide*)) OR (bmp OR bmp2 OR rhbmp OR rhbmp2))

= 26 trials

Search 2:

(lumbar OR lumbalis OR lumbo* OR OLIF51 OR OLIF25 OR OLIF OR OLLIF* OR ALIF) AND (((iliac OR ilium OR ilial OR bone*) AND (transplant* OR graft* OR autograft* OR allograft* OR autotransplant* OR homotransplant* OR heterotransplant*)) OR (bone* AND (cage* OR scaffold* OR demineraliz* OR demineralis* OR regenerat* OR matrix OR matrices OR decalcif* OR reimplant* OR substitut* OR replac*)))

= 113 trials

Search 3:

(lumbar OR lumbalis OR lumbo* OR OLIF51 OR OLIF25 OR OLIF OR OLLIF* OR ALIF) AND (ICBG OR ICBGs OR osteotransplant* OR osteo-transplant* OR osteograft* OR osteo-graft* OR osteoconduct* OR osteo-conduct* OR osteoinduct* OR osteo-induct*)

= 2 trials

Search 4:

(lumbar OR lumbalis OR lumbo* OR OLIF51 OR OLIF25 OR OLIF OR OLLIF* OR ALIF) AND ((inductos* OR "induct os" OR "induct ostm" OR "induct osr" OR infuse* OR truscient* OR nebotermin* OR 246539-15-1 OR T472P45MG6 OR i-factor* OR mastergraft* OR vitoss* OR actifuse* OR bonus* OR chronos* OR fibergraft* OR magnetos* OR grafton* OR magnifuse* OR DBF* OR DBX* OR osteosurge* OR puros* OR stagraft* OR intergrow* OR allocraft* OR vesuvius* OR trinity* OR osteocel* OR vivigen* OR "bonus triad*"))

= 53 trials

1. **Source:** **National Institute for Health and Care Excellence (NICE) webpages**

Interface / URL: https://www.nice.org.uk/

Database coverage dates: n/a

Search date: 28 February 2023

Retrieved records: 1

Search strategy:

The NICE website was browsed via links to "Guidance" then "Conditions and diseases" then "Musculoskeletal conditions" and finally "Spinal conditions". The 47 published products were rapidly screened by the Information Specialist for relevance to the review. One document was retrieved.

As a check the site-wide search interface was used at: <https://www.nice.org.uk/>. The term "fusion" was entered, and all results were rapidly screened by the Information Specialist for relevance to the review. Nothing further was identified.

1. **Source:** **Canadian Agency for Drugs and Technologies in Health (CADTH) webpages**

Interface / URL: https://www.cadth.ca/

Database coverage dates: n/a

Search date: 28 February 2023

Retrieved records: 2

Search strategy:

The site-wide search interface was used at: <https://www.cadth.ca/>. Separate searches were conducted on each term shown below. Results were rapidly screened by the Information Specialist for relevance to the review. PDFs of relevant documents were retrieved for further assessment. Duplicate documents were not retrieved.

OLIF51 (0 results)

OLIF25 (0 results)

OLIF (1 results)

OLLIF (0 results)

OLLIF51 (0 results)

OLLIF25 (0 results)

ALIF (0 results)

lumbar (87 results)

lumbalis (0 results)

lumbosacral (10 results)

lumbosacrum (0 results)

lumborum (1 results)

lumbovertebral (0 results)

lumbovertebra (0 results)

lumboverterbrae (0 results)

spondylosis (9 results)

spondyloses (0 results)

spondylolysis (3 results)

spondylolyses (0 results)

spondylolisthesis (7 results)

spondylolistheses (0 results)

discopathy (0 results)

discopathies (0 results)

diskopathy (0 results)

diskopathies (0 results)

spondylochondrosis (0 results)

spondylochondroses (0 results)

spondylo-chondrosis (0 results)

spondylo-chondroses (0 results)

spondylodiscitis (2 results)

spondylo-discitis (0 results)

spondylodiskitis (0 results)

spondylo-diskitis (0 results)

spondylo-listhesis (0 results)

spondylo-listheses (0 results)

spondylo-lysthesis (0 results)

spondylo-lystheses (0 results)

olisthesis (0 results)

olistheses (0 results)

radiculopathy (21 results)

radiculopathies (21 results)

radiculitis (1 results)

polyradiculopathy (1 results)

polyradiculopathies (1 results)

polyradiculitis (1 results)

myelopathy (8 results)

myelopathies (8 results)

neuromyelopathy (0 results)

neuromyelopathies (0 results)

osteophyte (8 results)

osteophytes (8 results)

spondylotic (0 results)

spondylotics (0 results)

retrolisthesis (1 results)

retrolistheses (0 results)

spondylodesis (0 results)

spondylodeses (0 results)

arthrodesis (3 results)

arthrodeses (0 results)

syndesis (0 results)

syndeses (0 results)

disc (100 results)

discs (100 results)

disk (20 results)

disks (20 results)

intervertebra (0 results)

intervertebrae (0 results)

intervertebral (27 results)

inter-vertebra (0 results)

inter-vertebrae (0 results)

inter-vertebral (0 results)

psoas (0 results)

prepsoas (0 results)

interbody (3 results)

anterior (118 results)

oblique (7 results)

fuse (25 results)

fused (25 results)

fusion (141 results)

fusing (25 results)

fusions (141 results)

spine fusion (38 results)

spinal fusion (49 results)

spine stenosis (27 results)

spinal stenosis (40 results)

spine compression (33 results)

spinal compression (62 results)

spine disease (145 results)

spinal disease (197 results)

spine disorder (117 results)

spinal disorder (167 results)

cord compression (44 results)

cord disease (167 results)

cord disorder (136 results)

1. **Source:** **Institute for Clinical and Economic Review webpages**

Interface / URL: https://icer-review.org/

Database coverage dates: n/a

Search date: 27 February 2023

Retrieved records: 0

Search strategy:

At the following URL: <https://icer.org/explore-our-research/assessments/>

The filter for 'Diseases & Conditions' was checked for relevant topics - none were identified.

1. **Source:** **European Medicines Agency (EMA) medicines webpages**

Interface / URL: https://www.ema.europa.eu/en

Database coverage dates: n/a

Search date: 28 February 2023

Retrieved records: 9

Search strategy:

The following documents for the drugs of interest were sought: 'EPAR - Public Assessment Report'. Associated documents with variant titles were also retrieved (e.g. 'EPAR - Assessment Report', 'EPAR - Assessment Report – Variation', 'EPAR procedural steps taken', EPAR – Scientific Conclusions', etc.)

Documents were retrieved if found under the headings 'Initial marketing-authorisation documents' and ' Changes since initial authorisation of medicine'.

The search interface at the following URL was used: <https://www.ema.europa.eu/en/medicines>.

Separate searches were conducted on each drug name shown below:

"Inductos"

"truscient"

"nebotermin"

Each result was assessed for correct drug and correct indication.

For relevant results, documents under 'Assessment history' were checked to identify the documents of interest noted above. Duplicate documents were not retrieved.

Nine documents were retrieved for further assessment.

# Appendix C: Resources Searched

**Table C1: Resources searched**

| Resource | Interface / URL |
| --- | --- |
| Databases | |
| MEDLINE(R) ALL | OvidSP |
| Embase | OvidSP |
| Cochrane Database of Systematic Reviews (CDSR) | Cochrane Library/Wiley |
| Cochrane Central Register of Controlled Trials (CENTRAL) | Cochrane Library/Wiley |
| International HTA Database | https://database.inahta.org/ |
| NHS Economic Evaluation Database (NHS EED) | https://www.crd.york.ac.uk/CRDWeb/HomePage.asp |
| Econlit | OvidSP |
| Trials Registers | |
| ClinicalTrials.gov | https://clinicaltrials.gov/ |
| WHO International Clinical Trials Registry Platform (ICTRP) | https://trialsearch.who.int/ |
| HTA / regulatory agency webpages | |
| National Institute for Health and Care Excellence (NICE) webpages | https://www.nice.org.uk/ |
| Canadian Agency for Drugs and Technologies in Health (CADTH) webpages | https://www.cadth.ca/ |
| Institute for Clinical and Economic Review (ICER) webpages | https://icer-review.org/ |
| European Medicines Agency (EMA) medicines webpages | https://www.ema.europa.eu/ |
| Reference checking | |
| Included studies list of systematic review checking | Included studies list of any retrieved relevant systematic reviews published between 2018 and 2023 |

# Appendix D: Excluded Studies

Table D.1: Excluded studies (n=314)

|  | Reference | Ref ID | Exclusion reason |
| --- | --- | --- | --- |
| 1 | Ackerman SJ, Mafilios MS, Polly DW, Jr. Economic evaluation of bone morphogenetic protein versus autogenous iliac crest bone graft in single-level anterior lumbar fusion: an evidence-based modeling approach. Spine. 2002.27(16 Suppl 1):S94-9. | 1768 | Ineligible outcomes |
| 2 | Acosta FL, Cloyd JM, Aryan HE, Ames CP. Patient satisfaction and radiographic outcomes after lumbar spinal fusion without iliac crest bone graft or transverse process fusion. J Clin Neurosci. 2009.16(9):1184-7. doi: https://dx.doi.org/10.1016/j.jocn.2008.12.006 | 1328 | Off label use of rhBMP |
| 3 | Ahlquist S, Thommen R, Park HY, Sheppard W, James K, Lord E, et al. Implications of sagittal alignment and complication profile with stand-alone anterior lumbar interbody fusion versus anterior posterior lumbar fusion. J Spine Surg. 2020.6(4):659-69. doi: https://dx.doi.org/10.21037/jss-20-595 | 130 | Mixed interventions |
| 4 | Alexander D, Oxner W, Soroceanu S, Kelly A, Shakespeare D. Prospective randomized controlled study comparing a DBM-CaSO4 composite graft and bone marrow aspirate with autologous iliac crest bone graft in 1-level and 2-level lumbar and lumbosacral spinal fusions. Can J Surg. 2008.(3 Suppl):S7. | 3637 | Ineligible patient population |
| 5 | Alimi M, Navarro-Ramirez R, Parikh K, Njoku I, Hofstetter CP, Tsiouris AJ, Hartl R. Radiographic and clinical outcome of silicate-substituted calcium phosphate (Si-CaP) ceramic bone graft in spinal fusion procedures. Clin Spine Surg. 2017.30(6):E845-E52. doi: https://dx.doi.org/10.1097/BSD.0000000000000432 | 574 | Ineligible patient population |
| 6 | Allain J, Delecrin J, Beaurain J, Poignard A, Vila T, Flouzat-Lachaniette CH. Stand-alone ALIF with integrated intracorporeal anchoring plates in the treatment of degenerative lumbar disc disease: a prospective study on 65 cases. Eur Spine J. 2014.23(10):2136-43. doi: 10.1007/s00586-014-3364-1 | 4957 | Off label use of rhBMP |
| 7 | Anderson DG, Sayadipour A, Shelby K, Albert TJ, Vaccaro AR, Weinstein MS. Anterior interbody arthrodesis with percutaneous posterior pedicle fixation for degenerative conditions of the lumbar spine. Eur Spine J. 2011.20(8):1323-30. doi: https://dx.doi.org/10.1007/s00586-011-1782-x | 1189 | Ineligible patient population |
| 8 | Andresen AK, Carreon LY, Overgaard S, Jacobsen MK, Andersen MO. Safety and reoperation rates in non-instrumented lumbar fusion surgery: Secondary report from a randomized controlled trial of ABM/P-15 vs allograft with minimum 5 years follow-up. Global Spine J. 2022.21925682221090924. doi: https://dx.doi.org/10.1177/21925682221090924 | 12 | Surgical route not reported |
| 9 | Anonymous. Bone morphogenetic protein (infuse bone graft). Med Lett Drugs Ther. 2009.51(1326-1327):99-100. | 2097 | Unobtainable |
| 10 | Artoss Inc. Rate of bony fusion using NanoBone® synthetic bone graft versus local autologous bone graft. Identifier: NCT04615260. In: ClinicalTrials.gov [internet]. Bethesda: US National Library of Medicine: 2021. Available from https://ClinicalTrials.gov/show/NCT04615260. | 3938 | Ineligible intervention |
| 11 | Aryan HE, Newman CB, Gold JJ, Acosta FL, Jr., Coover C, Ames CP. Percutaneous axial lumbar interbody fusion (AxiaLIF) of the L5-S1 segment: initial clinical and radiographic experience. Minim Invasive Neurosurg. 2008.51(4):225-30. doi: https://dx.doi.org/10.1055/s-2008-1080915 | 1398 | Ineligible intervention |
| 12 | Ashayeri K, Leon C, Tigchelaar S, Fatemi P, Follett M, Cheng I, et al. Single position lateral decubitus anterior lumbar interbody fusion (ALIF) and posterior fusion reduces complications and improves perioperative outcomes compared with traditional anterior-posterior lumbar fusion. Spine J. 2022.22(3):419-28. doi: https://dx.doi.org/10.1016/j.spinee.2021.09.009 | 3058 | Mixed interventions |
| 13 | Assiri I, du Plessis S, Hurlbert J, Hu R, Salo P, Whittaker T. A prospective randomized clinical study comparing instrumented lumbar fusion rates of Recombinant Human Bone Morphogenic Protein-2 (rhBMP-2) with autogenous iliac crest bone graft in patients with symptomatic degenerative disc disease. Can J Surg. 2004.(Suppl 4):7‐8. | 3571 | conference abstract |
| 14 | Aunoble S, Hoste D, Donkersloot P, Liquois F, Basso Y, Le Huec J-C. Video-assisted ALIF with cage and anterior plate fixation for L5-S1 spondylolisthesis. J Spinal Disord Tech. 2006.19(7):471-6. | 1511 | Ineligible patient population |
| 15 | Aziyo Biologics Inc. A study evaluating 3-level OLIF spine fusion. Identifier: NCT03896347. In: ClinicalTrials.gov [internet]. Bethesda: US National Library of Medicine: 2019. Available from https://ClinicalTrials.gov/show/NCT03896347. | 3747 | Ineligible patient population |
| 16 | Aziyo Biologics Inc. ViBone in cervical and lumbar spine fusion. Identifier: NCT03425682. In: ClinicalTrials.gov [internet]. Bethesda: US National Library of Medicine: 2018. Available from https://ClinicalTrials.gov/show/NCT03425682. | 3941 | Ineligible patient population |
| 17 | Bains R, Mitsunaga L, Kardile M, Chen Y, Guppy K, Harris J, Paxton E. Bone morphogenetic protein (BMP-2) usage and cancer correlation: An analysis of 10,416 spine fusion patients from a multi-center spine registry. J Clin Neurosci. 2017.43:214-19. doi: https://dx.doi.org/10.1016/j.jocn.2017.05.007 | 2096 | Surgical route not reported |
| 18 | Barbanti Brodano G, Griffoni C, Zanotti B, Gasbarrini A, Bandiera S, Ghermandi R, Boriani S. A post-market surveillance analysis of the safety of hydroxyapatite-derived products as bone graft extenders or substitutes for spine fusion. Eur Rev Med Pharmacol Sci. 2015.19(19):3548-55. | 738 | Ineligible intervention |
| 19 | Barnes B, Rodts GE, McLaughlin MR, Haid RW, Jr. Threaded cortical bone dowels for lumbar interbody fusion: over 1-year mean follow up in 28 patients. J Neurosurg. 2001.95(1 Suppl):1-4. | 1821 | <10 patients |
| 20 | Bassani R, Sirtori P, Morselli C, Cirullo A, Ciliberto R, Mangiavini L, Peretti GM. Simultaneous L5-S1 anterior lumbar interbody fusion and total hip arthroplasty through minimally invasive anterior approaches in hip-spine syndrome. J Biol Regul Homeost Agents. 2022.36(2):239-45. doi: https://dx.doi.org/10.23812/22-59-A | 3055 | Ineligible study design |
| 21 | Beckerman D, Esparza M, Lee SI, Berven SH, Bederman SS, Hu SS, et al. Cost analysis of single-level lumbar fusions. Global Spine J. 2020.10(1):39-46. doi: https://dx.doi.org/10.1177/2192568219853251 | 2288 | ALIF/OLIF not reported separately |
| 22 | Behrbalk E, Uri O, Parks RM, Musson R, Soh RCC, Boszczyk BM. Fusion and subsidence rate of stand alone anterior lumbar interbody fusion using PEEK cage with recombinant human bone morphogenetic protein-2. Eur Spine J. 2013.22(12):2869-75. doi: https://dx.doi.org/10.1007/s00586-013-2948-5 | 903 | Ineligible patient population |
| 23 | Beschloss AM, DiCindio CM, Lombardi JS, Shillingford JN, Laratta JL, Holderread B, et al. The rise and fall of bone morphogenetic protein 2 throughout the United States. Clin Spine Surg. 2022.35(6):264-69. doi: https://dx.doi.org/10.1097/BSD.0000000000001301 | 205 | ALIF/OLIF not reported separately |
| 24 | Beutler WJ, Peppelman WC, Jr. Anterior lumbar fusion with paired BAK standard and paired BAK Proximity cages: subsidence incidence, subsidence factors, and clinical outcome. Spine J. 2003.3(4):289-93. doi: 10.1016/s1529-9430(03)00061-5 | 4958 | Ineligible intervention |
| 25 | BioSurface Engineering Technologies Inc. Pilot study to assess safety/prelimary effectiveness of prefix in subjects with degenerative disc disease (DDD) undergoing spine fusion surgery. Identifier: NCT00798239. In: ClinicalTrials.gov [internet]. Bethesda: US National Library of Medicine: 2008. Available from https://ClinicalTrials.gov/show/NCT00798239. | 3911 | Ineligible patient population |
| 26 | BioSurface Engineering Technologies Inc. Pilot study to assess safety/preliminary effectiveness of prefix in subjects with degenerative disc disease (DDD) undergoing spine fusion surgery. Identifier: NCT00798902. In: ClinicalTrials.gov [internet]. Bethesda: US National Library of Medicine: 2008. Available from https://ClinicalTrials.gov/show/NCT00798902. | 3909 | Ineligible patient population |
| 27 | Bioventus LLC. OSTEOAMP lumbar fusion intra-patient controlled study. Identifier: NCT05405374. In: ClinicalTrials.gov [internet]. Bethesda: US National Library of Medicine: 2021. Available from https://clinicaltrials.gov/show/NCT05405374. | 4846 | Ineligible patient population |
| 28 | Bioventus LLC. OSTEOAMP lumbar fusion study. Identifier: NCT05405374. In: ClinicalTrials.gov [internet]. Bethesda: US National Library of Medicine: 2022. Available from https://clinicaltrials.gov/show/NCT05405374. | 3408 | Ineligible patient population |
| 29 | Blumenthal S, McAfee PC, Guyer RD, Hochschuler SH, Geisler FH, Holt RT, et al. A prospective, randomized, multicenter Food and Drug Administration Investigational Device Exemptions study of lumbar total disc replacement with the CHARITETM artificial disc versus lumbar fusion - Part I: Evaluation of clinical outcomes. Spine. 2005.30(14):1565-75. doi: https://dx.doi.org/10.1097/01.brs.0000170587.32676.0e | 1938 | Ineligible intervention |
| 30 | Bone Therapeutics SA. Phase 2a study on allogeneic osteoblastic cells implantation in lumbar spinal fusion. Identifier: NCT02205138. In: ClinicalTrials.gov [internet]. Bethesda: US National Library of Medicine: 2014. Available from https://ClinicalTrials.gov/show/NCT02205138. | 3919 | Ongoing trial |
| 31 | Bozzio AE, Johnson CR, Fattor JA, Kleck CJ, Patel VV, Burger EL, et al. Stand-alone anterior lumbar interbody, transforaminal lumbar interbody, and anterior/posterior fusion: Analysis of fusion outcomes and costs. Orthopedics. 2018.41(5):e655-e62. doi: 10.3928/01477447-20180711-06 | 4966 | Off label use of rhBMP |
| 32 | Brara HS, Fessler RG. The role of anterior lumbar interbody allograft bone dowel fusion as an adjunct to posterior segmental lumbar fixation. Clin Neurosurg. 2000.47:528-33. | 1841 | Ineligible intervention |
| 33 | Brewster L, Trueger N, Schermer C, Ghanayem A, Santaniello J. Infraumbilical anterior retroperitoneal exposure of the lumbar spine in 128 consecutive patients. World J Surg. 2008.32(7):1414-9. doi: https://dx.doi.org/10.1007/s00268-007-9433-4 | 1431 | Mixed interventions |
| 34 | Bronheim RS, Cheung ZB, Phan K, White SJW, Kim JS, Cho SK. Anterior lumbar fusion: Differences in patient selection and surgical outcomes between neurosurgeons and orthopaedic surgeons. World Neurosurg. 2018.120:e221-e26. doi: https://dx.doi.org/10.1016/j.wneu.2018.08.034 | 461 | Mixed interventions |
| 35 | Buckland AJ, Ashayeri K, Leon C, Manning J, Eisen L, Medley M, et al. Single position circumferential fusion improves operative efficiency, reduces complications and length of stay compared with traditional circumferential fusion. Spine J. 2021.21(5):810-20. doi: https://dx.doi.org/10.1016/j.spinee.2020.11.002 | 3057 | Ineligible patient population |
| 36 | Burkus JK, Gornet MF, Schuler TC, Kleeman TJ, Zdeblick TA. Six-year outcomes of anterior lumbar interbody arthrodesis with use of interbody fusion cages and recombinant human bone morphogenetic protein-2: Reply. Journal of Bone and Joint Surgery. 2010.92(15):2615-16. | 3072 | Ineligible study design |
| 37 | Burkus JK, Sandhu HS, Gornet MF, Longley MC. Use of rhBMP-2 in combination with structural cortical allografts: clinical and radiographic outcomes in anterior lumbar spinal surgery. J Bone Joint Surg Am. 2005.87(6):1205-12. | 1590 | Off label use of rhBMP |
| 38 | Burkus JK, Sandhu HS, Gornet MF. Influence of rhBMP-2 on the healing patterns associated with allograft interbody constructs in comparison with autograft. Spine. 2006.31(7):775-81. | 1542 | Off label use of rhBMP |
| 39 | Buser Z, Brodke DS, Youssef JA, Rometsch E, Park J-B, Yoon ST, et al. Allograft versus demineralized bone matrix in instrumented and noninstrumented lumbar fusion: A systematic review. Global Spine J. 2018.8(4):396-412. doi: https://dx.doi.org/10.1177/2192568217735342 | 621 | SR to check |
| 40 | Buser Z, Hsieh P, Meisel H-J, Skelly AC, Brodt ED, Brodke DS, et al. Use of autologous stem cells in lumbar spinal fusion: A systematic review of current clinical evidence. Global Spine J. 2021.11(8):1281-98. doi: https://dx.doi.org/10.1177/2192568220973190 | 125 | SR to check |
| 41 | Cahill KS, Chi JH, Day A, Claus EB. Prevalence, complications, and hospital charges associated with use of bone-morphogenetic proteins in spinal fusion procedures. JAMA. 2009.302(1):58-66. doi: https://dx.doi.org/10.1001/jama.2009.956 | 1317 | ALIF/OLIF not reported separately |
| 42 | Cahill KS, Chi JH, Groff MW, McGuire K, Afendulis CC, Claus EB. Outcomes for single-level lumbar fusion: the role of bone morphogenetic protein. Spine. 2011.36(26):2354-62. doi: https://dx.doi.org/10.1097/BRS.0b013e31820bc9e5 | 1201 | ALIF/OLIF not reported separately |
| 43 | Carragee EJ, Mitsunaga KA, Hurwitz EL, Scuderi GJ. Retrograde ejaculation after anterior lumbar interbody fusion using rhBMP-2: a cohort controlled study. Spine J. 2011.11(6):511-6. doi: https://dx.doi.org/10.1016/j.spinee.2011.02.013 | 1182 | Off label use of rhBMP |
| 44 | CGBio. Evaluation of efficacy and safety of NOVOSIS, BONGROS, EXCELOS INJECT on the patient who underwent in oblique lumbar interbody fusion to treat degenerative spinal disease. Identifier: KCT0005328. In: Clinical Research Information Service (CRIS) [internet]. Cheongju: Korea Centers for Disease Control and Prevention (KCDC): 2020. Available from http://cris.nih.go.kr/cris/en/search/search_result_st01.jsp?seq=18588. | 4824 | Ongoing trial |
| 45 | Chen W-J, Tsai T-T, Chen L-H, Niu C-C, Lai P-L, Fu T-S, McCarthy K. The fusion rate of calcium sulfate with local autograft bone compared with autologous iliac bone graft for instrumented short-segment spinal fusion. Spine. 2005.30(20):2293-7. | 1567 | Ineligible intervention |
| 46 | Cheung KMC, Zhang YG, Lu DS, Luk KDK, Leong JCY. Reduction of disc space distraction after anterior lumbar interbody fusion with autologous iliac crest graft. Spine. 2003.28(13):1385-9. | 1708 | Ineligible intervention |
| 47 | Cheung MY, Cheung P. Oblique lumbar interbody fusion in management of lumbar degenerative spinal stenosis in Chinese population. JOTR. 2020.27(2):119-27. doi: https://dx.doi.org/10.1177/2210491720923058 | 2775 | Ineligible patient population |
| 48 | Chi JH, Nunley PD, Huang KT, Krag MH, Bydon M, Lavoie S, et al. Two-year outcomes from a prospective multicenter investigation device trial of a novel conformal mesh interbody fusion device. Int J Spine Surg. 2021.15(6):1103-14. doi: https://dx.doi.org/10.14444/8169 | 87 | Ineligible route of administration |
| 49 | Cho CB, Ryu KS, Park CK. Anterior lumbar interbody fusion with stand-alone interbody cage in treatment of lumbar intervertebral foraminal stenosis : comparative study of two different types of cages. J Korean Neurosurg Soc. 2010.47(5):352-7. doi: 10.3340/jkns.2010.47.5.352 | 4967 | Ineligible intervention |
| 50 | Chung NS, Jeon CH, Lee HD, Chung HW. Factors affecting disc angle restoration in oblique lateral interbody fusion at L5-S1. Spine J. 2021.21(12):2019-25. doi: https://dx.doi.org/10.1016/j.spinee.2021.07.016 | 2457 | Ineligible patient population |
| 51 | Chung NS, Lee HD, Jeon CH, Jeong S. Factors affecting slip reduction in oblique lumbar interbody fusion with posterior fixation for degenerative spondylolisthesis. Global Spine J. 2022.12(8):1786-91. doi: https://dx.doi.org/10.1177/2192568221989295 | 2458 | Ineligible patient population |
| 52 | Chung NS, Lee HD, Jeon CH. The impact of vertebral end plate lesions on the radiological outcome in oblique lateral interbody fusion. Global Spine J. 2021.11(8):1176-82. doi: https://dx.doi.org/10.1177/2192568220941447 | 3211 | Ineligible patient population |
| 53 | Comer GC, Smith MW, Hurwitz EL, Mitsunaga KA, Kessler R, Carragee EJ. Retrograde ejaculation after anterior lumbar interbody fusion with and without bone morphogenetic protein-2 augmentation: a 10-year cohort controlled study. Spine J. 2012.12(10):881-90. doi: https://dx.doi.org/10.1016/j.spinee.2012.09.040 | 1059 | Off label use of rhBMP |
| 54 | Comparison of clinical and radiologic outcomes between oblique lumbar interbody fusion and minimal invasive transforminal lumbar interbody fusion in spondylolisthesis : a randomized controlled trial. Identifier: TCTR20200830001. In: Thai Clinical Trials Registry (TCTR) [internet]. Bangkok: Medical Research Foundation Thailand: 2020. Available from https://www.thaiclinicaltrials.org/show/TCTR20200830001. | 4859 | Ongoing trial |
| 55 | Cooper GS, Kou TD. Risk of cancer after lumbar fusion surgery with recombinant human bone morphogenic protein-2 (rh-BMP-2). Spine. 2013.38(21):1862-8. doi: https://dx.doi.org/10.1097/BRS.0b013e3182a3d3b4 | 906 | Surgical route not reported |
| 56 | Cooper GS, Kou TD. Risk of cancer following lumbar fusion surgery with recombinant human bone morphogenic protein-2 (rhBMP-2): An analysis using a commercially insured patient population. Int J Spine Surg. 2018.12(2):260-68. doi: https://dx.doi.org/10.14444/50323 | 615 | Ineligible patient population |
| 57 | Cowles RA TP, Sweeney JF, Graziano GP. Efcacy of the laparoscopic approach for anterior lumbar spinal fusion. Surgery. 2000.128(4):589–96. | 4954 | Ineligible outcomes |
| 58 | Cuellar JM, Rasouli A, Lanman TH, Kanim L, Delamarter R. Single and multilevel lumbar total disc replacement adjacent to L5-S1 ALIF (lumbar hybrid): 6 years of follow-up. Int J Spine Surg. 2021.15(5):971-77. doi: https://dx.doi.org/10.14444/8127 | 3056 | Off label use of rhBMP |
| 59 | Cumming D, Song F, Taylor RS, Zahra M, Williams A, Eggington S. Cost-effectiveness of 4 mg dibotermin alfa/absorbable collagen sponge versus iliac crest bone graft for lumbar degenerative disc disease in the United Kingdom. J Med Econ. 2022.25(1):59-65. doi: https://dx.doi.org/10.1080/13696998.2021.2017200 | 213 | ALIF/OLIF not reported separately |
| 60 | Dagostino PR, Whitmore RG, Smith GA, Maltenfort MG, Ratliff JK. Impact of bone morphogenetic proteins on frequency of revision surgery, use of autograft bone, and total hospital charges in surgery for lumbar degenerative disease: review of the Nationwide Inpatient Sample from 2002 to 2008. Spine J. 2014.14(1):20-30. doi: https://dx.doi.org/10.1016/j.spinee.2012.10.035 | 879 | Ineligible route of administration |
| 61 | DePuy Spine. CHARITÉ™ artificial disc compared to anterior interbody fusion for treatment of degenerative disc disease. Identifier: NCT00215306. In: ClinicalTrials.gov [internet]. Bethesda: US National Library of Medicine: 2000. Available from https://ClinicalTrials.gov/show/NCT00215306. | 4171 | Ineligible intervention |
| 62 | DePuy Spine. CHARITE™ vs. ALIF 5-year follow-up. Identifier: NCT00215332. In: ClinicalTrials.gov [internet]. Bethesda: US National Library of Medicine: 2005. Available from https://ClinicalTrials.gov/show/NCT00215332. | 4151 | Trial record >10 years, no results posted |
| 63 | DePuy Spine. Titanium surgical mesh and MOSS-Miami screws for lumbar fusion. Identifier: NCT00215319. In: ClinicalTrials.gov [internet]. Bethesda: US National Library of Medicine: 2000. Available from https://ClinicalTrials.gov/show/NCT00215319. | 4013 | Trial record >10 years, no results posted |
| 64 | Dettori JR, Chapman JR, DeVine JG, McGuire RA, Junge MR, Norvell DC. Longer follow-up continues to reveal no increased risk of cancer with the use of recombinant human bone morphogenetic protein in spine fusion. Spine J. 2019.19(10):1640-47. doi: https://dx.doi.org/10.1016/j.spinee.2019.05.005 | 411 | Ineligible patient population |
| 65 | Dettori JR, Chapman JR, DeVine JG, McGuire RA, Norvell DC, Weiss NS. The risk of cancer with the use of recombinant human bone morphogenetic protein in spine fusion. Spine. 2016.41(16):1317-24. doi: https://dx.doi.org/10.1097/BRS.0000000000001671 | 683 | Ineligible patient population |
| 66 | D'Oro A, Buser Z, Brodke DS, Park J-B, Yoon ST, Youssef JA, et al. Trends and costs of external electrical bone stimulators and grafting materials in anterior lumbar interbody fusion. Asian Spine J. 2018.12(6):973-80. doi: https://dx.doi.org/10.31616/asj.2018.12.6.973 | 610 | ALIF/OLIF not reported separately |
| 67 | Du X, Ou Y-S, Zhu Y, Luo W, Jiang G-Y, Jiang D-M. Oblique lateral interbody fusion combined percutaneous pedicle screw fixation in the surgical treatment of single-segment lumbar tuberculosis: A single-center retrospective comparative study. Int J Surg. 2020.83:39-46. doi: https://dx.doi.org/10.1016/j.ijsu.2020.09.012 | 320 | Ineligible patient population |
| 68 | Edgard-Rosa G, Geneste G, Negre G, Marnay T. Midline anterior approach from the right side to the lumbar spine for interbody fusion and total disc replacement: A new mobilization technique of the vena cava. Spine. 2012.37(9):E562-E69. doi: https://dx.doi.org/10.1097/BRS.0b013e31823a0a87 | 2695 | Ineligible patient population |
| 69 | El Masry MA, Badawy WS, Rajendran P, Chan D. Combined anterior interbody fusion and posterior pedicle screw fixation in patients with degenerative lumbar disc disease. Int Orthop. 2004.28(5):294-7. | 1631 | Ineligible patient population |
| 70 | Elhessy AH, Eltayeby HH, Kane SC, Garonzik IM, Conway JE, Conway JD. Fusion revision surgery with reamer-irrigator-aspirator to harvest autograft after spinal pseudarthrosis. Cureus. 2022.14(7):e27503. doi: https://dx.doi.org/10.7759/cureus.27503 | 67 | <10 patients |
| 71 | el-Masry MA, Katsochis A, Badawy WS, el-Hawary YK. Anterior lumbar interbody fusion using a hybrid graft. Acta Orthop Belg. 2004.70(4):332-6. | 1622 | Ineligible patient population |
| 72 | Epstein NE, Schwall GS. Costs and frequency of "off-label" use of INFUSE for spinal fusions at one institution in 2010. Surg Neurol Int. 2011.2:115. doi: https://dx.doi.org/10.4103/2152-7806.83929 | 1125 | Ineligible patient population |
| 73 | Escobar E, Transfeldt E, Garvey T, Ogilvie J, Graber J, Schultz L. Video-assisted versus open anterior lumbar spine fusion surgery: a comparison of four techniques and complications in 135 patients. Spine. 2003.28(7):729-32. | 1724 | Mixed interventions |
| 74 | Fayyazi AH, Buckley R, Lorio M, Yuan P, Devine JG, Smith KK. Evaluation of radiographic and patient outcomes following lumbar spine fusion using demineralized bone matrix (DBM) mixed with autograft. Spine J. 2010.(10 :(9 Suppl 1)):27s‐28s. | 3417 | conference abstract |
| 75 | Feeley A, Feeley I, Clesham K, Butler J. Is there a variance in complication types associated with ALIF approaches? A systematic review. Acta Neurochir. 2021.163(11):2991-3004. doi: https://dx.doi.org/10.1007/s00701-021-05000-0 | 244 | SR to check |
| 76 | Feng J-T, Yang X-G, Wang F, He X, Hu Y-C. Efficacy and safety of bone substitutes in lumbar spinal fusion: a systematic review and network meta-analysis of randomized controlled trials. Eur Spine J. 2020.29(6):1261-76. doi: https://dx.doi.org/10.1007/s00586-019-06257-x | 379 | SR to check |
| 77 | Flouzat-Lachaniette C-H, Ghazanfari A, Bouthors C, Poignard A, Hernigou P, Allain J. Bone union rate with recombinant human bone morphogenic protein-2 versus autologous iliac bone in PEEK cages for anterior lumbar interbody fusion. Int Orthop. 2014.38(9):2001-7. doi: https://dx.doi.org/10.1007/s00264-014-2301-6 | 844 | Ineligible patient population |
| 78 | Flouzat-Lachaniette CH, Ratte L, Poignard A, Auregan JC, Queinnec S, Hernigou P, Allain J. Minimally invasive anterior lumbar interbody fusion for adult degenerative scoliosis with 1 or 2 dislocated levels. J Neurosurg Pediatr: Spine. 2015.23(6):739-46. doi: https://dx.doi.org/10.3171/2015.3.SPINE14772 | 2702 | Single level/multiple level not reported separately |
| 79 | Formica M, Vallerga D, Zanirato A, Cavagnaro L, Basso M, Divano S, et al. Fusion rate and influence of surgery-related factors in lumbar interbody arthrodesis for degenerative spine diseases: a meta-analysis and systematic review. Musculoskelet Surg. 2020.104(1):1-15. doi: https://dx.doi.org/10.1007/s12306-019-00634-x | 377 | SR to check |
| 80 | Frantzides CT et al. L5-S1 laparoscopic anterior interbody fusion. JSLS. 2006.10(4):488–92. | 4953 | Ineligible intervention |
| 81 | Fujibayashi S, Kawakami N, Asazuma T, Ito M, Mizutani J, Nagashima H, et al. Complications associated with lateral interbody fusion: Nationwide survey of 2998 cases during the first 2 years of its use in Japan. Spine. 2017.42(19):1478-84. doi: https://dx.doi.org/10.1097/BRS.0000000000002139 | 2262 | Mixed interventions |
| 82 | Fussi J, Pumberger M, Zahn RK, Putzier M. Lumbar total disc replacement versus anterior interbody fusion: a prospective-randomized control study-average followup of 10 years. Eur Spine J. 2016.S320. doi: 10.1007/s00586-016-4722-y | 3431 | conference abstract |
| 83 | Geale K, Alvarez M, Polyzoi M, Malaga X, Pineda C, Hernandez C. Budget impact analysis of demineralized bone matrix in combination with autograft in lumbar spinal fusion procedures for the treatment of lumbar degenerative disc disease in Spain. J Med Econ. 2018.21(10):977-82. doi: https://dx.doi.org/10.1080/13696998.2018.1489256 | 466 | Surgical route not reported |
| 84 | Geerdes BP, Geukers CW, van Erp WF. Laparoscopic spinal fusion of L4–L5 and L5–S1. Surg Endosc. 2001.15(11):1308–12. | 4956 | Ineligible intervention |
| 85 | Geisler FH, Blumenthal SL, Guyer RD, McAfee PC, Regan JJ, Johnson JP, Mullin B. Neurological complications of lumbar artificial disc replacement and comparison of clinical results with those related to lumbar arthrodesis in the literature: results of a multicenter, prospective, randomized investigational device exemption study of Charite intervertebral disc. Invited submission from the Joint Section Meeting on Disorders of the Spine and Peripheral Nerves, March 2004. J Neurosurg Spine. 2004.1(2):143-54. | 1629 | Ineligible intervention |
| 86 | Geisler FH, Guyer RD, Blumenthal SL, McAfee PC, Cappuccino A, Bitan F, Regan JJ. Effect of previous surgery on clinical outcome following 1-level lumbar arthroplasty. J Neurosurg Spine. 2008.8(2):108-14. doi: https://dx.doi.org/10.3171/SPI/2008/8/2/108 | 1430 | Ineligible intervention |
| 87 | Geistlich Pharma AG. Evidence for fusion in spine with Orthoss®. Identifier: DRKS00016578. In: German Clinical Trials Register [internet]. Freiburg: Institute for Medical Biometry and Statistics - University of Freiburg: 2019. Available from http://www.drks.de/DRKS00016578. | 4869 | Ongoing trial |
| 88 | Geistlich Pharma AG. Evidence for fusion in spine with Orthoss®. Identifier: NCT03853356. In: ClinicalTrials.gov [internet]. Bethesda: US National Library of Medicine: 2019. Available from https://ClinicalTrials.gov/show/NCT03853356. | 3944 | Ineligible patient population |
| 89 | Genera Research Ltd. Blinded study on safety, tolerability and lumbar fusion efficacy of a single dose of Osteogrow (which comprises a protein called BMP6 mixed with the patients own blood to form a clot) in adult patients treated by spinal fusion (posterolateral lumbar interbody fusion, PLIF) for degenerative disc disease. Identifier: EUCTR2017-000860-14-AT. In: EU Clinical Trials Register [internet]. Amsterdam: European Medicines Agency: 2018. Available from https://www.clinicaltrialsregister.eu/ctr-search/search?query=eudract_number:2017-000860-14. | 4826 | Ineligible intervention |
| 90 | Girardi FP, Cammisa FP, Jr. The effect of bone graft extenders to enhance the performance of iliac crest bone grafts in instrumented lumbar spine fusion. Orthopedics. 2003.26(5 Suppl):s545-8. | 1719 | Surgical route not reported |
| 91 | Gonzalez-Tartiere P, de Frutos AG, Ubierna MT, Arco AD, Salo G, Matamalas A, et al. P136. Randomized, prospective clinical trial to evaluate efficacy and safety in lumbar fusion surgery of implantation of autologous bone marrow mesenchymal cells expanded ex vivo and combined with allogeneic bone tissue, compared with autologous iliac crest graft; part II: clinical findings. Spine J. 2019.(9):S220‐s21. doi: 10.1016/j.spinee.2019.05.561 | 3597 | conference abstract |
| 92 | Gornet MF, Dryer RF, Peloza JH, Schranck FW. Lumbar disc arthroplasty vs. anterior lumbar interbody fusion: five-year outcomes for patients in the Maverickdegree disc IDE study. Spine J. 2010.(10:(9 Suppl. 1)):64s. | 3446 | conference abstract |
| 93 | Gruskay JA, Basques BA, Bohl DD, Webb ML, Grauer JN. Short-term adverse events, length of stay, and readmission after iliac crest bone graft for spinal fusion. Spine. 2014.39(20):1718-24. doi: https://dx.doi.org/10.1097/BRS.0000000000000476 | 3053 | ALIF/OLIF not reported separately |
| 94 | Guppy KH, Chan PH, Prentice HA, Norheim EP, Harris JE, Brara HS. Does the use of preoperative bisphosphonates in patients with osteopenia and osteoporosis affect lumbar fusion rates? Analysis from a national spine registry. Neurosurg Focus. 2020.49(2):E12. doi: https://dx.doi.org/10.3171/2020.5.FOCUS20262 | 328 | Ineligible patient population |
| 95 | Guyer RD, McAfee PC, Banco RJ, Bitan FD, Cappuccino A, Geisler FH, et al. Prospective, randomized, multicenter Food and Drug Administration investigational device exemption study of lumbar total disc replacement with the CHARITE artificial disc versus lumbar fusion: five-year follow-up. Spine J. 2009.9(5):374-86. doi: https://dx.doi.org/10.1016/j.spinee.2008.08.007 | 1369 | Ineligible intervention |
| 96 | Guzman JZ, Merrill RK, Kim JS, Overley SC, Dowdell JE, Somani S, et al. Bone morphogenetic protein use in spine surgery in the United States: how have we responded to the warnings? Spine J. 2017.17(9):1247-54. doi: https://dx.doi.org/10.1016/j.spinee.2017.04.030 | 541 | Ineligible outcomes |
| 97 | Hagel A, Zeh A, Hein W, Held A, Wohlrab D. Comparison of anterior lumbar fusion rates after circumferential fusion using beta-tricalcium phosphate (Cerasorb) versus autologous iliac crest spongiosa. Zeitschrift fur Orthopadie und Unfallchirurgie. 2007.(4):488‐92. doi: 10.1055/s-2007-965252 | 3428 | Non-English language |
| 98 | Han P-F, Chen T-Y, Zhang Z-L, Li X-D, Li P-C, Wei L, et al. rhBMP in lumber fusion for lumbar spondylolisthesis: A systematic review and meta-analysis. Chinese Journal of Traumatology. 2019.22(1):51-58. doi: https://dx.doi.org/10.1016/j.cjtee.2018.10.003 | 430 | SR to check |
| 99 | Haukeland University Hospital. Degenerative spondylolisthesis; micro-decompression alone vs decompression plus instrumented fusion on long term follow up. Identifier: NCT03469791. In: ClinicalTrials.gov [internet]. Bethesda: US National Library of Medicine: 2007. Available from https://ClinicalTrials.gov/show/NCT03469791. | 4216 | Ineligible intervention |
| 100 | He W, He D, Tian W, Schaller B. Evaluation of lumbar fusion using the anterior to psoas approach for the treatment of L5/S1 spondylolisthesis. Med. 2020.99(23):e20014. doi: https://dx.doi.org/10.1097/MD.0000000000020014 | 2432 | <10 patients |
| 101 | Hindoyan K, Tilan J, Buser Z, Cohen JR, Brodke DS, Youssef JA, et al. A retrospective analysis of complications associated with bone morphogenetic protein 2 in anterior lumbar interbody fusion. Global Spine J. 2017.7(2):148-53. doi: https://dx.doi.org/10.1177/2192568217694010 | 650 | DDD not reported separately |
| 102 | Hirasawa M, Mure H, Toi H, Nagahiro S. Surgical results of lumbar interbody fusion using calcium phosphate cement. Neurol Med Chir. 2014.54(9):722-6. | 805 | Ineligible intervention |
| 103 | Hu Z, He D, Gao J, Zeng Z, Jiang C, Ni W, et al. The influence of endplate morphology on cage subsidence in patients with stand-alone oblique lateral lumbar interbody fusion (OLIF). Global Spine J. 2023.13(1):97-103. doi: https://dx.doi.org/10.1177/2192568221992098 | 3216 | DDD not reported separately |
| 104 | Huang C-Y, Yeh K-T, Yu T-C, Lee R-P, Chen I-H, Peng C-H, et al. Surgical results of a one-stage combined anterior lumbosacral fusion and posterior percutaneous pedicle screw fixation. TCMJ. 2018.30(1):20-23. doi: https://dx.doi.org/10.4103/tcmj.tcmj_186_17 | 626 | Ineligible patient population |
| 105 | Invibio Ltd. A clinical study to evaluate safety and performance of PEEK-OPTIMA™ HA enhanced interbody cages in the lumbar spine. Identifier: NCT03928041. In: ClinicalTrials.gov [internet]. Bethesda: US National Library of Medicine: 2019. Available from https://ClinicalTrials.gov/show/NCT03928041. | 4096 | Ongoing trial |
| 106 | Invibio Ltd. A clinical study to evaluate safety and performance of PEEK-OPTIMA™ HA enhanced. Identifier: NCT04416321. In: ClinicalTrials.gov [internet]. Bethesda: US National Library of Medicine: 2021. Available from https://ClinicalTrials.gov/show/NCT04416321. | 4183 | Ongoing trial |
| 107 | Ishihara H, Osada R, Kanamori M, Kawaguchi Y, Ohmori K, Kimura T, et al. Minimum 10-year follow-up study of anterior lumbar interbody fusion for isthmic spondylolisthesis. J Spinal Disord. 2001.14(2):91-99. doi: https://dx.doi.org/10.1097/00002517-200104000-00001 | 2735 | Ineligible patient population |
| 108 | ISTO Technologies Inc. Prospective study of safety and efficacy of InQu® bone graft extender in lumbar interbody fusion surgery. Identifier: NCT02400762. In: ClinicalTrials.gov [internet]. Bethesda: US National Library of Medicine: 2013. Available from https://ClinicalTrials.gov/show/NCT02400762. | 3863 | Ineligible patient population |
| 109 | Jackle K, Saul D, Oberthur S, Roch PJ, Sehmisch S, Lehmann W, Weiser L. Long-term quality of life after fusion of the ventral thoracic and lumbar spine. J Neurol Surg A Cent Eur Neurosurg. 2022.83(2):153-60. doi: https://dx.doi.org/10.1055/s-0041-1736324 | 214 | Ineligible patient population |
| 110 | Jaeger A, Giber D, Bastard C, Thiebaut B, Roubineau F, Flouzat Lachaniette CH, Dubory A. Risk factors of instrumentation failure and pseudarthrosis after stand-alone L5-S1 anterior lumbar interbody fusion: a retrospective cohort study. J Neurosurg Spine. 2019.31(3):338-46. doi: https://dx.doi.org/10.3171/2019.3.SPINE181476 | 410 | Ineligible patient population |
| 111 | Jeon CH, Lee HD, Chung NS. Does mechanical bowel preparation ameliorate surgical performance in anterior lumbar interbody fusion? Global Spine J. 2019.9(7):692-96. doi: https://dx.doi.org/10.1177/2192568218825249 | 2346 | Ineligible patient population |
| 112 | Johann Wolfgang Goethe University Hospital. Evaluation of fusion after ALIF with an osteoinductive material or with homologus bone. Identifier: NCT03331159. In: ClinicalTrials.gov [internet]. Bethesda: US National Library of Medicine: 2012. Available from https://ClinicalTrials.gov/show/NCT03331159. | 4057 | Trial record >10 years, no results posted |
| 113 | Johnson RG. Bone marrow concentrate with allograft equivalent to autograft in lumbar fusions. Spine. 2014.39(9):695-700. doi: https://dx.doi.org/10.1097/BRS.0000000000000254 | 851 | Ineligible patient population |
| 114 | Kansas Joint and Spine Institute. Outcomes of degenerative disc disease patients treated with an anterior-only fusion using InQu bone graft. Identifier: NCT01746212. In: ClinicalTrials.gov [internet]. Bethesda: US National Library of Medicine: 2012. Available from https://ClinicalTrials.gov/show/NCT01746212. | 3738 | Trial record >10 years, no results posted |
| 115 | Kapustka B et al. Anterior lumbar interbody fusion (ALIF): biometrical results and own experiences. Neurosurg Rev. 2020.43(2):687–93. | 4950 | Ineligible intervention |
| 116 | Kasis AG, Jensen C, Dharmadhikari R, Emmerson BR, Mawdsley M. Novel bone grafting technique in stand-alone ALIF procedure combining allograft and autograft ('Northumbria Technique')-Fusion rate and functional outcomes in 100 consecutive patients. Eur Spine J. 2021.30(5):1296-302. doi: https://dx.doi.org/10.1007/s00586-021-06758-8 | 289 | Ineligible intervention |
| 117 | Kayanja M, Orr RD. Incidence and outcome of graft resorption in anterior lumbar interbody fusion: using femoral ring allografts and recombinant human bone morphogenetic protein-2. Spine. 2014.39(5):374-80. doi: https://dx.doi.org/10.1097/BRS.0000000000000145 | 861 | Single level/multiple level not reported separately |
| 118 | Kerr EJ, 3rd, Jawahar A, Wooten T, Kay S, Cavanaugh DA, Nunley PD. The use of osteo-conductive stem-cells allograft in lumbar interbody fusion procedures: an alternative to recombinant human bone morphogenetic protein. J Surg Orthop Adv. 2011.20(3):193-7. | 1153 | Single level/multiple level not reported separately |
| 119 | Khalid SI, Nunna RS, Shanker RM, Thomson KB, Parola R, Adogwa O, Mehta AI. Bone morphogenetic protein in anterior lumbar interbody fusions: a propensity-matched Medicare outcome analysis. Int J Spine Surg. 2022.14:14. doi: https://dx.doi.org/10.14444/8301 | 9 | Ineligible patient population |
| 120 | Khanna G, Lewonowski K, Wood KB. Initial results of anterior interbody fusion achieved with a less invasive bone harvesting technique. Spine. 2006.31(1):111-4. | 1550 | Single level/multiple level not reported separately |
| 121 | Kiely PD, Mount LE, Du JY, Nguyen JT, Weitzman G, Memstoudis S, et al. The incidence and risk factors for post-operative ileus after spinal fusion surgery: a multivariate analysis. Int Orthop. 2016.40(6):1067-74. doi: https://dx.doi.org/10.1007/s00264-016-3148-9 | 3213 | Ineligible patient population |
| 122 | Kim DH, Lee N, Shin DA, Yi S, Kim KN, Ha Y. Matched comparison of fusion rates between hydroxyapatite demineralized bone matrix and autograft in lumbar interbody fusion. J Korean Neurosurg Soc. 2016.59(4):363-7. doi: https://dx.doi.org/10.3340/jkns.2016.59.4.363 | 991 | Ineligible patient population |
| 123 | Kim JS, Seong JH. Endoscope-assisted oblique lumbar interbody fusion for the treatment of cauda equina syndrome: a technical note. Eur Spine J. 2017.26(2):397-403. doi: https://dx.doi.org/10.1007/s00586-016-4902-9 | 2404 | Ineligible study design |
| 124 | Koehler S, Held C, Stetter C, Westermaier T. Alloplastic or autologous? Bone chips versus PEEK cage for lumbar interbody fusion in degenerative spondylolisthesis. J Neurol Surg A Cent Eur Neurosurg. 2021.82(6):562-67. doi: https://dx.doi.org/10.1055/s-0040-1718770 | 275 | Ineligible patient population |
| 125 | Konig MA, Ebrahimi FV, Nitulescu A, Behrbalk E, Boszczyk BM. Early results of stand-alone anterior lumbar interbody fusion in iatrogenic spondylolisthesis patients. Eur Spine J. 2013.22(12):2876-83. doi: https://dx.doi.org/10.1007/s00586-013-2970-7 | 890 | <10 patients |
| 126 | Kotheeranurak V, Lin GX, Mahatthanatrakul A, Kim JS. Endoscope-assisted anterior lumbar interbody fusion with computed tomography-guided, image-navigated unilateral cortical bone trajectory screw fixation in managing adjacent segment disease in L5/S1: Technical note. World Neurosurg. 2019.122:469-73. doi: https://dx.doi.org/10.1016/j.wneu.2018.10.029 | 2403 | Ineligible study design |
| 127 | Kuang L, Wang B, Lu G. Transforaminal lumbar interbody fusion versus mini-open anterior lumbar interbody fusion with oblique self-anchored stand-alone cages for the treatment of lumbar disc herniation: A retrospective study with 2-year follow-up. Spine. 2017.42(21):E1259-E65. doi: 10.1097/BRS.0000000000002145 | 4969 | Ineligible intervention |
| 128 | Kumar N, Wild A, Webb JK, Aebi M. Hybrid computer-guided and minimally open surgery: anterior lumbar interbody fusion and translaminar screw fixation. Eur Spine J. 2000.9 Suppl 1:S71-7. | 1872 | Ineligible study design |
| 129 | Lad SP, Bagley JH, Karikari IO, Babu R, Ugiliweneza B, Kong M, et al. Cancer after spinal fusion: the role of bone morphogenetic protein. Neurosurgery. 2013.73(3):440-9. doi: https://dx.doi.org/10.1227/NEU.0000000000000018 | 915 | ALIF/OLIF not reported separately |
| 130 | Lad SP, Nathan JK, Boakye M. Trends in the use of bone morphogenetic protein as a substitute to autologous iliac crest bone grafting for spinal fusion procedures in the United States. Spine. 2011.36(4):E274-81. doi: https://dx.doi.org/10.1097/BRS.0b013e3182055a6b | 1202 | ALIF/OLIF not reported separately |
| 131 | Lammli J, Whitaker MC, Moskowitz A, Duong J, Dong F, Felts L, et al. Stand-alone anterior lumbar interbody fusion for degenerative disc disease of the lumbar spine: results with a 2-year follow-up. Spine. 2014.39(15):E894-901. doi: https://dx.doi.org/10.1097/BRS.0000000000000393 | 836 | Off label use of rhBMP |
| 132 | Lang SAJ, Bohn T, Barleben L, Pumberger M, Roll S, Buttner-Janz K. Advanced meta-analyses comparing the three surgical techniques total disc replacement, anterior stand-alone fusion and circumferential fusion regarding pain, function and complications up to 3 years to treat lumbar degenerative disc disease. Eur Spine J. 2021.30(12):3688-701. doi: https://dx.doi.org/10.1007/s00586-021-06784-6 | 1974 | SR to check |
| 133 | Lanman TH, Hopkins TJ. Lumbar interbody fusion after treatment with recombinant human bone morphogenetic protein-2 added to poly(L-lactide-co-D,L-lactide) bioresorbable implants. Neurosurg Focus. 2004.16(3):E9. | 2654 | Ineligible patient population |
| 134 | Lao L, Cohen JR, Buser Z, Brodke DS, Yoon ST, Youssef JA, et al. Trends analysis of rhBMP2 utilization in single-level anterior lumbar interbody fusion in the United States. Global Spine J. 2018.8(2):137-41. doi: https://dx.doi.org/10.1177/2192568217701119 | 625 | Ineligible outcomes |
| 135 | Lastfogel JF, Altstadt TJ, Rodgers RB, Horn EM. Sacral fractures following stand-alone L5-S1 anterior lumbar interbody fusion for isthmic spondylolisthesis. J Neurosurg Pediatr: Spine. 2010.13(2):288-93. doi: https://dx.doi.org/10.3171/2010.3.SPINE09366 | 3030 | <10 patients |
| 136 | Latzman JM, Kong L, Liu C, Samadani U. Administration of human recombinant bone morphogenetic protein-2 for spine fusion may be associated with transient postoperative renal insufficiency. Spine. 2010.35(7):E231-7. doi: https://dx.doi.org/10.1097/BRS.0b013e3181c71447 | 1269 | Ineligible patient population |
| 137 | Lavelle W, McLain RF, Rufo-Smith C, Gurd DP. Prospective randomized controlled trial of the stabilis Stand Alone Cage (SAC) versus Bagby and Kuslich (BAK) implants for anterior lumbar interbody fusion. Int J Spine Surg. 2014.8:8. doi: https://dx.doi.org/10.14444/1008 | 2922 | Single level/multiple level not reported separately |
| 138 | LDR Spine USA. Retrospective/prospective data collection on the LDR ROIA interbody fusion device with VerteBRIDGE plating. Identifier: NCT02104141. In: ClinicalTrials.gov [internet]. Bethesda: US National Library of Medicine: 2013. Available from https://ClinicalTrials.gov/show/NCT02104141. | 4172 | Trial record >10 years, no results posted |
| 139 | Lechner R, Putzer D, Liebensteiner M, Bach C, Thaler M. Fusion rate and clinical outcome in anterior lumbar interbody fusion with beta-tricalcium phosphate and bone marrow aspirate as a bone graft substitute. A prospective clinical study in fifty patients. Int Orthop. 2017.41(2):333-39. doi: https://dx.doi.org/10.1007/s00264-016-3297-x | 570 | Single level/multiple level not reported separately |
| 140 | Lee DD, Kim JY. A comparison of radiographic and clinical outcomes of anterior lumbar interbody fusion performed with either a cellular bone allograft containing multipotent adult progenitor cells or recombinant human bone morphogenetic protein-2. Journal of Orthopaedic Surgery. 2017.12(1):126. doi: https://dx.doi.org/10.1186/s13018-017-0618-8 | 521 | Off label use of rhBMP |
| 141 | Lee DH, Lee DG, Hwang JS, Jang JW, Maeng DH, Park CK. Clinical and radiological results of indirect decompression after anterior lumbar interbody fusion in central spinal canal stenosis. J Neurosurg Pediatr: Spine. 2021.34(4):564-72. doi: https://dx.doi.org/10.3171/2020.7.SPINE191335 | 2154 | Single level/multiple level not reported separately |
| 142 | Lefevre E, d'Astorg H, Fiere V, Gauthe R, Vieira TD, Ould Slimane M, Szadkowski M. Treatment of one-level degenerative lumbar spondylolisthesis with severe stenosis by oblique lateral interbody fusion: Clinical and radiological results after a minimal 1-year follow-up. Interdiscip Neurosurg. 2021.26:101321. doi: https://dx.doi.org/10.1016/j.inat.2021.101321 | 3315 | Ineligible patient population |
| 143 | Lekovic GP, Han PP, Kenny KJ, Dickman CA. Bone dowels in anterior lumbar interbody fusion. J Spinal Disord Tech. 2007.20(5):374-9. | 1472 | Single level/multiple level not reported separately |
| 144 | Li J, Dumonski ML, Liu Q, Lipman A, Hong J, Yang N, et al. A multicenter study to evaluate the safety and efficacy of a stand-alone anterior carbon I/F Cage for anterior lumbar interbody fusion: two-year results from a Food and Drug Administration investigational device exemption clinical trial. Spine. 2010.35(26):E1564-70. doi: 10.1097/BRS.0b013e3181ef5c14 | 4970 | Ineligible intervention |
| 145 | Li J-C, Xie T-H, Zhang Z, Song Z-T, Song Y-M, Zeng J-C. The mismatch between bony endplates and grafted bone increases screw loosening risk for OLIF patients with ALSR fixation biomechanically. Front Bioeng Biotechnol. 2022.10:862951. doi: https://dx.doi.org/10.3389/fbioe.2022.862951 | 75 | Ineligible outcomes |
| 146 | Li Y, Yu Y, Hou T-Y, Zhang Z-H, Xing J-C, Lu H-W, et al. Efficacy of Biocage in treating single-segment lumbar degenerative disease in patients with high risk of non-fusion: a prospective controlled study with at least 2 years' follow-up. J Int Med Res. 2020.48(9):300060520945500. doi: https://dx.doi.org/10.1177/0300060520945500 | 317 | Ineligible intervention |
| 147 | Li Y-D, Chi J-E, Chiu P-Y, Kao F-C, Lai P-L, Tsai T-T. The comparison between anterior and posterior approaches for removal of infected lumbar interbody cages and a proposal regarding the use of endoscope-assisted technique. Journal of Orthopaedic Surgery. 2021.16(1):386. doi: https://dx.doi.org/10.1186/s13018-021-02535-x | 262 | Ineligible outcomes |
| 148 | Li Z-Z, Wang J-C, Cao Z, Zhao H-L, Lewandrowski K-U, Yeung A. Full-endoscopic oblique lateral lumbar interbody fusion: A technical note with 1-year follow-up. Int J Spine Surg. 2021.15(3):504-13. doi: https://dx.doi.org/10.14444/8072 | 113 | Off label use of rhBMP |
| 149 | Limthongkul W, Chantharakomen R, Tanasansomboon T, Yingsakmongkol W, Yoong-Leong Oh J, Kotheeranurak V, Singhatanadgige W. Comparison of Unremoved Intervertebral Disc Location Between 2 Lateral Lumbar Interbody Fusion (LLIF) Techniques. World Neurosurg. 2022.160((Limthongkul, Yingsakmongkol, Singhatanadgige) Department of Orthopedics, Faculty of Medicine, Chulalongkorn University, Bangkok, Thailand):e322-e27. doi: https://dx.doi.org/10.1016/j.wneu.2022.01.011 | 2254 | Single level/multiple level not reported separately |
| 150 | Lindenhofgruppe AG. Comparison of standalone anterior lumbar interbody fusion L5/S1 performed with either rrhBMP-2 or ViviGen®. Identifier: NCT05238740. In: ClinicalTrials.gov [internet]. Bethesda: US National Library of Medicine: 2022. Available from https://ClinicalTrials.gov/show/NCT05238740. | 3735 | Ongoing trial |
| 151 | Litrico S, Langlais T, Pennes F, Gennari A, Paquis P. Lumbar interbody fusion with utilization of recombinant human bone morphogenetic protein: a retrospective real-life study about 277 patients. Neurosurg Rev. 2018.41(1):189-96. doi: https://dx.doi.org/10.1007/s10143-017-0834-z | 512 | Off label use of rhBMP |
| 152 | Liu JC, Ondra SL, Angelos P, Ganju A, Landers ML. Is laparoscopic anterior lumbar interbody fusion a useful minimally invasive procedure? Neurosurgery. 2002.51(5 Suppl):S155-8. | 4971 | Ineligible intervention |
| 153 | Liu S, Wang Y, Liang Z, Zhou M, Chen C. Comparative clinical effectiveness and safety of bone morphogenetic protein versus autologous iliac crest bone graft in lumbar fusion: A meta-analysis and systematic review. Spine. 2020.45(12):E729-E41. doi: https://dx.doi.org/10.1097/BRS.0000000000003372 | 375 | SR to check |
| 154 | Liu XJ, Zhu QS, Sun HF, Song XJ, Wang CL, Wu YT, Ma YH. The clinical efficacy of hydroxyapatite and its composites in spinal reconstruction: a meta-analysis. Eur Rev Med Pharmacol Sci. 2022.26(13):4614-24. doi: https://dx.doi.org/10.26355/eurrev_202207_29183 | 172 | SR to check |
| 155 | Lloyd AP. Counting the cost of failed spinal fusion for relief of low back pain: does primary fusion with bone morphogenetic protein make economic sense from a primary payer perspective? Clin Spine Surg. 2017.30(6):E720-E24. doi: https://dx.doi.org/10.1097/BSD.0000000000000273 | 534 | Single level/multiple level not reported separately |
| 156 | Long-term effects of inorganic inducing factor scaffolds versus autologous bone in the treatment of lumbar interbody fusion: a non-randomized, controlled 2-year follow-up trial. Identifier: ChiCTR1900021333. In: EU Clinical Trials Register [internet]. Amsterdam: European Medicines Agency: 2019. Available from http://www.chictr.org.cn/showproj.aspx?proj=36019. | 4868 | Ineligible intervention |
| 157 | Louie PK, Hassanzadeh H, Singh K. Epidemiologic trends in the utilization, demographics, and cost of bone morphogenetic protein in spinal fusions. Curr Rev Musculoskelet Med. 2014.7(3):177-81. doi: https://dx.doi.org/10.1007/s12178-014-9222-2 | 1041 | Ineligible study design |
| 158 | Lu C, Gai JY, Lu GH. Clinical outcomes of posterior versus anterior lumbar interbody fusion for high level lumbar intervertebral disc herniation. CRTER. 2009.(35):6861‐66. | 3480 | Non-English language |
| 159 | Lubelski D, Abdullah KG, Nowacki AS, Alvin MD, Steinmetz MP, Chakka S, et al. Urological complications following use of recombinant human bone morphogenetic protein-2 in anterior lumbar interbody fusion: presented at the 2012 Joint Spine Section Meeting: clinical article. J Neurosurg Spine. 2013.18(2):126-31. doi: https://dx.doi.org/10.3171/2012.11.SPINE12389 | 954 | Single level/multiple level not reported separately |
| 160 | Lubelski D, Abdullah KG, Steinmetz MP, Alvin MD, Nowacki AS, Chakka S, et al. Adverse events with the use of rhBMP-2 in thoracolumbar and lumbar spine fusions: A 9-year institutional analysis. J Spinal Disord Tech. 2015.28(5):E277-83. doi: https://dx.doi.org/10.1097/BSD.0b013e318287f2e2 | 785 | Single level/multiple level not reported separately |
| 161 | Lubelski D, Alvin MD, Torre-Healy A, Abdullah KG, Nowacki AS, Whitmore RG, et al. Quality-of-life outcomes following thoracolumbar and lumbar fusion with and without the use of recombinant human bone morphogenetic protein-2: Does recombinant human bone morphogenetic protein-2 make a difference? Glob Spine J. 2014.4(4):245-54. doi: https://dx.doi.org/10.1055/s-0034-1394123 | 1034 | ALIF/OLIF not reported separately |
| 162 | Lv Z-T, Xu Y, Cao B, Dai J, Zhang S-Y, Huang J-M, et al. Titanium-coated PEEK Versus Uncoated PEEK Cages in Lumbar Interbody Fusion: A Systematic Review and Meta-analysis of Randomized Controlled Trial. Clin Spine Surg. 2022.24:24. doi: https://dx.doi.org/10.1097/BSD.0000000000001378 | 8 | Ineligible intervention |
| 163 | Madan S, Boeree NR. Containment and stabilization of bone graft in anterior lumbar interbody fusion: the role of the Hartshill Horseshoe cage. J Spinal Disord. 2001.14(2):104-8. | 1830 | Ineligible intervention |
| 164 | Madan S, Boeree NR. Outcome of the Graf ligamentoplasty procedure compared with anterior lumbar interbody fusion with the Hartshill horseshoe cage. Eur Spine J. 2003.12(4):361-8. | 1693 | Ineligible intervention |
| 165 | Madan SS BNea. Comparison of instrumented anterior interbody fusion with instrumented circumferential lumbar fusion. Eur Spine J. 2003.12(6):567–75. | 4949 | Single level/multiple level not reported separately |
| 166 | Madan SS, Harley JM, Boeree NR. Anterior lumbar interbody fusion: does stable anterior fixation matter? Eur Spine J. 2003.12(4):386-92. | 1713 | Ineligible intervention |
| 167 | Mahatthanatrakul A, Kotheeranurak V, Lin GX, Hur JW, Chung HJ, Lokanath YK, et al. Do Obliquity and Position of the Oblique Lumbar Interbody Fusion Cage Influence the Degree of Indirect Decompression of Foraminal Stenosis? J. 2022.65(1):74-83. doi: https://dx.doi.org/10.3340/jkns.2021.0105 | 2341 | Single level/multiple level not reported separately |
| 168 | Malham GM, Giles GG, Milne RL, Blecher CM, Brazenor GA. Bone morphogenetic proteins in spinal surgery: What is the fusion rate and do they cause cancer? Spine. 2015.40(22):1737-42. doi: https://dx.doi.org/10.1097/BRS.0000000000001184 | 735 | Ineligible patient population |
| 169 | Malham GM, Parker RM, Blecher CM, Chow FY, Seex KA. Choice of Approach Does Not Affect Clinical and Radiologic Outcomes: A Comparative Cohort of Patients Having Anterior Lumbar Interbody Fusion and Patients Having Lateral Lumbar Interbody Fusion at 24 Months. Global spine j. 2016.6(5):472-81. doi: https://dx.doi.org/10.1055/s-0035-1569055 | 2137 | Off label use of rhBMP |
| 170 | Malik AT, Phillips FM, Yu E, Khan SN. Are current DRG-based bundled payment models for lumbar fusions risk-adjusting adequately? An analysis of Medicare beneficiaries. Spine J. 2020.20(1):32-40. doi: https://dx.doi.org/10.1016/j.spinee.2019.04.024 | 390 | Single level/multiple level not reported separately |
| 171 | Mamuti M et al. Mini-open anterior lumbar interbody fusion for recurrentl lumbar disc herniation following posterior instrumentation. Spine. 2016.41(18):E1104–E14. | 4951 | Ineligible patient population |
| 172 | Manzur M, Virk SS, Jivanelli B, Vaishnav AS, McAnany SJ, Albert TJ, et al. The rate of fusion for stand-alone anterior lumbar interbody fusion: a systematic review. Spine J. 2019.19(7):1294-301. doi: https://dx.doi.org/10.1016/j.spinee.2019.03.001 | 424 | SR to check |
| 173 | Marchi L, Oliveira L, Coutinho E, Pimenta L. A prospective, randomized, controlled clinical and radiological study to evaluate and compare the use of silicated calcium phosphate and rh-bmp2 in interbody lumbar spine fusion. 36 month follow-up. Eur Spine J. 2011.(4):S514. doi: 10.1007/s00586-011-1952-x | 3485 | conference abstract |
| 174 | Martin BI, Franklin GM, Deyo RA, Wickizer TM, Lurie JD, Mirza SK. How do coverage policies influence practice patterns, safety, and cost of initial lumbar fusion surgery? A population-based comparison of workers' compensation systems. Spine J. 2014.14(7):1237-46. doi: https://dx.doi.org/10.1016/j.spinee.2013.08.018 | 864 | ALIF/OLIF not reported separately |
| 175 | Martin BI, Lurie JD, Tosteson ANA, Deyo RA, Farrokhi FR, Mirza SK. Use of bone morphogenetic protein among patients undergoing fusion for degenerative diagnoses in the United States, 2002 to 2012. Spine J. 2015.15(4):692-9. doi: https://dx.doi.org/10.1016/j.spinee.2014.12.010 | 775 | Ineligible patient population |
| 176 | Mayer HM, Wiechert K. Microsurgical anterior approaches to the lumbar spine for interbody fusion and total disc replacement. Neurosurg. 2002.51(5 Suppl):159-65. doi: https://dx.doi.org/10.1097/00006123-200211002-00022 | 2692 | Ineligible patient population |
| 177 | McAfee PC, Cunningham B, Holsapple G, Adams K, Blumenthal S, Guyer RD, et al. A prospective, randomized, multicenter Food and Drug Administration investigational device exemption study of lumbar total disc replacement with the CHARITE artificial disc versus lumbar fusion: part II: evaluation of radiographic outcomes and correlation of surgical technique accuracy with clinical outcomes. Spine. 2005.30(14):1576-83; discussion E388-90. | 1586 | Ineligible intervention |
| 178 | McAfee PC, Fedder IL, Saiedy S, Shucosky EM, Cunningham BW. SB Charite disc replacement: report of 60 prospective randomized cases in a US center. J Spinal Disord Tech. 2003.16(4):424-33. | 1700 | Ineligible intervention |
| 179 | McAfee PC, Geisler FH, Saiedy SS, Moore SV, Regan JJ, Guyer RD, et al. Revisability of the CHARITE Artificial Disc replacement: Analysis of 688 patients enrolled in the U.S. IDE study of the CHARITE Artificial Disc. Spine. 2006.31(11):1217-26. doi: https://dx.doi.org/10.1097/01.brs.0000217689.08487.a8 | 3003 | Ineligible intervention |
| 180 | McConnell J. A comparison of b-tcpdbmaversus rhBMP-2 in anterior lumbar interbody fusion: a prospective, randomized trial with two-year clinical and radiographic outcomes. Spine J. 2011.(10 Suppl 1):64s‐65s. doi: 10.1016/j.spinee.2011.08.163 | 3427 | conference abstract |
| 181 | McConnell J. A comparison of ß-TCP+BMA versus RhBMP-2 in anterior lumbar interbody fusion: a prospective, randomized trial with two-year clinical and radiographic outcomes. Spine J. 2011.64s‐5s. | 3540 | conference abstract |
| 182 | McGrath M, Feroze AH, Nistal D, Robinson E, Saigal R. Impact of surgeon rhBMP-2 cost awareness on complication rates and health system costs for spinal arthrodesis. Neurosurg Focus. 2021.50(6):E5. doi: https://dx.doi.org/10.3171/2021.3.FOCUS2152 | 268 | ALIF/OLIF not reported separately |
| 183 | McGuire KJ. Graft resorption with the use of bone morphogenetic protein: Lessons from anterior lumbar interbody fusion using femoral ring allografts and recombinant human bone morphogenetic protein-2. Pradhan B, Bae H, Dawson E, Patel V, Delamarter R. Spine. 2006;31:E277-E284. Clin Orthop. 2007.454:287. doi: https://dx.doi.org/10.1097/BLO.0b013e31802ee4c0 | 2501 | conference abstract |
| 184 | Medtronic Spinal and Biologics. A pilot study of rhBMP-2/ACS with the INTERFIX™ device for the anterior lumbar interbody fusion in patients with degenerative disc disease. Identifier: NCT01491451. In: ClinicalTrials.gov [internet]. Bethesda: US National Library of Medicine: 1999. Available from https://ClinicalTrials.gov/show/NCT01491451. | 3739 | Trial record >10 years, no results posted |
| 185 | Medtronic Spinal and Biologics. A pilot study of rhBMP-2/ACS/Allograft Bone Dowel for anterior lumbar interbody fusion in patients with symptomatic degenerative disc disease. Identifier: NCT01494428. In: ClinicalTrials.gov [internet]. Bethesda: US National Library of Medicine: 1998. Available from https://ClinicalTrials.gov/show/NCT01494428. | 3732 | Off label use of rhBMP |
| 186 | Medtronic Spinal and Biologics. A pivotal study of rhBMP-2/ACS/LT-CAGE® device for anterior lumbar interbody fusion in patients with degenerative disc disease. Identifier: NCT01491425. In: ClinicalTrials.gov [internet]. Bethesda: US National Library of Medicine: 1998. Available from https://ClinicalTrials.gov/show/NCT01491425. | 3719 | Off label use of rhBMP |
| 187 | Medtronic Spinal and Biologics. A post market surveillance on INFUSE bone graft. Identifier: NCT05299762. In: ClinicalTrials.gov [internet]. Bethesda: US National Library of Medicine: 2022. Available from https://ClinicalTrials.gov/show/NCT05299762. | 3743 | Ongoing trial |
| 188 | Medtronic Spinal and Biologics. InductOs® in real world spine surgery; A retrospective, French, multi-centric, study. Identifier: NCT02280187. In: ClinicalTrials.gov [internet]. Bethesda: US National Library of Medicine: 2014. Available from https://ClinicalTrials.gov/show/NCT02280187. | 3769 | ALIF/OLIF not reported separately |
| 189 | Medtronic Spinal and Biologics. Pivotal study of rhBMP-2/ACS/Allograft Bone Dowel for anterior lumbar interbody fusion in patients with symptomatic degenerative disc disease. Identifier: NCT01494493. In: ClinicalTrials.gov [internet]. Bethesda: US National Library of Medicine: 2000. Available from https://ClinicalTrials.gov/show/NCT01494493. | 3731 | Ineligible outcomes |
| 190 | Medtronic Spinal and Biologics. rhBMP-2/CRM/CD HORIZON® spinal system pivotal study. Identifier: NCT00707265. In: ClinicalTrials.gov [internet]. Bethesda: US National Library of Medicine: 2002. Available from https://ClinicalTrials.gov/show/NCT00707265. | 3746 | Ineligible intervention |
| 191 | Mehren C, Mayer HM, Zandanell C, Siepe CJ, Korge A. The oblique anterolateral approach to the lumbar spine provides access to the lumbar spine with few early complications. Clin Orthop Relat Res. 2016.474(9):2020-27. doi: https://dx.doi.org/10.1007/s11999-016-4883-3 | 3223 | Single level/multiple level not reported separately |
| 192 | Meir AR, Freeman BJC, Fraser RD, Fowler SM. Ten-year survival and clinical outcome of the AcroFlex lumbar disc replacement for the treatment of symptomatic disc degeneration. Spine J. 2013.13(1):13-21. doi: https://dx.doi.org/10.1016/j.spinee.2012.12.008 | 3170 | Ineligible intervention |
| 193 | Meng H, Gao Y, Zhao G, Sun T, Li F. Use of recombinant human bone morphogenetic protein-2 with iliac crest bone graft instead of iliac crest bone graft alone in lumbar spondylolysis. Clin Spine Surg. 2022.35(2):E314-E19. doi: https://dx.doi.org/10.1097/BSD.0000000000001251 | 219 | Ineligible intervention |
| 194 | Mesfin A, Buchowski JM, Zebala LP, Bakhsh WR, Aronson AB, Fogelson JL, et al. High-dose rhBMP-2 for adults: major and minor complications: a study of 502 spine cases. J Bone Joint Surg Am. 2013.95(17):1546-53. doi: https://dx.doi.org/10.2106/JBJS.L.01730 | 898 | Ineligible patient population |
| 195 | Mines D, Gu Y, Kou TD, Cooper GS. Recombinant human bone morphogenetic protein-2 and pancreatic cancer: a retrospective cohort study. Pharmacoepidemiol Drug Saf. 2011.20(2):111-8. doi: https://dx.doi.org/10.1002/pds.2057 | 1207 | Surgical route not reported |
| 196 | Mobbs RJ, Amin T, Phan K, Al Khawaja D, Choy WJ, Parr WCH, et al. Standalone titanium/polyetheretherketone interbody cage for anterior lumbar interbody fusion: Clinical and radiological results at 24 months. J Craniovertebr Junction Spine. 2022.13(1):42-47. doi: https://dx.doi.org/10.4103/jcvjs.jcvjs_133_21 | 79 | Ineligible patient population |
| 197 | Mobbs RJ, Maharaj M, Rao PJ. Clinical outcomes and fusion rates following anterior lumbar interbody fusion with bone graft substitute i-FACTOR, an anorganic bone matrix/P-15 composite. J Neurosurg Spine. 2014.21(6):867-76. doi: https://dx.doi.org/10.3171/2014.9.SPINE131151 | 795 | Ineligible intervention |
| 198 | Mobbs RJ, Phan K, Assem Y, Pelletier M, Walsh WR. Combination Ti/PEEK ALIF cage for anterior lumbar interbody fusion: Early clinical and radiological results. J Clin Neurosci. 2016.34:94-99. doi: https://dx.doi.org/10.1016/j.jocn.2016.05.028 | 671 | Single level/multiple level not reported separately |
| 199 | Mobbs RJ, Phan K, Thayaparan GK, Rao PJ. Anterior lumbar interbody fusion as a salvage technique for pseudarthrosis following posterior lumbar fusion surgery. Glob Spine J. 2015.6(1):14-20. doi: https://dx.doi.org/10.1055/s-0035-1555656 | 2010 | <10 patients |
| 200 | Moreau PE, Flouzat-Lachaniette CH, Lebhar J, Mirouse G, Poignard A, Allain J. Particularities of anterior fusion in L4-L5 isthmic spondylolisthesis. Orthopaedics and Traumatology: Surgery and Research. 2016.102(6):755-58. doi: https://dx.doi.org/10.1016/j.otsr.2016.05.006 | 2820 | Ineligible patient population |
| 201 | Moura DL, Lawrence D, Gabriel JP. Multilevel anterior lumbar interbody fusion combined with posterior stabilization in lumbar disc disease-prospective analysis of clinical and functional outcomes. Rev Bras Ortop. 2019.54(2):140-48. doi: https://dx.doi.org/10.1016/j.rbo.2017.11.006 | 587 | Single level/multiple level not reported separately |
| 202 | Murphy ME, McCutcheon BA, Grauberger J, Shepherd D, Maloney PR, Rinaldo L, et al. Allograft versus autograft in cervical and lumbar spinal fusions: an examination of operative time, length of stay, surgical site infection, and blood transfusions. J Neurosurg Sci. 2019.63(1):11-18. doi: https://dx.doi.org/10.23736/S0390-5616.16.03847-9 | 451 | Ineligible patient population |
| 203 | NCT05504499. REFINE Study: A Study In a Novel Interspinous Fusion Device In Subjects With Low Back Pain. In; 2021 | 3925 | Ineligible intervention |
| 204 | Niemeyer T, Bovingloh AS, Halm H, Liljenqvist U. Results after anterior-posterior lumbar spinal fusion: 2-5 years follow-up. Int Orthop. 2004.28(5):298-302. | 1623 | Ineligible patient population |
| 205 | Norheim EP, Royse KE, Brara HS, Moller DJ, Suen PW, Rahman SU, et al. PLF+PS or ALIF+PS: which has a lower operative nonunion rate? Analysis of a cohort of 2,061 patients from a National Spine Registry. Spine J. 2021.21(7):1118-25. doi: https://dx.doi.org/10.1016/j.spinee.2021.02.018 | 2847 | DDD not reported separately |
| 206 | Norotte G, Barrios C. Clinical and radiological outcomes after stand-alone ALIF for single L5-S1 degenerative discopathy using a PEEK cage filled with hydroxyapatite nanoparticles without bone graft. Clin Neurol Neurosurg 2018.168:24-29. doi: https://dx.doi.org/10.1016/j.clineuro.2018.01.037 | 489 | Ineligible intervention |
| 207 | Novadip Biosciences. Safety and preliminary effectiveness of NVD-001 for the treatment of low grade degenerative lumbar spondylolisthesis. Identifier: NCT03100032. In: ClinicalTrials.gov [internet]. Bethesda: US National Library of Medicine: 2017. Available from https://ClinicalTrials.gov/show/NCT03100032. | 3984 | Ineligible intervention |
| 208 | NuTech Medical Inc. Efficacy study of NuCel® in patients undergoing fusion of the lumbar spine. Identifier: NCT02023372. In: ClinicalTrials.gov [internet]. Bethesda: US National Library of Medicine: 2013. Available from https://ClinicalTrials.gov/show/NCT02023372. | 3932 | Trial record >10 years, no results posted |
| 209 | NuVasive. Osteocel® plus in anterior lumbar interbody fusion (ALIF). Identifier: NCT00948831. In: ClinicalTrials.gov [internet]. Bethesda: US National Library of Medicine: 2009. Available from https://ClinicalTrials.gov/show/NCT00948831. | 3709 | Trial record >10 years, no results posted |
| 210 | NYU Langone Health. BMAC & allograft vs BMP-2. Identifier: NCT02924571. In: ClinicalTrials.gov [internet]. Bethesda: US National Library of Medicine: 2018. Available from https://ClinicalTrials.gov/show/NCT02924571. | 3761 | Ongoing trial |
| 211 | Ohtori S, Koshi T, Yamashita M, Takaso M, Yamauchi K, Inoue G, et al. Single-level instrumented posterolateral fusion versus non-instrumented anterior interbody fusion for lumbar spondylolisthesis: a prospective study with a 2-year follow-up. J Orthop Sci. 2011.16(4):352-8. doi: https://dx.doi.org/10.1007/s00776-011-0088-5 | 1185 | Ineligible intervention |
| 212 | Ohtori S, Mannoji C, Orita S, Yamauchi K, Eguchi Y, Ochiai N, et al. Mini-open anterior retroperitoneal lumbar interbody fusion: Oblique lateral interbody fusion for degenerated lumbar spinal kyphoscoliosis. Asian Spine J. 2015.9(4):565-72. doi: https://dx.doi.org/10.4184/asj.2015.9.4.565 | 1011 | Ineligible patient population |
| 213 | Ohtori S, Orita S, Yamauchi K, Eguchi Y, Ochiai N, Kishida S, et al. Mini-open anterior retroperitoneal lumbar interbody fusion: Oblique lateral interbody fusion for lumbar spinal degeneration disease. Yonsei Med J. 2015.56(4):1051-9. doi: https://dx.doi.org/10.3349/ymj.2015.56.4.1051 | 751 | Ineligible patient population |
| 214 | Ong KL, Villarraga ML, Lau E, Carreon LY, Kurtz SM, Glassman SD. Off-label use of bone morphogenetic proteins in the United States using administrative data. Spine. 2010.35(19):1794-800. doi: https://dx.doi.org/10.1097/BRS.0b013e3181ecf6e4 | 1242 | Ineligible patient population |
| 215 | Pankowski R, Smoczynski A, Jaskolski D, Roclawski M, Samson L, Piotrowski M. The comparison of the use of cages with the use of autogenous bone grafts in the operative treatment of the isthmic spondylolisthesis by the posterior stabilisation and ALIF. Chir Narzadow Ruchu Ortop Pol. 2009.(1):35‐40. | 3511 | Non-English language |
| 216 | Pankowski R, Smoczynski A, Roclawski M, Ceynowa M, Kloc W, Wasilewski W, et al. Operative treatment of isthmic spondylolisthesis with posterior stabilization and ALIF. Cages versus autogenous bone grafts. Stud Health Technol Inform. 2012.176:311-4. | 1070 | Ineligible patient population |
| 217 | Pappou IP, Papadopoulos EC, Sama AA, Girardi FP, Cammisa FP. Postoperative infections in interbody fusion for degenerative spinal disease. Clin Orthop. 2006.444:120-8. | 1543 | Ineligible intervention |
| 218 | Park JB, Yang JH, Chang DG, Suk SI, Suh SW, Kim GU, et al. Comparison of union rates between autogenous iliac crest bone graft and local bone graft as fusion materials in lumbar fusion surgery: an evaluation of up to three-level fusion. World Neurosurg. 2020.139:e286-e92. doi: https://dx.doi.org/10.1016/j.wneu.2020.03.211 | 2252 | Ineligible patient population |
| 219 | Park SC, Chang SY, Mok S, Kim H, Chang BS, Lee CK. Risk factors for postoperative ileus after oblique lateral interbody fusion: a multivariate analysis. Spine J. 2021.21(3):438-45. doi: https://dx.doi.org/10.1016/j.spinee.2020.10.002 | 3016 | Single level/multiple level not reported separately |
| 220 | Patel VV, Estes S, Lindley EM, Burger E. Lumbar spinal fusion versus anterior lumbar disc replacement: the financial implications. J Spinal Disord Tech. 2008.21(7):473-6. doi: https://dx.doi.org/10.1097/BSD.0b013e3181592264 | 1388 | Ineligible patient population |
| 221 | Pavlov PW, Meijers H, van Limbeek J, Jacobs WCH, Lemmens JAM, Obradov-Rajic M, de Kleuver M. Good outcome and restoration of lordosis after anterior lumbar interbody fusion with additional posterior fixation. Spine. 2004.29(17):1893-9. | 1619 | Ineligible intervention |
| 222 | Pavlov PW, Spruit M, Havinga M, Anderson PG, van Limbeek J, Jacobs WC. Anterior lumbar interbody fusion with threaded fusion cages and autologous bone grafts. Eur Spine J. 2000.9(3):224-9. | 1855 | <10 patients |
| 223 | Pellise F, Puig O, Rivas A, Bago J, Villanueva C. Low fusion rate after L5-S1 laparoscopic anterior lumbar interbody fusion using twin stand-alone carbon fiber cages. Spine. 2002.27(15):1665-9. doi: 10.1097/00007632-200208010-00015 | 4972 | Ineligible intervention |
| 224 | Phan K, Hogan JA, Assem Y, Mobbs RJ. PEEK-Halo effect in interbody fusion. J Clin Neurosci. 2016.24:138-40. doi: https://dx.doi.org/10.1016/j.jocn.2015.07.017 | 726 | <10 patients |
| 225 | Phillips FM, Falahee M, Wetzel F, Rabinowitz R, Gupta P. Lumbar interbody fusion comparing allograft bone dowels to threaded titanium cages - A prospective, randomized, multi-center study. Eur Spine J. 2001.(7):S67. | 3553 | conference abstract |
| 226 | Pimenta L, Pesantez CFA, Oliveira L. Silicon Matrix Calcium Phosphate as a Bone Substitute: Early Clinical and Radiological Results in a Prospective Study With 12-Month Follow-up. Sas J. 2008.2(2):62-8. doi: https://dx.doi.org/10.1016/SASJ-2007-0122-RR | 1137 | Ineligible patient population |
| 227 | Pradhan BB, Bae HW, Dawson EG, Patel VV, Delamarter RB. Graft resorption with the use of bone morphogenetic protein: lessons from anterior lumbar interbody fusion using femoral ring allografts and recombinant human bone morphogenetic protein-2. Spine. 2006.31(10):E277-84. | 1537 | Off label use of rhBMP |
| 228 | Putzier M, Strube P, Funk JF, Gross C, Monig H-J, Perka C, Pruss A. Allogenic versus autologous cancellous bone in lumbar segmental spondylodesis: a randomized prospective study. Eur Spine J. 2009.18(5):687-95. doi: https://dx.doi.org/10.1007/s00586-008-0875-7 | 1361 | Ineligible intervention |
| 229 | Quirno M, Kamerlink JR, Goldstein JA, Spivak JM, Bendo JA, Errico TJ. Outcomes analysis of anterior-posterior fusion for low grade isthmic spondylolisthesis. Bull NYU Hosp Jt Dis. 2011.69(4):316-9. | 1155 | Ineligible intervention |
| 230 | Rahn KA, Shugart RM, Wylie MW, Reddy KK, Morgan JA. The effect of lordosis, disc height change, subsidence, and transitional segment on stand-alone anterior lumbar interbody fusion using a nontapered threaded device. Am J Orthop. 2010.39(12):E124-9. | 4961 | Ineligible intervention |
| 231 | Rao PJ, Ghent F, Phan K, Lee K, Reddy R, Mobbs RJ. Stand-alone anterior lumbar interbody fusion for treatment of degenerative spondylolisthesis. J Clin Neurosci. 2015.22(10):1619-24. doi: 10.1016/j.jocn.2015.03.034 | 4962 | Mixed interventions |
| 232 | Rao PJ, Loganathan A, Yeung V, Mobbs RJ. Outcomes of anterior lumbar interbody fusion surgery based on indication: a prospective study. Neurosurgery. 2015.76(1):7-23; discussion 23-4. doi: 10.1227/NEU.0000000000000561 | 4973 | Ineligible intervention |
| 233 | Rao PJ, Phan K, Giang G, Maharaj MM, Phan S, Mobbs RJ. Subsidence following anterior lumbar interbody fusion (ALIF): a prospective study. J Spine Surg. 2017.3(2):168-75. doi: 10.21037/jss.2017.05.03 | 4963 | Ineligible intervention |
| 234 | Red de Terapia Celular. Lumbar degenerative disc disease treatment with bone marrow autologous mesenchymal stem cells (MSV). Identifier: NCT02440074. In: ClinicalTrials.gov [internet]. Bethesda: US National Library of Medicine: 2011. Available from https://ClinicalTrials.gov/show/NCT02440074. | 3871 | Trial record >10 years, no results posted |
| 235 | Red de Terapia Celular. Treatment of degenerative disc disease with allogenic mesenchymal stem cells (MSV). Identifier: NCT01860417. In: ClinicalTrials.gov [internet]. Bethesda: US National Library of Medicine: 2013. Available from https://ClinicalTrials.gov/show/NCT01860417. | 3951 | Surgical route not reported |
| 236 | Rickert M, Chatterjee B, Fleege C, Rauschmann M. Prospective, randomized study evaluating bony fusion after anterior lumbar interbody fusion (ALIF) performed using a new truss technology based interbody fusion cage filled with bone marrow aspirate (BMA) and supplemental fixation. Eur Spine J. 2018.(11):2968‐. doi: 10.1007/s00586-018-5770-2 | 3489 | conference abstract |
| 237 | Rickert M, Fleege C, Papachristos I, Makowski MR, Rauschmann M, Arabmotlagh M. Clinical outcome after anterior lumbar interbody fusion with a new osteoinductive bone substitute material: A randomized clinical pilot study. Clin Spine Surg. 2019.32(7):E319-E25. doi: https://dx.doi.org/10.1097/BSD.0000000000000802 | 432 | Single level/multiple level not reported separately |
| 238 | Robertson PA, Wray AC. Natural history of posterior iliac crest bone graft donation for spinal surgery: a prospective analysis of morbidity. Spine. 2001.26(13):1473-6. | 1819 | Ineligible intervention |
| 239 | RTI Surgical. Interbody spacers with map3® cellular allogeneic bone graft in anterior or lateral lumbar interbody fusion. Identifier: NCT02628210. In: ClinicalTrials.gov [internet]. Bethesda: US National Library of Medicine: 2015. Available from https://clinicaltrials.gov/show/NCT02628210. | 4883 | Ineligible patient population |
| 240 | Safaee MM, Tenorio A, Osorio JA, Choy W, Amara D, Lai L, et al. The impact of obesity on perioperative complications in patients undergoing anterior lumbar interbody fusion. J Neurosurg Spine. 2020.1-10. doi: https://dx.doi.org/10.3171/2020.2.SPINE191418 | 19 | Single level/multiple level not reported separately |
| 241 | Salamanna F, Tschon M, Borsari V, Pagani S, Martini L, Fini M. Spinal fusion procedures in the adult and young population: a systematic review on allogenic bone and synthetic grafts when compared to autologous bone. J Mater Sci Mater Med. 2020.31(6):51. doi: https://dx.doi.org/10.1007/s10856-020-06389-3 | 349 | SR to check |
| 242 | Santos ERG, Goss DG, Morcom RK, Fraser RD. Radiologic assessment of interbody fusion using carbon fiber cages. Spine. 2003.28(10):997-1001. | 1715 | Single level/multiple level not reported separately |
| 243 | Saraph V, Lerch C, Walochnik N, Bach CM, Krismer M, Wimmer C. Comparison of conventional versus minimally invasive extraperitoneal approach for anterior lumbar interbody fusion. Eur Spine J. 2004.13(5):425-31. | 1646 | Ineligible patient population |
| 244 | Sarwat AM, O'Brien JP, Renton P, Sutcliffe JC. The use of allograft (and avoidance of autograft) in anterior lumbar interbody fusion: a critical analysis. Eur Spine J. 2001.10(3):237-41. | 1818 | Ineligible patient population |
| 245 | Sasso RC, Best NM, Mummaneni PV, Reilly TM, Hussain SM. Analysis of operative complications in a series of 471 anterior lumbar interbody fusion procedures. Spine. 2005.30(6):670-4. | 1595 | Ineligible patient population |
| 246 | Sasso RC, Kenneth Burkus J, LeHuec JC. Retrograde ejaculation after anterior lumbar interbody fusion: transperitoneal versus retroperitoneal exposure. Spine. 2003.28(10):1023–6. | 4952 | Ineligible patient population |
| 247 | Sasso RC, Kitchel SH, Dawson EG. A prospective, randomized controlled clinical trial of anterior lumbar interbody fusion using a titanium cylindrical threaded fusion device. Spine. 2004.29(2):113-22; discussion 21-2. | 1668 | Ineligible intervention |
| 248 | Sasso RC, LeHuec JC, Shaffrey C. Iliac crest bone graft donor site pain after anterior lumbar interbody fusion: a prospective patient satisfaction outcome assessment. J Spinal Disord Tech. 2005.18 Suppl:S77-81. | 1604 | Ineligible intervention |
| 249 | Sathe A, Lee S-H, Kim S-J, Eun SS, Choi YS, Lee S-M, et al. Comparative analysis of ABM/P-15, bone morphogenic protein and demineralized bone matrix after instrumented lumbar interbody fusion. J Korean Neurosurg Soc. 2022.65(6):825-33. doi: https://dx.doi.org/10.3340/jkns.2021.0296 | 73 | ALIF/OLIF not reported separately |
| 250 | Savage JW, Kelly MP, Ellison SA, Anderson PA. A population-based review of bone morphogenetic protein: associated complication and reoperation rates after lumbar spinal fusion. Neurosurg Focus. 2015.39(4):E13. doi: https://dx.doi.org/10.3171/2015.7.FOCUS15240 | 740 | Ineligible patient population |
| 251 | Schiffman M, Brau SA, Henderson R, Gimmestad G. Bilateral implantation of low-profile interbody fusion cages: subsidence, lordosis, and fusion analysis. Spine J. 2003.3(5):377-87. | 1681 | Ineligible intervention |
| 252 | Scott-Young M, McEntee L, Furness J, Schram B, Hing W, Grosser D, Zotti M. Combined aorto-Iliac and anterior lumbar spine reconstruction: A case series. Int J Spine Surg. 2018.12(3):328-36. doi: https://dx.doi.org/10.14444/5038 | 614 | <10 patients |
| 253 | Scott-Young M, McEntee L, Schram B, Rathbone E, Hing W, Nielsen D. Concurrent use of lumbar total disc arthroplasty and anterior lumbar interbody fusion: The lumbar hybrid procedure for the treatment of multilevel symptomatic degenerative disc disease: A prospective study. Spine. 2018.43(2):E75-E81. doi: https://dx.doi.org/10.1097/BRS.0000000000002263 | 2275 | Ineligible patient population |
| 254 | Sethi A, Craig J, Bartol S, Chen W, Jacobson M, Coe C, Vaidya R. Radiographic and CT evaluation of recombinant human bone morphogenetic protein-2-assisted spinal interbody fusion. AJR Am J Roentgenol. 2011.197(1):W128-33. doi: https://dx.doi.org/10.2214/AJR.10.5484 | 1176 | ALIF/OLIF not reported separately |
| 255 | Sewon Cellontech Co Ltd. Efficacy and safety of SurgiFill™ on spinal fusion. Identifier: NCT02466048. In: ClinicalTrials.gov [internet]. Bethesda: US National Library of Medicine: 2013. Available from https://ClinicalTrials.gov/show/NCT02466048. | 3987 | Ineligible intervention |
| 256 | Shahrestani S, Ballatori AM, Chen X, Ton A, Wang JC, Buser Z. The impact of osteobiologic subtype selection on perioperative complications and hospital-reported charges in single- And multi-level lumbar spinal fusion. Int J Spine Surg. 2021.15(4):654-62. doi: https://dx.doi.org/10.14444/8086 | 3210 | Surgical route not reported |
| 257 | Shellock FG, Hatfield M, Simon BJ, Block S, Wamboldt J, Starewicz PM, Punchard WFB. Implantable spinal fusion stimulator: Assessment of MR safety and artifacts. J Magn Reson Imaging. 2000.12(2):214-23. doi: https://dx.doi.org/10.1002/1522-2586%28200008%2912:2%3C214::AID-JMRI2%3E3.0.CO;2-K | 2552 | <10 patients |
| 258 | Singh K, Cha EDK, Lynch CP, Nolte MT, Parrish JM, Jenkins NW, et al. Risk assessment of anterior lumbar interbody fusion access in degenerative spinal conditions. Clin Spine Surg. 2022.35(7):E601-E09. doi: https://dx.doi.org/10.1097/BSD.0000000000001322 | 197 | Single level/multiple level not reported separately |
| 259 | Singh K, Nandyala SV, Marquez-Lara A, Fineberg SJ. Epidemiological trends in the utilization of bone morphogenetic protein in spinal fusions from 2002 to 2011. Spine. 2014.39(6):491-6. doi: https://dx.doi.org/10.1097/BRS.0000000000000167 | 854 | Single level/multiple level not reported separately |
| 260 | Slosar PJ, Josey R, Reynolds J. Accelerating lumbar fusions by combining rhBMP-2 with allograft bone: a prospective analysis of interbody fusion rates and clinical outcomes. Spine J. 2007.7(3):301-7. | 1479 | Off label use of rhBMP |
| 261 | Smoljanovic T, Josipovic M, Bojanic I. The justification for recombinant human bone morphogenetic protein-2 use in one- or two-level lumbar spine interbody fusions. J Clin Neurosci. 2011.18(3):445-6. doi: https://dx.doi.org/10.1016/j.jocn.2010.05.020 | 1209 | Ineligible study design |
| 262 | Smoljanovic T, Pecina M. Re: Burkus J K, Transfeldt E E, Kitchel S H, et al. Clinical and radiographic outcomes of anterior lumbar interbody fusion using recombinant human bone morphogenetic protein-2. (Spine 2002;27:2396-408). Spine. 2008.33(2):224. doi: https://dx.doi.org/10.1097/BRS.0b013e31816068e2 | 1433 | Ineligible study design |
| 263 | Smoljanovic T, Siric F, Bojanic I. Six-year outcomes of anterior lumbar interbody arthrodesis with use of interbody fusion cages and recombinant human bone morphogenetic protein-2. J Bone Joint Surg Am. 2010.92(15):2614-5. | 1232 | Ineligible study design |
| 264 | Snowden R, Fischer D, Kraemer P. Early outcomes and safety of outpatient (surgery center) vs inpatient based L5-S1 Anterior Lumbar Interbody Fusion. J Clin Neurosci. 2020.73:183-86. doi: https://dx.doi.org/10.1016/j.jocn.2019.11.001 | 2363 | Off label use of rhBMP |
| 265 | Sofamor - Danek. A randomized prospective study of instrumented posterior lumbar interbody fusion: Autologous iliac crest bone graft vs. Inductos™. Identifier: EUCTR2005-005766-36-BE. In: EU Clinical Trials Register [internet]. Amsterdam: European Medicines Agency: 2006. Available from https://www.clinicaltrialsregister.eu/ctr-search/search?query=eudract_number:2005-005766-36. | 4909 | Ineligible intervention |
| 266 | Spineology Inc. OptiMesh® for lumbar interbody fusion trial (OLIF). Identifier: NCT00764491. In: ClinicalTrials.gov [internet]. Bethesda: US National Library of Medicine: 2003. Available from https://ClinicalTrials.gov/show/NCT00764491. | 3877 | Ineligible intervention |
| 267 | Stephan SR, Kanim LE, Bae HW. Stem cells and spinal fusion. Int J Spine Surg. 2021.15(1):94-103. doi: https://dx.doi.org/10.14444/8057 | 3113 | SR to check |
| 268 | Subach BR, Copay AG, Martin MM, Schuler TC. Anterior lumbar interbody implants: importance of the interdevice distance. Adv Orthop. 2011.2011:176497. doi: https://dx.doi.org/10.4061/2011/176497 | 1123 | Ineligible patient population |
| 269 | Svante Berg. A study comparing the outcome of anterior lumbar fusion and total lumbar disc replacement. Identifier: NCT03674190. In: ClinicalTrials.gov [internet]. Bethesda: US National Library of Medicine: 2018. Available from https://ClinicalTrials.gov/show/NCT03674190. | 4123 | Ongoing trial |
| 270 | Svedbom A, Paech D, Leonard C, Donnell D, Song F, Boszcyk B, et al. Is dibotermin alfa a cost-effective substitute for autologous iliac crest bone graft in single level lumbar interbody spine fusion? Curr Med Res Opin. 2015.31(11):2145-56. doi: https://dx.doi.org/10.1185/03007995.2015.1092123 | 742 | Surgical route not reported |
| 271 | Szadkowski M, Bahroun S, Aleksic I, Vande Kerckhove M, Ramos-Pascual S, Fiere V, d'Astorg H. Clinical and radiologic outcomes of stand-alone anterior lumbar interbody fusion at L4-L5. Interdiscip Neurosurg. 2021.26:101312. doi: https://dx.doi.org/10.1016/j.inat.2021.101312 | 2150 | Mixed interventions |
| 272 | Szadkowski M, Bahroun S, Aleksic I, Vande Kerckhove M, Ramos-Pascual S, Saffarini M, et al. Bioactive glass grants equivalent fusion compared to autologous iliac crest bone for ALIF: a within-patient comparative study. J Exp Orthop. 2022.9(1):56. doi: https://dx.doi.org/10.1186/s40634-022-00496-6 | 69 | Single level/multiple level not reported separately |
| 273 | Szadkowski M, d'Astorg H, Bouhali H, Aleksic I, Ramos-Pascual S, Fiere V. Outcomes of stand-alone anterior lumbar interbody fusion of L5-S1 using a novel implant with anterior plate fixation. Spine J. 2020.20(10):1618-28. doi: https://dx.doi.org/10.1016/j.spinee.2020.05.555 | 2814 | Mixed interventions |
| 274 | Tepper G, Rabbani R, Yousefzadeh M, Prince D. Quantitative assessment of retrograde ejaculation using semen analysis, comparison with a standardized qualitative questionnaire, and investigating the impact of rhBMP-2. Spine. 2013.38(10):841-5. doi: https://dx.doi.org/10.1097/BRS.0b013e31828bf36a | 940 | Single level/multiple level not reported separately |
| 275 | Thalgott JS, Giuffre JM, Klezl Z, Timlin M. Anterior lumbar interbody fusion with titanium mesh cages, coralline hydroxyapatite, and demineralized bone matrix as part of a circumferential fusion. Spine J. 2002.2(1):63-9. | 1737 | Single level/multiple level not reported separately |
| 276 | Theologis AA, Patel S, Burch S. Radiographic comparison of L5-S1 lateral anterior lumbar interbody fusion cage subsidence and displacement by fixation strategy: anterior plate versus integrated screws. J Neurosurg Pediatr: Spine. 2023.38(1):126-30. doi: https://dx.doi.org/10.3171/2022.7.SPINE22436 | 2944 | Off label use of rhBMP |
| 277 | Tong Y-J, Liu J-H, Fan S-W, Zhao F-D. One-stage debridement via oblique lateral interbody fusion corridor combined with posterior pedicle screw fixation in treating spontaneous lumbar infectious spondylodiscitis: A case series. Orthop Surg. 2019.11(6):1109-19. doi: https://dx.doi.org/10.1111/os.12562 | 395 | Ineligible patient population |
| 278 | Tong YJ, Liu JH, Fan SW, Zhao FD. One-stage debridement via oblique lateral interbody fusion corridor combined with posterior pedicle screw fixation in treating spontaneous lumbar infectious spondylodiscitis: A case series. Orthop Surg. 2019.doi: https://dx.doi.org/10.1111/os.12562 | 2784 | Ineligible patient population |
| 279 | Tristate Brain and Spine Institute. Clinical and radiological outcomes of oblique lateral lumbar interbody fusion. Identifier: NCT03726190. In: ClinicalTrials.gov [internet]. Bethesda: US National Library of Medicine: 2013. Available from https://ClinicalTrials.gov/show/NCT03726190. | 4219 | Ineligible intervention |
| 280 | University Hospital Ghent. Long-term follow-up of the anterior lumbar interbody fusion procedure. Identifier: NCT01666860. In: ClinicalTrials.gov [internet]. Bethesda: US National Library of Medicine: 2010. Available from https://ClinicalTrials.gov/show/NCT01666860. | 3966 | Single level/multiple level not reported separately |
| 281 | University Hospitals Cleveland Medical Center. Mesenchymal stem cells for lumbar degenerative disc disease. Identifier: NCT03692221. In: ClinicalTrials.gov [internet]. Bethesda: US National Library of Medicine: 2019. Available from https://ClinicalTrials.gov/show/NCT03692221. | 3971 | Ongoing trial |
| 282 | University of Kansas Medical Center. Irrisept spinal fusion pilot study protocol. Identifier: NCT05011942. In: ClinicalTrials.gov [internet]. Bethesda: US National Library of Medicine: 2021. Available from https://ClinicalTrials.gov/show/NCT05011942. | 3937 | Ineligible intervention |
| 283 | Vaidya R, Sethi A, Bartol S, Jacobson M, Coe C, Craig JG. Complications in the use of rhBMP-2 in PEEK cages for interbody spinal fusions. J Spinal Disord Tech. 2008.21(8):557-62. doi: https://dx.doi.org/10.1097/BSD.0b013e31815ea897 | 1381 | Off label use of rhBMP |
| 284 | Vaidya R, Weir R, Sethi A, Meisterling S, Hakeos W, Wybo CD. Interbody fusion with allograft and rhBMP-2 leads to consistent fusion but early subsidence. Journal of Bone and Joint Surgery - British Volume. 2007.89(3):342-5. | 1487 | Single level/multiple level not reported separately |
| 285 | Varshneya K, Medress ZA, Jensen M, Azad TD, Rodrigues A, Stienen MN, et al. Trends in anterior lumbar interbody fusion in the United States: A MarketScan study from 2007 to 2014. Clin Spine Surg. 2020.33(5):E226-E30. doi: https://dx.doi.org/10.1097/BSD.0000000000000904 | 386 | Single level/multiple level not reported separately |
| 286 | Verbruggen D, Tampere T, Uyttendaele D, Sys G, Poffyn B. Long-term follow-up of the anterior lumbar interbody fusion procedure. Acta Orthop Belg. 2015.81(3):546-52. | 4965 | Ineligible intervention |
| 287 | Vincentelli AF, Szadkowski M, Vardon D, Litrico S, Fuentes S, Steib JP, et al. Description of the use of rhBMP2 in spine surgery: a retrospective, French, multi-center study. In: European Spine Journal. EUROSPINE meeting., Germany; 2016. S361 | 3473 | conference abstract |
| 288 | Vincentelli AF, Szadkowski M, Vardon D, Litrico S, Fuentes S, Steib J-P, et al. rhBMP-2 (Recombinant Human Bone Morphogenetic Protein-2) in real world spine surgery. A phase IV, national, multicentre, retrospective study collecting data from patient medical files in French spinal centres. OTSR. 2019.105(6):1157-63. doi: https://dx.doi.org/10.1016/j.otsr.2019.04.023 | 402 | Ineligible patient population |
| 289 | Virk S, Sandhu HS, Khan SN. Cost effectiveness analysis of graft options in spinal fusion surgery using a Markov model. J Spinal Disord Tech. 2012.25(7):E204-10. | 1067 | Surgical route not reported |
| 290 | Virtua Health Inc. rhBMP-2 versus Vivigen in lumbar fusion procedures. Identifier: NCT03527966. In: ClinicalTrials.gov [internet]. Bethesda: US National Library of Medicine: 2017. Available from https://ClinicalTrials.gov/show/NCT03527966. | 3714 | <10 patients |
| 291 | Vishteh AG, Dickman CA. Anterior lumbar microdiscectomy and interbody fusion for the treatment of recurrent disc herniation. Neurosurgery. 2001.48(2):334-7; discussion 38. | 1837 | <10 patients |
| 292 | Wadhwa H, Wu JY, Malacon K, Ames C, Ratliff J, Zygourakis C. Trends, payments, and costs associated with BMP use in Medicare beneficiaries undergoing ppinal fusion. Spine J. 2023.26:26. doi: https://dx.doi.org/10.1016/j.spinee.2023.01.012 | 5 | Single level/multiple level not reported separately |
| 293 | Wanderman N, Carlson B, Robinson W, Bydon M, Yaszemski M, Huddleston P, Freedman B. Does recombinant human bone morphogenic protein 2 affect perioperative blood loss after lumbar and thoracic spinal fusion? Asian Spine J. 2018.12(5):880-86. doi: https://dx.doi.org/10.31616/asj.2018.12.5.880 | 617 | Ineligible patient population |
| 294 | Wang B, Chen C, Hua W, Ke W, Lu S, Zhang Y, et al. Minimally invasive surgery oblique lumbar interbody debridement and fusion for the treatment of lumbar spondylodiscitis. Orthop Surg. 2020.12(4):1120-30. doi: https://dx.doi.org/10.1111/os.12711 | 344 | Ineligible patient population |
| 295 | Wang P, Zhang H, Liu L, Wu C, Wang H. The application of polyetheretherketone cage for lumbar fusion and the pedicle screw-based internal fixation in senile lumbar degenerative diseases. Biomed Res (India). 2017.28(5):2008-13. | 3172 | Ineligible intervention |
| 296 | West China Hospital of Sichuan University. A randomized controlled study of rhBMP-2-loaded self-curing calcium phosphate artificial bone (granular) and allogeneic bone in lateral anterior lumbar interbody fusion (OLIF). Identifier: ChiCTR2100043375. In: Chinese Clinical Trial Register [internet]. Chengdu: Chinese University of Hong Kong: 2021. Available from http://www.chictr.org.cn/showproj.aspx?proj=52426. | 4821 | Ongoing trial |
| 297 | Westerlund LE, Borden M. Clinical experience with the use of a spherical bioactive glass putty for cervical and lumbar interbody fusion. J Spine Surg,. 2020.6(1):49-61. doi: https://dx.doi.org/10.21037/jss.2020.03.06 | 149 | Ineligible intervention |
| 298 | Wetzell B, McLean JB, Dorsch K, Moore MA. A 24-month retrospective update: follow-up hospitalization charges and readmissions in US lumbar fusion surgeries using a cellular bone allograft (CBA) versus recombinant human bone morphogenetic protein-2 (rhBMP-2). J Orthop Surg. 2021.16(1):680. doi: https://dx.doi.org/10.1186/s13018-021-02829-0 | 236 | Surgical route not reported |
| 299 | Wetzell B, McLean JB, Moore MA, Kondragunta V, Dorsch K. A large database study of hospitalization charges and follow-up re-admissions in US lumbar fusion surgeries using a cellular bone allograft (CBA) versus recombinant human bone morphogenetic protein-2 (rhBMP-2). Journal of Orthopaedic Surgery. 2020.15(1):544. doi: https://dx.doi.org/10.1186/s13018-020-02078-7 | 314 | Surgical route not reported |
| 300 | Whang PG, O'Hara BJ, Ratliff J, Sharan A, Brown Z, Vaccaro AR. Pseudarthrosis following lumbar interbody fusion using bone morphogenetic protein-2: Intraoperative and histopathologic findings. Orthopedics. 2008.31(10):1031. | 2927 | Ineligible study design |
| 301 | Wind J, Park D, Lansford T, Nunley P, Peppers T, Russo A, et al. Twelve-month results from a prospective clinical study evaluating the efficacy and safety of cellular bone allograft in subjects undergoing lumbar spinal fusion. Neurology Int. 2022.14(4):875-83. doi: https://dx.doi.org/10.3390/neurolint14040070 | 38 | Single level/multiple level not reported separately |
| 302 | Woodward HR. A prospective-randomized controlled clinical trial of anterior lumbar interbody fusion using a titanium cylindrical threaded fusion device: Point of view. Spine. 2004.29(2):121-22. doi: https://dx.doi.org/10.1097/01.BRS.0000107221.86855.14 | 1939 | Ineligible study design |
| 303 | Wu H, Shan Z, Zhang T, Liu J, Fan S, Zhao F, Cheung JPY. Small preoperative dural sac cross-sectional area and anteriorly placed fusion cages are risk factors for indirect decompression failure after oblique lateral interbody fusion. World Neurosurg. 2022.167:e1032-e44. doi: https://dx.doi.org/10.1016/j.wneu.2022.08.134 | 3074 | Single level/multiple level not reported separately |
| 304 | Xie T, Wang C, Yang Z, Xiu P, Yang X, Wang X, et al. Minimally invasive oblique lateral lumbar interbody fusion combined with anterolateral screw fixation for lumbar degenerative disc disease. World Neurosurg. 2020.135:e671-e78. doi: https://dx.doi.org/10.1016/j.wneu.2019.12.105 | 378 | Ineligible intervention |
| 305 | Xie T, Xiu P, Yang Z, Wang D, Zeng J, Song Y. OLIF combined with anterior fixation for lumbar synovial cysts with instability. World Neurosurg. 2020.135:76-79. doi: https://dx.doi.org/10.1016/j.wneu.2019.11.094 | 2780 | Ineligible study design |
| 306 | Yao Q, Cohen JR, Buser Z, Park J-B, Brodke DS, Meisel H-J, et al. Analysis of recombinant human bone morphogenetic protein-2 use in the treatment of lumbar degenerative spondylolisthesis. Global Spine J. 2016.6(8):749-55. | 987 | Ineligible patient population |
| 307 | Yuan ZC, Chen YM, Chen F, Liu WX. Long-term comparison of three bone graft materials in lumbar interbody fusion for lumbar spondylolisthesis. CJTER. 2013.(16):3033‐40. doi: 10.3969/j.issn.2095-4344.2013.16.026 | 3499 | Non-English language |
| 308 | Zdeblick TA, David SM. A prospective comparison of surgical approach for anterior L4–L5 fusion: laparoscopic versus mini anterior lumbar interbody fusion. Spine. 2000.25(20):2682–7. | 4955 | Ineligible intervention |
| 309 | Zelle B, Konig F, Enderle A, Bertagnoli R, Dorner J. Circumferential fusion of the lumbar and lumbosacral spine using a carbon fiber ALIF cage implant versus autogenous bone graft: a comparative study. J Spinal Disord Tech. 2002.15(5):369-76. | 1756 | Single level/multiple level not reported separately |
| 310 | Zhang Z-C, Zhang Y, Zhang L-Z, Guan K, Zhao G-M, Ren D-J, et al. Repair of symptomatic bilateral L5 spondylolysis with autogenous iliac crest graft and temporary intersegmental pedicle screw fixation in youth. Journal of Orthopaedic Surgery. 2021.16(1):422. doi: https://dx.doi.org/10.1186/s13018-021-02534-y | 256 | Ineligible patient population |
| 311 | Zhao C-G, Qin J, Wang X, Xu G, Jia Y, Guan Y-C, et al. Clinical outcomes of treatment with cage-shaped demineralized bone plus local bone grafts vs. autogenous iliac crest bone grafts in instrumented single-level lumbar fusion: A retrospective cohort study. Exp Ther Med. 2020.19(1):393-99. doi: https://dx.doi.org/10.3892/etm.2019.8171 | 152 | Ineligible patient population |
| 312 | Zhao L, Xie T, Wang X, Yang Z, Pu X, Lu Y, Zeng J. Clinical and radiological evaluation of cage subsidence following oblique lumbar interbody fusion combined with anterolateral fixation. BMC Musculoskelet Disord. 2022.(1):214. doi: 10.1186/s12891-022-05165-4 | 3395 | Off label use of rhBMP |
| 313 | Zhou T, Gu Y. Hybrid surgery of percutaneous transforaminal endoscopic surgery (PTES) combined with OLIF and anterolateral screws rod fixation for treatment of multi-level lumbar degenerative diseases with intervertebral instability. Journal of Orthopaedic Surgery. 2023.18(1):117. doi: https://dx.doi.org/10.1186/s13018-023-03573-3 | 42 | Ineligible patient population |
| 314 | Zimmer Biomet. Post-market surveillance study of the TM Ardis interbody fusion system. Identifier: NCT02429908. In: ClinicalTrials.gov [internet]. Bethesda: US National Library of Medicine: 2014. Available from https://ClinicalTrials.gov/show/NCT02429908. | 3986 | Ongoing trial |

# Appendix E: Data Extraction Elements

- Study details (bibliographic details).
- Study characteristics:
- Study design.
- Study objective.
- Number of participating centres and countries.
- Eligibility criteria.
- Number of patients randomised/analysed.
- Follow up duration.
- Data collection time points.
- Statistical powering of the study.
- Patient baseline characteristics:
- Age – (Age > 60 and Age <60).
- Sex.
- BMI.
- Time since diagnosis.
- Vitamin D deficiency.
- Non-steroidal anti-inflammatory (NSAID) use (n/%).
- Opioid use (n/%).
- Smoker – Never, past, current.
- Alcohol use.
- Nutrition status.
- Blood sugar levels (by A1C test).
- Diabetes – Yes/No.
- Osteoporosis (as diagnosed by DEXA bone density scan).
- Number of DDD levels treated.
- Prior history of surgery at the treated level.
- Details of intervention:
- Treatment type, brand name and name of manufacturer.
- Interbody material used.
- Use of posterior instrumentation (yes/no).
- Amount of graft material used.
- Graft preparation time (if relevant).
- Graft preparation method (if relevant).
- Vertebrae being fused.
- Surgical procedure details including:
  - - Type of surgery (open or minimally invasive).
    - Operative time.
    - Blood loss.
- Details of statistical analyses.
- For each of the clinical outcomes specified we extracted the following:
- Outcome definition.
- Who assessed the outcome.
- The unit of measurement.
- The number of patients included in the analysis.
- The size of the effect:
  - - For dichotomous outcomes; absolute and relative risks (or odds ratios) and risk (or rate) differences.
    - For continuous outcomes; the mean change and measure of variance from baseline (or at both baseline and final visit), or mean difference between treatments.
    - For time-to-event analysis; the number of events in each arm at a given timepoint, median time to event and a hazard ratio and p-value.
    - Where possible, absolute and relative data will be extracted.
- A measure of precision for each estimate of effect (95% confidence intervals, standard error or standard deviation).
- For each of the PROMs outcomes specified we extracted the following:
- PROM methods:
  - - Country (where the data were collected).
    - The number of patients included in the analysis.
    - Source of perspective of the values (for example, patients, carers, hypothetical health state).
    - Was a proxy used? If yes, describe.
    - Method of elicitation (PROM tool).
    - Uncertainty around values, how was this measured?
- PROM values:
  - - Mean or median PROM values.
    - Standard error or confidence intervals or standard deviation of PROMs.
- For each of the utilities outcomes specified we extracted the following:
- Utility methods:
  - - Country (where the data were collected).
    - The number of patients included in the analysis.
    - Source of perspective of the values (for example, patients, carers, hypothetical health state).
    - Was a proxy used? If yes, describe.
    - Method of elicitation (utility tool).
    - Preference scaling (if stated, for example: time trade off (TTO), standard gamble (SG), rating scale, willingness to pay (WtP)).
    - Method of valuation, for example note if a particular tariff (UK etc.) was used.
    - Describe any mapping conducted (if relevant).
    - Uncertainty around values, how was this measured?
    - Sources of data (if a model, and the data generation is not described within the same paper, the source of the data will be described).
- Utility values:
  - - Mean or median utility or disutility values (by health state).
    - Standard error or confidence intervals or standard deviation of utilities or disutilities (including increments and decrements).
- For each of the monetary and non-monetary costs data outcomes specified we extracted the following:
- Direct and indirect costs data:
  - - Currency.
    - Cost year.
    - Costs uplifting methodology (if relevant).
    - Costs of surgery.
    - Costs of staff time.
    - Other direct costs data.
    - Other indirect costs data.
    - Source(s) of the data (if not the study itself, e.g. a database).
    - Downstream costs where reported (e.g. XX reduced adverse event was associated with YY reduction in cost for revision surgery and ZZ reduced cost for analgesics/opioids etc.).
- Healthcare resource use data:
  - - Cost year.
    - Number of days in hospital.
    - Number of work days missed.
    - Staff time.
    - Other healthcare resource use data.
    - Source(s) of the data (if not the study itself, e.g. a database).
- For each of the economic evaluations outcomes specified we extracted the following:
- Study country (model setting).
- Study perspective.
- Type of economic evaluation:
  - - Author defined.
    - Reviewer defined (i.e. as determined by the reviewer).
- Analytic approach (trial based, model based).
- Modelling methods:
  - - Time horizon.
    - Discounting (costs and effects).
    - Reference year of the analysis.
    - Currency.
    - Model type (e.g. Markov, Decision Tree, Discrete Event Simulation).
    - Model cycle lengths.
    - Model health states used.
    - Model assumptions, briefly described.
    - Main input sources:
      - Utilities/health-related quality of life (HRQoL) data:
        - Source of utilities/HRQoL data (e.g. from the literature, study elicited).
        - Mean or median data used (standard error and confidence intervals will be extracted if reported).
        - Mapping – describe how any mapping was conducted.
      - Effectiveness data.
      - Resource use and costs data – a summary of the source(s) of these data.
    - Sensitivity analyses methodology – brief description.
- Methods for non-models:
  - - Cost calculation method – brief description.
- Economic evaluation results:
  - - Costs outcomes, e.g. total costs.
    - Effectiveness outcomes e.g. QALYs.
    - Base case incremental analyses outcomes, e.g. ICERs.
    - Base case outcomes for other economic evaluation types.
    - Sensitivity analyses.

For each outcome, we collected data at all-time points reported.

# Appendix F: Risk of Bias Assessment Tables

1. Risk of bias assessment of included randomised controlled trials

Table F1. Biases arising from the randomisation process

| Study name | 1.1 Was the allocation sequence random? | 1.2 Was the allocation sequence concealed until participants were enrolled and assigned to interventions? | 1.3 Did baseline differences between intervention groups suggest a problem with the randomisation process? | Risk of bias judgement |
| --- | --- | --- | --- | --- |
| **Infuse studies** | | | | |
| Boden 2000 [24] | No information | No information | Probably no | Some concerns |
|  | Described as randomised but no details reported | No details reported | The authors report that the groups were similar at baseline. The only variable that was statistically significantly different was patient weight, with a mean of 211 lb in the control group and 166 lb in the investigational group. |  |
| Burkus 2002 [26] | No information | No information | No | Some concerns |
|  | Described as randomised but no details reported | No details reported | Study reported that there were no significant differences (p<0.05) between the treatment groups at baseline |  |
| Burkus 2002b [47] | Yes | No information | Probably no | Some concerns |
|  | Patients randomly assigned from a table of random numbers | No details reported | The groups were not compared at baseline. There appear to be some differences (male/female ratio, number with previous surgeries) but unclear whether these differences are significant. |  |
| Gornet 2011 [29] | Yes | Yes | No | Low |
|  | Patients were randomized according to a schedule centrally generated by the study sponsor with a fixed block size of six. The block size was unknown to study investigators and patients during the study. Treatment randomization was 2:1 (investigational to control) on a site basis | Sequentially numbered, sealed envelopes were provided by the study sponsor | The two treatment groups were similar demographically, and preoperative evaluations of clinical endpoints were similar in each treatment group. Only one comparison was different, in preoperative medication use, the investigational group had a higher percentage of patients using nonnarcotic medications before surgery (P = 0.014). |  |
| Studies of other interventions | | | | |
| Chung 2003 [35] | No information | No information | No information | Some concerns |
|  | Described as randomised but no details reported | No details reported | Gender, age and mean follow up are the only baseline details that were compared. Previous disc surgery was reported, but not compared. |  |

**Table F2. Bias due to deviations from the intended intervention (Effect of assignment to intervention)**

| Study name | 2.1 Were participants aware of their assigned intervention during the trial? | 2.2 Were carers and trial people delivering the interventions aware of the participants assigned intervention during the trial? | 2.3 If Yes/probably yes/no information to 2.1 or 2.2, were there deviations from the intended intervention that arose because of the trial context? | 2.4 If Yes/probably yes to 2.3, were these deviations likely to have affected the outcomes? | 2.5 If yes/possibly yes/no information to 2.4 Were these deviations from intended intervention balanced between groups? | 2.6 Was an appropriate analysis used to estimate the effect of assignment to intervention? | 2.7 If No/probably no/no information to 2.6. Was there potential for a substantial impact of the failure to analyse the participants in their group? | Risk of bias judgement |
| --- | --- | --- | --- | --- | --- | --- | --- | --- |
| Infuse studies | | | | | | | | |
| Boden 2000 [24] | Yes | Yes | No information | NA | NA | Probably yes | NA | Some concerns |
|  | Study is reported to be non-blinded | Study is reported to be non-blinded | The authors do not report withdrawals or deviations from the protocol |  |  | Assume ITT analysis undertaken, but this is not reported |  |  |
| Burkus 2002 [26] | Yes | Yes | No information | NA | NA | No | Yes | High risk |
|  | Study is reported to be non-blinded | Study is reported to be non-blinded | The authors do not report withdrawals or deviations from the protocol |  |  | Per protocol analyses undertaken | The study reports that the rates of follow up are high (>90% at both 12 and 24 months for both arms). However, the study starts with 143 patients in the Infuse group and ends at 24 months with 122 - a 15% loss, and starts with 136 in the ICBG group and ends with 108 - a 21% loss. No explanations are reported. |  |
| Burkus 2002b [47] | Yes | Yes | No information | NA | NA | No | Probably no | Some concerns |
|  | Study is reported to be non-blinded | Study is reported to be non-blinded | The authors do not report withdrawals or deviations from the protocol |  |  | Per protocol analyses undertaken | Only two patients without data overall, unlikely to have changed outcomes |  |
| Gornet 2011 [29] | Yes | Yes | No information | NA | NA | No | Probably yes | High risk |
|  | Both the investigator and the patient were blinded to the randomization before informed consent but were not blinded after the opening of the treatment envelope immediately following informed consent because differences in postoperative regimen would compromise the blinded status of patients | Study is non-blinded | The authors do not report reasons for withdrawals or deviations from the protocol |  |  | Per protocol analyses undertaken | Infuse: There were 172 patients randomised at baseline and 145 (84.3%) remaining at 24 months Artificial disc replacement: There were 405 patients randomised at baseline and 379 (93.6%) remaining at 24 months. No explanations for losses to follow up are reported, although it appears some patients may have died. |  |
| Studies of other interventions | | | | | | | | |
| Chung 2003 [35] | No information | No information | No information | No information | NA | No | Yes | High risk |
|  | Not reported | Not reported | Not reported | Not reported |  | Per protocol analysis undertaken | Of 54 included patients, ten (18.5%) were not included in the analysis of final outcome: three cases in group 1 were converted to open procedure and, among the rest, five were lost to follow up in group 1 and two in group 2. |  |

**Table F3. Risk of bias due to deviations from the intended interventions (Effect of adhering to intervention)**

| Study | 2.1 Were participants aware of their assigned intervention during the trial? | 2.2 Were carers and people delivering the interventions aware of the participants assigned intervention during the trial? | 2.3 [If applicable] If yes/probably yes/ no information to 2.1 or 2.2 Were important non-protocol interventions balanced across intervention groups? | 2.4 [If applicable] Were there failures in implementing the intervention that could have affected the outcome? | 2.5 [If applicable] Was there non-adherence to the assigned intervention regimen that could have affected participant’s outcomes? | 2.6. If no/probably no/no information to 2.3, or yes probably yes/no information to 2.4 or 2.5 Was an appropriate analysis used to estimate the effect of adhering to the intervention? | Risk of bias judgement |
| --- | --- | --- | --- | --- | --- | --- | --- |
| Infuse studies | | | | | | | |
| Boden 2000 [24] | Yes | Yes | No information | No information | No | Probably yes | Some concerns |
|  | Study is reported to be non-blinded | Study is reported to be non-blinded | No details about concomitant treatments are reported. | Re-operations not reported | All patients received surgery | Assume ITT analysis undertaken, but this is not reported |  |
| Burkus 2002 [26] | Yes | Yes | No information | Probably no | No | NA | Low risk |
|  | Study is reported to be non-blinded | Study is reported to be non-blinded | No details about concomitant treatments are reported. | Number of failures reported in Burkus 2003, similar number in both groups (6/143 in Infuse group, 4/136 in ICBG group). | All patients received surgery | NA |  |
| Burkus 2002b [47] | Yes | Yes | No information | Probably yes | No | No | High risk |
|  | Study is reported to be non-blinded | Study is reported to be non-blinded | No details about concomitant treatments are reported. | There were a higher number of patients in the control group requiring supplemental fixation and reoperation (n=4) compared to Infuse (n=1) | All patients received surgery | Per protocol analyses undertaken |  |
| Gornet 2011 [29] | Yes | Yes | Probably yes | Probably yes | No | No | High risk |
|  | Both the investigator and the patient were blinded to the randomization before informed consent but were not blinded after the opening of the treatment envelope immediately following informed consent because differences in postoperative regimen would compromise the blinded status of patients | Study is non-blinded | Non-protocol interventions were reported, and there was a significant difference in the use of non-narcotic medications between groups | There were a significantly lower number of re-operations in the Infuse group compared to the artificial disc replacement group p=0.0046 | All patients received surgery | Per protocol analyses undertaken |  |
| Studies of other interventions | | | | | | | |
| Chung 2003 [35] | No information | No information | No information | Yes | Yes | No | High risk |
|  | Not reported | Not reported | Non-protocol interventions not reported | In the laparoscopic group 3/27(11.1%) converted to an open procedure. | The patients that converted to open procedures were excluded | Per protocol analysis undertaken |  |

**Table F4. Bias due to missing outcome data**

| Study name | 3.1 Were outcome data available for all, or nearly all participants randomised? | 3.2 If N/PN/NI to 3.1: Is there evidence that the result was not biased by missing outcome data? | 3.3 If N/PN/NI to 3.2: Could missingness in the outcome depend on its true value? | 3.4 It Y/PY/NI to 3.3: Is it likely that missingness in the outcome depended on its true value? | Risk of bias judgement |
| --- | --- | --- | --- | --- | --- |
| Infuse studies | | | | | |
| Boden 2000 [24] | Yes | NA | NA | NA | Low risk |
|  | Data is reported for all patients at all time points |  |  |  |  |
| Burkus 2002 [26] | No | No | No information | Yes | High risk |
|  | The study starts with 143 patients in the Infuse group and ends at 24 months with 122 - a 15% loss, and starts with 136 in the ICBG group and ends with 108 - a 21% loss. No explanations are reported. | Analysis methods did not correct for bias and no sensitivity analyses undertaken | No details reported about patients who withdrew | The proportions of missing outcome data are different between the two arms |  |
| Burkus 2002b [47] | No | No | Probably no | NA | Low risk |
|  | There is no data for two patients in the control group, | No bias correction or sensitivity analyses undertaken | One patient died in a house fire and one was lost to follow up |  |  |
| Gornet 2011 [29] | No | No | No information | Yes | High risk |
|  | The study starts with 172 patients in the Infuse group and ends at 24 months with 145 - a 15.7% loss. The artificial disc replacement group starts with 405 and ends with 379 - a 6.4% loss. No explanations are reported. | Analysis methods did not correct for bias and no sensitivity analyses undertaken | No details reported about patients who withdrew | The proportions of missing outcome data are different between the two arms |  |
| Studies of other interventions | | | | | |
| Chung 2003 [35] | No | No | Probably no | NA | Some concerns |
|  | Of 54 included patients, ten (18.5%) were not included in the analysis of final outcome: three cases in group 1 were converted to open procedure and, among the rest, five were lost to follow up in group 1 and two in group 2. | Analysis methods did not correct for bias and no sensitivity analyses undertaken | Some participants were lost to follow up, unlikely related to intervention |  |  |

**Table F5. Bias in the measurement of the outcome**

| Study | 4.1 Was the method of measuring the outcome inappropriate? | 4.2 Could measurement or ascertainment of the outcome have differed between intervention groups? | 4.3 If N/PN to 4.1 and 4.2: Were outcome assessors aware of the intervention received by the study participant? | 4.4 If Y/PY/NI to 4.3: Could assessment of the outcome have been influenced by knowledge of intervention received? | 4.5 If Y/PY/NI to 4.4: Is it likely that assessment of the outcome was influenced by knowledge of intervention received? | Risk of bias judgement |
| --- | --- | --- | --- | --- | --- | --- |
| Infuse studies | | | | | | |
| Boden 2000 [24] | No | No | Yes | Probably yes | Probably no | Some concerns |
|  | Outcomes have been measured objectively where possible and PROMS have been measured with standardised tools. | The same outcome measures were used across both groups | Trial was reported to be open label | For some PROMS outcomes |  |  |
| Burkus 2002 [26] | No | No | Yes | Probably yes | Probably no | Some concerns |
|  | Outcomes have been measured objectively where possible and PROMS have been measured with standardised tools. | The same outcome measures were used across both groups | Trial was reported to be open label | For some outcomes (ICBG pain) |  |  |
| Burkus 2002b [47] | No | No | Yes | Probably yes | Probably no | Some concerns |
|  | Outcomes have been measured objectively where possible and PROMS have been measured with standardised tools. | The same outcome measures were used across both groups | Trial was reported to be open label | For some PROMS outcomes |  |  |
| Gornet 2011 [29] | No | No | Yes | Probably yes | Probably no | Some concerns |
|  | Outcomes have been measured objectively where possible and PROMS have been measured with standardised tools. | The same outcome measures were used across both groups | Trial was reported to be open label | For some outcomes (e.g. pain) |  |  |
| **Studies of other interventions** | | | | | | |
| Chung 2003 [35] | No | No | No information | Probably no | Probably no | Low risk |
|  | Outcomes have been measured objectively where possible and PROMS have been measured with standardised tools. | The same outcome measures were used across both groups | Not reported | Intervention and comparator were different surgical methods |  |  |

**Table F6. Risk of bias in selection of the reported result and overall judgement**

| Study name | 5.1 Were the data that produced this result analysed in accordance with a pre-specified analysis plan that was finalized before unblinded outcome data were available for analysis? | 5.2. Is the numerical result being assessed likely to have been selected, on the basis of the results, from multiple eligible outcome measurements (e.g. scales, definitions, time points) within the outcome domain? | 5.3 Is the numerical result being assessed likely to have been selected, on the basis of the results, from multiple eligible analyses of the data? | Risk of bias judgement | RCT overall risk of bias |
| --- | --- | --- | --- | --- | --- |
| Infuse studies | | | | | |
| Boden 2000 [24] | No information | Probably no | No information | Some concerns | High risk of bias |
|  | Pre-specified analysis plan not available and study conducted before routine use of NCT database | It appears that all time points were reported and multiple outcomes were all reported | Analysis intentions are not available |  |  |
| Burkus 2002 [26] | No information | No | No information | Some concerns | High risk of bias |
|  | Pre-specified analysis plan not available and study conducted before routine use of NCT database | It appears that all time points were reported and multiple outcomes (for pain) were all reported. | Analysis intentions are not available |  |  |
| Burkus 2002b [47] | No information | Probably no | No information | Some concerns | High risk of bias |
|  | Pre-specified analysis plan not available and study conducted before routine use of NCT database | It appears that all time points were reported and multiple outcomes were all reported | Analysis intentions are not available |  |  |
| Gornet 2011 [29] | No information | No | No information | Some concerns | High risk of bias |
|  | Pre-specified analysis plan not available and study conducted before routine use of NCT database | It appears that all time points were reported and multiple outcomes (for pain) were all reported. | Analysis intentions are not available |  |  |
| Studies of other interventions | | | | | |
| Chung 2003 [35] | No information | No | No information | Some concerns | High risk of bias |
|  | Pre-specified analysis plan not available | It appears that all time points were reported and multiple outcomes (for pain) were all reported. | Analysis intentions are not available |  |  |

1. Risk of Bias Assessment of included Cohort Studies

**Table F7. JBI Cohort Studies Checklist Domains 1 to 6**

| Study ID | 1. Were the two groups similar and recruited from the same population? | 2. Were the exposures measured similarly to assign people to both exposed and unexposed groups? | 3. Was the exposure measured in a valid and reliable way? | 4. Were confounding factors identified? | 5. Were strategies to deal with confounding factors stated? | 6. Were the groups/participants free of the outcome at the start of the study (or at the moment of exposure)? |
| --- | --- | --- | --- | --- | --- | --- |
| Wang 2006 [33] | No | Yes | Unclear | No | NA | Probably yes |
|  | All patients with single-level lumbar DDD who underwent ALIF; however, groups are dissimilar in size and gender ratio. | Different types of surgery described | Graft material amount not reported | No confounders identified | NA | Any previous surgeries are not reported, but population had single-level DDD based on physical examination and radiographic findings |

**Table F8. JBI Cohort Studies Checklist Domains 7 to 11 and Overall Appraisal**

| Study | 7. Were the outcomes measured in a valid and reliable way? | 8. Was the follow up time reported and sufficient to be long enough for outcomes to occur? | 9. Was follow up complete, and if not, were the reasons to loss to follow up described and explored? | 10. Were strategies to address incomplete follow up utilized? | 11. Was appropriate statistical analysis used? | Overall appraisal |
| --- | --- | --- | --- | --- | --- | --- |
| Wang 2006 [33] | Unclear | No | Probably yes | NA | Yes | High risk |
|  | Definition of failed fusion is not reported | Duration of mean follow-up differed between groups, longest was 7.2 months | No withdrawals reported | NA | Descriptive statistics only |  |

1. Risk of Bias Assessment of Case series Studies

**Table F9. JBI Case Series Checklist Domains 1 to 5**

| Study | 1. Were there clear criteria for inclusion in the case series? | 2. Was the condition measured in a standard, reliable way for all participants included in the case series? | 3. Were valid methods used for identification of the condition for all participants included in the case series? | 4. Did the case series have consecutive inclusion of participants? | 5. Did the case series have complete inclusion of participants? |
| --- | --- | --- | --- | --- | --- |
| Infuse studies | | | | | |
| Burkus 2003 [8] | Yes | Unclear | Yes | Unclear | Unclear |
|  | Study included patients with DDD who had undergone ALIF | Not reported | Degenerative disc disease as noted by back pain of discogenic origin, with or without leg pain, with degeneration of the disc confirmed by patient history ( e.g. pain [leg, back, or symptoms in the sciatic nerve distribution], function deficit and/or neurological deficit) and radiographic studies ( e.g., CT, MRl, X-Ray, etc.) to include one or more of the following: instability (defined as angular motion > 5° and/or translation >= 4mm, based on Flex/Ext radiographs); osteophyte formation; decreased disc height; thickening of ligamentous tissue; disc degeneration or herniation; and/or facet joint degeneration. | Not reported | Not reported |
| Chatha 2014 [28] | Yes | Unclear | Unclear | Unclear | Unclear |
|  | Study included patients with DDD who had undergone operative repair | Not reported | Diagnostic criteria not reported | Study says that included patients were from a single operator, but not reported whether all (consecutive) patients were included | Does not report whether the included patients comprised all who were available |
| Kalb 2016 [30] | Yes | Unclear | Unclear | Yes | No |
|  | Study included patients who underwent single or multi-level ALIF | Not reported | Diagnostic criteria not reported | Consecutive patients included | Of the 242 patients who underwent ALIF, 231 were included |
| Kleeman 2001 [31] | Yes | Yes | Yes | Unclear | No |
|  | Study included patients with refractory low back pain with or without leg pain from single level degenerative disc disease or low-grade spondylolisthesis | Surgical pathology was verified with radiograph, MRI, and/or discogram | Surgical pathology was verified with radiograph, MRI, and/or discogram | Study says that included patients were from a single operator, but not reported whether all (consecutive) patients were included | One patient transferred care elsewhere before the 3-month follow-up and was unavailable for further evaluation |
| Malham 2014 [32] | Yes | Unclear | Unclear | Yes | Probably |
|  | Patients who underwent ALIF for severe discogenic pain, radiculopathy, and Grade 1 and 2 degenerative and isthmic spondylolistheses | Not reported | Diagnostic criteria not reported | Consecutive patients included | Described as consecutive patients and no withdrawals reported |
| Studies of other interventions | | | | | |
| Abbasi 2015 [34] | Yes | Yes | Unclear | Yes | Unclear |
|  | Patients who underwent OLIF for DDD, disc herniation, listhesis, stenosis or scoliosis | Pre-operative imaging included MRI, x-ray of the lumbar spine with flexion and extension and in many cases, a discogram and CT scan. | Diagnostic criteria not reported | Patients reported to be consecutive | Withdrawals not reported |
| Choi 2005 [36] | Yes | Yes | Unclear | Unclear | Probably |
|  | Patients who underwent a mini open laparotomy followed by the ALIF procedure to treat recurrent lumbar disc herniation, inclusion criterion for surgery was intractable radicular pain combined with back pain of >8 weeks’ duration refractory to conservative treatment | Postoperative radiographs, including dynamic flexion–extension views, were obtained at regular intervals to assess fusion progression | Diagnostic criteria not reported in detail | Not reported whether all (consecutive) patients were included | No withdrawals reported |
| Choi 2006 [37] | Yes | Yes | Unclear | Unclear | Probably |
|  | Patients who underwent a single-level ALIF using paired stand-alone rectangular cages | Radiologic evaluation was performed using standing AP, lateral plain radiograph, and dynamic flexion extension films in lateral decubitus to determine the fusion. | Diagnostic criteria not reported in detail | Not reported whether all (consecutive) patients were included | No withdrawals reported |
| Hironaka 2013 [38] | Yes | Yes | Yes | Yes | Probably |
|  | Patients who underwent single level ALIF for lumbar spinal degenerative disorder | Preoperative imaging including magnetic resonance imaging (MRI), dynamic radiography, myelography, myelographic computed tomography (CT), and three-dimensional CT angiography | Diagnostic criteria clearly reported. | Study reports that all 142 patients who underwent ALIF were followed up at 2 years. | No withdrawals reported |
| Lee 2017 [39] | Yes | Yes | Unclear | Yes | Probably |
|  | Patients with degenerative spinal stenosis and instability who underwent fusion on a single level, L5-S1. All patients had severe lower back pain as a chief complaint, and leg pain or neurogenic intermittent claudication collaterally. ALIF was performed in patients who complained primarily of lower back pain, rather than leg pain or neurogenic intermittent claudication. In addition, they showed severe disk space narrowing and a moderate grade of spondylolytic spondylolisthesis. | Condition assessed using CT imaging | Diagnostic criteria not reported in detail | Patients reported to be consecutive | No withdrawals reported |
| Pinson 2017 [40] | Yes | Yes | Yes | Unclear | No |
|  | Patients with degenerative disc disease (DDD). | Use of MRI imaging and clinical examination | The indication for surgical intervention was made by the individual surgeon. The decision was based on a correlation between clinical parameters such as lumbosciatica, loss of spinal reflexes, motor weakness, sensory loss, and paresthesias on one hand and the presence of the different components of degenerative disc disease (DDD), such as decrease in disc height, presence of disc bulging or herniation, osteophytosis, and Schmorl nodules on spinal imaging on the other hand. | Not reported whether all (consecutive) patients were included | At baseline, the cohort included 166 patients with 200 fusion levels. Patients who could not be contacted by telephone or who refused cooperation were excluded (n=13), and patients whose files for which preoperative imaging and/or 3-year follow-up parameters could not be retrieved (n=30) were excluded |
| Sarwat 2001 [41] | Yes | Yes | Unclear | Yes | Probably |
|  | Patients suffering from intractable low back pain with or without sciatica who underwent anterior lumbar intervertebral fusion using composite graft made of femoral cortical allograft rings packed with cancellous allograft bone chips | All patients were investigated preoperatively with MRI. Provocative discography was performed in all cases where the MRI showed more than one-segment pathology. Correlation between the clinical findings, MRI findings, and discography was essential if operative treatment was to be offered to the patients. | All patients were investigated preoperatively with MRI. Provocative discography was performed in all cases where the MRI showed more than one-segment pathology. Correlation between the clinical findings, MRI findings, and discography was essential if operative treatment was to be offered to the patients. Further details on criteria not reported. | Patients reported to be consecutive | No withdrawals reported |
| Strube 2012 [42] | Yes | Yes | Yes | Unclear | No |
|  | Patients who presented persistent lumbosacral, and/or radicular complaints after an unsuccessful conservative therapy. | Fusion assessed by radiograph and CT scan. | For both groups, patients who presented persistent lumbosacral, and/or radicular complaints after an unsuccessful conservative therapy, covering a period of at least 6 months, were enrolled in this study. At the same time, at least 1 of the following had to be detectable in magnetic resonance imaging (MRI): osteochondrosis in Modic stage Z2 with a residual disc height of <7 mm, resulting from idiopathic intervertebral disc degeneration; a facet joint degeneration, Fujiwara stage Z3; a neuroforaminal stenosis resulting from the loss of disc height in segments L4/5 or L5/S1 (L6/S1 in cases with 6 free lumbar vertebrae) | Not reported whether all (consecutive) patients were included | 6 patients in the ALIF group were lost to follow up |
| Xu 2023 [43] | Yes | Yes | Unclear | Yes | No |
|  | Patients who underwent OLIF by a single surgeon | Clinical outcome was assessed by multiple questionnaires, including Oswestry disability index (ODI), Japanese Orthopaedic Association (JOA) score rating system, short form-36 health survey (SF-36), and visual analog scale (VAS) for low back pain. Radiographic outcome was evaluated by measuring the parameters such as disc height, lumbar lordosis, and segmental angle on the standard standing lateral radiographs, and the space angle of the fusion level on the dynamic views of the lateral radiographs. | Diagnostic criteria not reported in detail | Patients described as consecutive | Eight cases were excluded due to the application of this combined procedure in the L5/S1 level, in the patients with pyogenic discitis or with a vertebral fracture, or just lost to follow-up |

**Table F10. JBI Case Series Study Checklist Domains 6 to 10 and Overall Appraisal**

| Study ID | 6. Was there clear reporting of the demographics of the participants in the study? | 7. Was there clear reporting of clinical information of the participants? | 8. Were the outcomes or follow up results of cases clearly reported? | 9. Was there clear reporting of the presenting site(s)/clinic(s) demographic information? | 10. Was statistical analysis appropriate? | Overall appraisal |
| --- | --- | --- | --- | --- | --- | --- |
| Infuse studies | | | | | | |
| Burkus 2003 [8] | Yes | Yes | Yes | Yes | Yes | Low risk of bias |
|  | Relevant demographic information reported | Clinical information well reported | Outcomes were mostly well reported. | Details about the site were reported | Appropriate analyses |  |
| Chatha 2014 [28] | Unclear | No | No | No | No | High risk of bias |
|  | Some demographic information was reported, but other important information (weight, ethnicity, prior back surgery) was not reported | Data was presented in graphs, however many points overlapped making the graphs difficult to read. There were no numerical data reported. | Outcomes were sporadically reported in the text and graphs were not sufficient | No details about the clinical site | No measures of variance were reported. Several comments were made about results being significant, but no data to confirm this |  |
| Kalb 2016 [30] | Yes | Yes | Yes | Yes | Yes | Low risk of bias |
|  | Demographic characteristics were well reported | Clinical information well reported | Outcomes were mostly well reported. | Details about the site were reported | Appropriate analyses |  |
| Kleeman 2001 [31] | Yes | Yes | Yes | No | Yes | Low risk of bias |
|  | Demographic characteristics were well reported | Clinical information well reported | Outcomes were well reported | No details about the clinical site | Appropriate analyses |  |
| Malham 2014 [32] | Yes | Yes | Yes | No | Yes | Low risk of bias |
|  | Demographic characteristics were well reported | Clinical information well reported | Outcomes were well reported | No details about the clinical site | Appropriate analyses |  |
| Studies of other interventions | | | | | | |
| Abbasi 2015 [34] | No | No | Unclear | Yes | No | High risk of bias |
|  | Only age and BMI reported | No clinical information reported | Study reports that patients were followed up at 3, 6, 9 and 12 months, but only peri-operative data reported | Surgeries undertaken at 2 hospitals, locations reported | No description of statistical analyses undertaken |  |
| Choi 2005 [36] | No | Yes | Yes | Yes | Yes | Low risk of bias |
|  | Some demographic information was reported, but other important information (weight, smoking status etc.) was not reported | Clinical information well reported | Outcomes were well reported | Site details and location reported in author affiliations | Appropriate analyses |  |
| Choi 2006 [37] | No | Yes | Yes | Yes | Yes | Low risk of bias |
|  | Some demographic information was reported, but other important information was not reported | Clinical information well reported | Outcomes were well reported | Site details and location reported in author affiliations | Appropriate analyses |  |
| Hironaka 2013 [38] | No | Yes | Yes | Yes | Yes | Low risk of bias |
|  | Some demographic information was reported, but other important information (weight, smoking status etc.) was not reported | Clinical information well reported | Outcomes were well reported | Details about the sites were reported | Appropriate analyses |  |
| Lee 2017 [39] | Unclear | Yes | Yes | Yes | Yes | Low risk of bias |
|  | Demographic information well reported overall, but not well for each surgical approach | Clinical information well reported | Outcomes were well reported | Site location details reported in author affiliations | Appropriate analyses |  |
| Pinson 2017 [40] | Unclear | No | No | Yes | Yes | Some concerns |
|  | Only some demographic information reported | Clinical information not well reported, | Outcomes were not well reported. There was no clear reporting of fusion outcomes, data needed to be digitized from graphs and other outcomes were not reported clearly enough to extract | Site location details reported in author affiliations | Appropriate analyses |  |
| Sarwat 2001 [41] | Unclear | Unclear | Yes | No | Yes | Some concerns |
|  | Age and gender reported for single level patients, no other details reported (e.g. previous surgery, smoking status, weight etc.) | Limited clinical information reported | Outcomes were well reported | Reported that cases were from a single surgeon, author affiliations would suggest a single centre but this is not reported explicitly. | Appropriate analyses |  |
| Strube 2012 [42] | Yes | Yes | Yes | No | Yes | Low risk of bias |
|  | Demographic information was well reported | Clinical information was well reported | Outcomes were clearly reported | Details of the clinical site not reported | Appropriate analyses |  |
| Xu 2023 [43] | Yes | Yes | Yes | Yes | Yes | Low risk of bias |
|  | Demographic information was well reported | Clinical information was well reported | Outcomes were clearly reported | Site location details reported in author affiliations | Appropriate analyses |  |

# Appendix G: Detailed Data for Case Series Studies

**Table H1. Fusion outcomes; Infuse studies**

| **Study ID** | **Study design** | **How fusion status was evaluated** | **Criteria for fusion success** | **Details about who measured fusion success** | **Intervention** | **Timepoint of assessment** | **Number of fusions**  **n (%)** | **p-value for difference between study arms** |
| --- | --- | --- | --- | --- | --- | --- | --- | --- |
| Burkus, 2003 [8] | Retrospective case series | Radiographic and computed tomographic scan findings. | Fusion was defined as bridging bone connecting the adjacent vertebral bodies either through the implants or around the implants, <5 of angular motion, ≤3mm of translation, and an absence of radiolucent lines around >50% of either implant. | Two independent, blinded radiologists interpreted all radiographs and CT scans. A third independent, blinded radiologist was used to adjudicate conflicting fusion findings. | Infuse | 6 months | 88/134 (92.6) | NA |
|  |  |  |  |  |  | 12 months | 95/134 (94.1) | NA |
|  |  |  |  |  |  | 24 months | 81/134 (94.2) | NA |
| Kalb, 2016 [30] | Retrospective case series | Fusion was assessed using postoperative radiography. | Successful fusion criteria included the following:  (1) bone fusion was more dense and more mature than it appeared during surgery  (2) no interspace was present between the cage and vertebral body  (3) mature bony trabeculae | Number of assessors, certification, blinding, independence, author status was not reported. | Infuse | Mean: 11 months (Range 1-75) | 229/231 (99) | NA |
| Kleeman, 2001 [31] | Prospective case series | Radiographs were repeated after surgery at 6 and 12 months. CT scans with sagittal and coronal reconstruction were performed at the same intervals. | Fusion was defined by evidence of bridging trabeculae on radiographs or CT in at least one of the following areas: lateral, medial, anterior, posterior, and/or through either one or both of the implants.  Other criteria included no more than 3 mm difference in translation or 5° difference in angulation on flexion–extension radiographs and no evidence of radiolucency surrounding 50% of either device on radiographs. All of the criteria had to be met to be classified as fused. | An unbiased radiologist read postoperative computed tomography scans for evidence of fusion. An independent neuroradiologist reviewed all CT scans. | Infuse | 6 months | 21/21 (100) | NA |
|  |  |  |  |  |  | 12 months | 21/21 (100) | NA |
| Malham, 2014 [32] | Prospective case series | Lumbar CT scans were obtained using a high-definition (HD) CT scanner (Somatom Definition Flash, Siemens AG) 2 days postoperatively to assess instrumentation and then at 6,9, and 12 months until solid interbody fusion was confirmed on coronal and sagittal views. | Fusion was assessed based on lumbar CT scans 2 days postoperatively to assess instrumentation and then at 6,9, and 12 months until solid interbody fusion was confirmed on coronal and sagittal views. Fusion was defined as the presence of bridging interbody trabecular bone. | Lumbar CT scans were obtained by an independent radiologist, no further details reported. | Infuse | 6 months | 62/86 (72.1) | NA |
|  |  |  |  |  |  | 9 months | 81/86 (94.2) | NA |
|  |  |  |  |  |  | 12 months | 83/86 (96.5) | NA |

Abbreviations: ALIF, anterior lumbar interbody fusion; CT, computed tomography; HD, high definition; ICBG, iliac crest bone graft; MRI, magnetic resonance imaging; NA, not applicable; NR, not reported; RCT, randomised controlled trial; ROM, range of motion

**Table H2. Fusion outcomes; Other interventions**

| Study ID | Study design | How fusion status was evaluated | Criteria for fusion success | Details about who measured fusion success | Intervention | Timepoint of assessment | Number of fusions  n (%) | p-value |
| --- | --- | --- | --- | --- | --- | --- | --- | --- |
|  |  |  |  |  | Autologous graft, Mini-open ALIF | Mean: 30 months (Range 24-40) | 20 /22 (91) |  |
| Choi, 2005 [36] | Retrospective case series | Postoperative radiographs, including dynamic flexion–extension views, were obtained at regular intervals to assess fusion progression. In some cases, postoperative CT and MRI were obtained to supplement the radiographic findings. | A fusion was confirmed by a progressive increase in interspace bone density and blurring of adjacent endplates, presence of bridging bone in the interbody space, and no evidence of loosening or motion on flexion lateral radiographs. | The follow-up radiographs were evaluated by an independent examiner. | Allograft bone | 35 months (SD 8.9, range 30–42) | 22/22 (100) | NA |
| Choi, 2006 [37] | Retrospective case series | Radiologic evaluation was performed using standing AP, lateral plain radiograph, and dynamic flexion/extension films in lateral decubitus to determine the fusion. | Solid fusion was defined as grades 1 or 2 fusion - grade I, fused with remodelling and trabeculae; grade II, graft intact, not fully remodelled and incorporated though but with no lucencies above or below. | One independent investigator analysed the radiographic results. No further details reported. | Allograft bone | Mean 27 months (Range 19-38) | 78/90 (86.7) | NA |
| Hironaka Y, 2013 [38] | Retrospective case series | Radiographic assessments were made, including MRI, dynamic radiography, myelography, myelographic CT, and three-dimensional CT angiography. | Radiological identification of lumbar arthrodesis was defined as follows: the absence of lucency around the threaded interbody implant, the presence of bridging bone incorporating the anterior bone graft, and the absence of movement > 3 mm on dynamic X-rays. | Radiographic assessments were made by an independent observer. | Autogenous vertebral spur and apacerum powder | Mean: 76 months (range 26–146.6) | 128/142 (90.1) | NA |
| Lee N, 2017 [39] | Retrospective case series | To evaluate the fusion rate, a postoperative computed tomography (CT) scan was taken 1 year after surgery. If complete fusion was achieved with confirmation on CT scan 1 year after surgery, no more CT scans were taken. However, if the CT scan showed incomplete fusion 1 year after surgery, a CT scan was taken again at 2 years after surgery. | Complete fusion was defined as trabecular continuity of grafted bone material without any visual gap on CT scan (grade 1). In addition, segmental ROM was used as another tool to assess fusion state. When segmental ROM was greater than 4 degrees, it was regarded as nonfused; when it was below 4 degrees, it was regarded as fused.  If complete fusion was achieved with confirmation on CT scan 1 year after surgery, no more CT scans were taken. However, if the CT scan showed incomplete fusion 1 year after surgery, a CT scan was taken again at 2 years after surgery. | No details on who assessed the images or whether they were independent. Two senior spinal neurosurgeons performed all operations in this study. | Allograft demineralised bone matrix | 1 year | 16/26 (61.5) | NA |
|  |  |  |  |  |  | 2 years | 9/13 (69.2) | NA |
| Sarwat, 2001 [41] | Retrospective case series | Lateral tomography was exclusively to assess the graft-host interface, and hence, the fusion. | Radiological fusion was diagnosed when the lucent line at the entire host-endplate interface had disappeared on lateral tomography. | All radiological investigations were reviewed and reported on by a single orthopaedic radiologist. | Allograft bone | ≤3 months | 11/24 (45.8) | NA |
|  |  |  |  |  |  | 6 months | 19/24 (79.2) | NA |
|  |  |  |  |  |  | 9 months | 21/24 (87.5) | NA |
|  |  |  |  |  |  | 1 year | 23/24 (95.8) | NA |
|  |  |  |  |  |  | Average 24.4 months | 24/24 (100) | NA |
| Strube, 2012 [42] | Prospective case series | The qualitative radiographic evaluation of fusion was based on the criteria for vertebral body fusion using intersomatic cages suggested by McAfee et al and Ray, and was performed at 3, 6, and 12 months and at final follow-up. This was on the basis of both plain and extension-flexion radiographs.  In addition, qualitative fusion was determined by thin-layer CT scans (layer thickness - 1 mm) using sagittal and coronary plane reconstructions at 12-month follow-up. | Radiograph: A positive evaluation required that each of the following criteria be fulfilled: lack of any visible motion using Hutter method, or<2 degrees of intersegmental change, as seen on flexion and extension radiographs by the Simmons method; lack of a dark halo around the implant material; lack of disc space height loss of more than 1 mm indicating a resistance to collapse of the cancellous vertebral bone; lack of visible fracture of the device, graft, or vertebrae; lack of substantial sclerotic changes in the recipient bone bed or the graft; visible bridging bone around the PEEK fusion cage as seen on anteroposterior or lateral radiographs.  CT: According to the protocol of Williams et al, fusion was defined by fulfilling each of the following criteria: lack of any lucency at the implant material margins; lack of any visible fracture of the device, graft, or vertebrae; lack of any cystic changes within the endplates adjacent to the implant; lack of any linear defects (fracture) through intervertebral new bone within, or adjacent to, the titanium cage parallel to the endplates; lack of a high subsidence level of the cages or dislocation; bridging bone external to the/in the cage. | The radiographs and CT reconstructions were evaluated, independently and blinded, by both a radiologist specialized in spinal imaging and an orthopaedic surgeon. A second independent orthopaedic surgeon was used to adjudicate on conflicting fusion findings. | Allograft bone | 6 months | 7/34 (20.6) | NA |
|  |  |  |  |  |  | 1 year | 28/34 (82.4) | NA |
|  |  |  |  |  |  | Mean 41 months | 31/34 (91.2) | NA |
| Xu, 2023 [43] | Retrospective case series | Mainly (author reported) evaluated on computed tomography scan images 12 and 24 months postoperatively. | Complete fusion was defined as the presence of continuous bridging trabecular bone connecting the adjacent vertebral bodies through or around the implants. | Results were determined by a spine surgeon who was blinded to the patient information. Measurement of radiological parameters was blinded from the clinical results. | Allograft bone | 3 months | 5/34 (14.7) | NA |
|  |  |  |  |  |  | 6 months | 15/34 (44.1) | NA |
|  |  |  |  |  |  | 1 year | 31/34 (91.2) | NA |
|  |  |  |  |  |  | 2 years | 31/34 (91.2) | NA |

Abbreviations: ALIF, anterior lumbar interbody fusion; CT, computed tomography; MRI, magnetic resonance imaging; NA, not applicable; NR, not reported; RCT, randomised controlled trial; ROM, range of motion

**Table H3. Operative time; Infuse studies**

| Study ID | Study design | Measure of operative time | Intervention | n | Mean (SD) | p-value for difference |
| --- | --- | --- | --- | --- | --- | --- |
| Burkus 2003 [8] | Prospective case series | Hours | Infuse | 134 | Mean 1.9 (SD 0.9) | NA |
| Kleeman 2001 [31] | Prospective case series | min | Infuse | 21 | Mean 102 (Range 65-190) | NA |
| Wang 2006 [33] | Retrospective cohort | min | Infuse + SPIRE plate | 21 | Median 164 (Range 123-217) | NA |
|  |  |  | Infuse + open BPS | 3 | Median 239 (Range 207-240) |  |
|  |  |  | Infuse + MAST BPS | 8 | Median 250 (Range 159-299) |  |

Abbreviations: ALIF, anterior lumbar interbody fusion; BPS, bilateral pedicle screw; ICBG, Iliac crest bone graft; MAST, minimum access spinal technology; NA, not applicable; NR, not reported; RCT, randomised controlled trial

**Table H4. Operative time; Other interventions**

| Study ID | Study design | Measure of operative time | Intervention | n | Operative time | p-value for difference |
| --- | --- | --- | --- | --- | --- | --- |
| Abbasi 2015 [34] | Retrospective case series | min | Tricalcium phosphate soaked in autologous bone marrow aspirate | 28 | Mean 69.2 (SD 29.4) | NA |
| Choi 2005 [36] | Retrospective case series | min | Allograft bone | 22 | Mean 76.5 (Range 50-110) | NA |
| Hironaka 2013 [38] | Retrospective case series | min | Autogenous vertebral spur and apacerum powder | 142 | Mean 155.5 (Range 96–280) | NA |
| Lee 2017 [39] | Retrospective case series | min | Allograft demineralised bone matrix | 26 | Mean 159.35 (SD 45.81) | NA |
| Strube 2012 [42] | Prospective case series | min | Allograft bone | 34 | Mean 95.6 (Range: 60-205) | NA |

Abbreviations: ALIF, anterior lumbar interbody fusion; CT, computed tomography; HD, high definition; ICBG, iliac crest bone graft; MRI, magnetic resonance imaging; NA, not applicable; NR, not reported; RCT, randomised controlled trial; ROM, range of motion

Notes: Blue text indicates YHEC calculated values; Yellow highlight indicates trials that could be considered for statistical pooling

**Table H5. Blood loss; Infuse studies**

| **Study ID** | **Study design** | **Blood loss measure** | **Intervention** | **n** | **Blood loss** | **p-value for difference** |
| --- | --- | --- | --- | --- | --- | --- |
| **Observational studies** | | | | | | |
| Burkus 2003 [8] | Prospective case series | ml | Infuse | 134 | Mean 146.1 (406.2) | NA |
| Kleeman 2001 [31] | Prospective case series | NR | Infuse | 21 | Mean 33 (SD NR) | NA |
| Malham 2014 [32] | Prospective case series | ml | Infuse | 84 | Mean 115 (SD 151) | NA |
| Wang 2006 [33] | Retrospective cohort | ml | Infuse + SPIRE plate | 21 | Median 75 (Range 50-200) | NA |
|  |  |  | Infuse + open BPS | 3 | Median 150 (Range 100-350) | NA |
|  |  |  | Infuse + MAST BPS | 8 | Median 125 (Range 50-250) | NA |

Abbreviations**:** ALIF, anterior lumbar interbody fusion; BPS, bilateral pedicle screw; ICBG, iliac crest bone graft; ITT, intention to treat; MAST, minimum access spinal technology; ml, millilitres; n, number of participants; NA, not applicable; NR, not reported; NS, not significant; PP, per protocol; RCT, randomised controlled trial; SD, standard deviation

Notes**:** Yellow highlight indicates trials that could be considered for statistical pooling

**Table H6. Blood loss; Other interventions**

| Study ID | Study design | Blood loss measure | Intervention | n | Blood loss | p-value for difference |
| --- | --- | --- | --- | --- | --- | --- |
| Abbasi 2015 [34] | Retrospective case series | ml | Tricalcium phosphate soaked in autologous bone marrow aspirate | 28 | Mean 29.4 (SD 17.2) | NA |
| Choi 2005 [36] | Retrospective case series | ml | Allograft bone | 22 | Mean 157 (Range 50-600) | NA |
| Hironaka 2013 [38] | Retrospective case series | ml | Autogenous vertebral spur and apacerum powder | 142 | Mean 63.7 (Range 10-456) | NA |
| Lee 2017 [39] | Retrospective case series | cm3 | Allograft bone | 26 | Mean 461.54 (SD 465.89) | NA |
| Strube 2012 [42] | Prospective case series | mL | Allograft bone | 34 | Mean 90.3 (Range 20-600) | NA |

Abbreviations: ALIF, anterior lumbar interbody fusion; ICBG, iliac crest bone graft; n, number of participants; NA, not applicable; NS, not significant; PP, per protocol; RCT, randomised controlled trial; SD, standard deviation

**Table H7. Length of stay; Infuse studies**

| Study ID | Study design | Intervention | n | Mean days (SD) | p-value for difference |
| --- | --- | --- | --- | --- | --- |
| Burkus 2003 [8] | Prospective case series | Infuse | 134 | Mean 1.2 (SD 1.1) | NR |
| Wang 2006 [33] | Retrospective cohort | Infuse + SPIRE plate | 21 | Median 3 (Range 2-4) | NR |
|  |  | Infuse + open BPS | 3 | Median 4 (Range 3-4) | NR |
|  |  | Infuse + MAST BPS | 8 | Median 3 (Range 2-5) | NR |
| Kleeman 2001 [31] | Prospective case series | Infuse | 21 | Mean 1.0 (SD NR) | NR |

Abbreviations**:** ALIF, anterior lumbar interbody fusion; ICBG, Iliac crest bone graft; ITT, Intention to treat; n, Number of participants; NA, Not applicable; NR, Not reported; NS, Not significant; PP, Per protocol; RCT, Randomised controlled trial; SD, Standard deviation

Notes: Yellow highlight indicates trials that could be considered for statistical pooling

**Table H8. Length of stay; Other interventions**

| Study ID | Study design | Intervention | n | Mean | p-value for difference |
| --- | --- | --- | --- | --- | --- |
| Abbasi 2015 [34] | Retrospective case series | Tricalcium phosphate soaked in autologous bone marrow aspirate | 28 | Mean 2.6 (SD 1.7) | NA |
| Choi 2005 [36] | Retrospective case series | Allograft bone | 22 | Mean 5.5 (Range 4-10) | NA |
| Hironaka 2013 [38] | Retrospective case series | Autogenous vertebral spur and apacerum powder | 142 | Mean 6.9 (Range 3-21) | NA |
| Lee 2017 [39] | Retrospective case series | Allograft demineralised bone matrix | 26 | Mean 6.44 (SD 1.58) | NA |
| Sarwat 2001 [41] | Retrospective case series | Allograft bone | 24 | Mean 4.0 (Range 3-12) | NA |

Abbreviations: ALIF, anterior lumbar interbody fusion; ICBG, iliac crest bone graft; n, number of participants; NA, not applicable; NR, not reported; NS, not significant; PP, per protocol; RCT, randomised controlled trial; SD, standard deviation

**Table H9. ODI; Infuse studies**

| Study ID | Study design | Timepoint of assessment | Intervention | n | ODI measures | | | Change from baseline | | |  | |
| --- | --- | --- | --- | --- | --- | --- | --- | --- | --- | --- | --- | --- |
|  |  |  |  |  | Mean (SD) | p-value for differences between baseline and follow up | p-value for difference between treatment arms | Mean (SD) | p value for differences between baseline and follow up | p-value for difference between treatment arms | Improvement in ODI n (%) | p-value for difference |
| Burkus 2003 Baseline to 24 months [8]  Burkus 2009 4 and 6 years [7] | Prospective case series | Baseline | Infuse (lap) | 134 | 52.3 (12.7) | NR | NR | NR | NR | NR | NR | NR |
|  |  | 3 months | Infuse (lap) | 127 | 30.2 (19.9) | NR | NR | NR | NR | NR | NR | NR |
|  |  | 6 months | Infuse (lap) | 120 | 25.1 (20.4) | NR | NR | NR | NR | NR | NR | NR |
|  |  | 12 months | Infuse (lap) | 114 | 20.4 (19.8) | NR | NR | NR | NR | NR | NR | NR |
|  |  | 24 months | Infuse (lap) | 93 | 18.7 (19.3) | NR | NR | NR | NR | NR | NR | NR |
|  |  | 4 years | Infuse (lap) | 55 | 14.3 (18.2) | NR | NR | NR | NR | NR | NR | NR |
|  |  | 6 years | Infuse (lap) | 68 | 15.5 (18.9) | NR | NR | NR | NR | NR | NR | NR |
| Chatha 2014 [28] | Prospective case series | 3 months | Infuse | 196 | NR (NR) | NR | NR | 36.8% improvement | Significant difference (not further defined) | NR | NR | NA |
| Kleeman 2001 [31] | Prospective case series | Baseline | Infuse | 21 | 47 (8.7) | NR | NR | NR | NR | NR | NR | NA |
|  |  | 6 months | Infuse | 21 | 16 (12) | NR | NR | NR | NR | NR | 18/21 (86)^2^ | NA |
|  |  | 12 months | Infuse | 21 | 11 (10) | p<0.001 | NR | NR | NR | NR | 21/21 (100) | NA |
| Malham 2014 [32] | Prospective case series | Pre-operative | Infuse | 86 | 49.7 (16) | NR | NR | NR | NR | NR | NR | NA |
|  |  | Last follow up (6-24 months) | Infuse | 86 | 23.1 (19.7) | NR | NR | NR | NR | NR | NR | NA |

Abbreviations: ADR: Artificial disc replacement; ICBG, iliac crest bone graft; ITT, intention to treat; Lap, laparoscopy; n, number of participants; NA, not applicable; NR, not reported; NS, not significant; ODI, Oswestry Disability Index; PP, per protocol; RCT, randomised controlled trial; SD, standard deviation

**Table H10. ODI; Other interventions**

| Study ID | Study design | Timepoint of assessment | Intervention | n | ODI measures | | | Change from baseline | | | Improvement in ODI  n (%) |
| --- | --- | --- | --- | --- | --- | --- | --- | --- | --- | --- | --- |
|  |  |  |  |  | Mean (SD) | p-value for differences between baseline and follow up | p-value for difference between treatment arms | Mean (SD) | p value for differences between baseline and follow up | p-value for difference between treatment arms |  |
| Pinson, 2017 [40] | Retrospective case series | 3 years | Allograft bone | 123 | NR | NR | NR | NR | NR | NR | Improved 2 ODI categories vs baseline: 95/123 (77.2) |
|  |  |  |  |  | NR | NR | NR | NR | NR | NR | Improved 1 ODI category vs baseline: 21/123 (17.1) |
|  |  |  |  |  | NR | NR | NR | NR | NR | NR | No ODI change: 6/123 (4.9) |
|  |  |  |  |  | NR | NR | NR | NR | NR | NR | ODI deterioration: 1/123 (0.8) |
| Strube 2012 [42] | Prospective case series | Pre-operative | Allograft bone | 34 | 62.9 (14.8) | NR | NR | NR | NR | NR | NA |
|  |  | 3 months | Allograft bone | 34 | 35.7 (12.1) | NR | NR | NR | NR | NR | NA |
|  |  | 6 months | Allograft bone | 34 | 31.3 (13.1) | NR | NR | NR | NR | NR | NA |
|  |  | 1 year | Allograft bone | 34 | 25.8 (11.7) | NR | NR | NR | NR | NR | NA |
|  |  | 2 years | Allograft bone | 34 | 24.1 (11.8) | NR | NR | NR | NR | NR | NA |
|  |  | Mean 41 months | Allograft bone | 34 | 22.8 (10.2) | NR | NR | NR | NR | NR | NA |
| Xu 2024 [43] | Retrospective case series | Baseline | Allograft bone | 34 | 54.6 (9.8) | NR | NR | NR | NR | NR | NA |
|  |  | 3 months | Allograft bone | 34 | 20.2 (7.7) | p<0.001 | NR | NR | NR | NR | NA |
|  |  | 6 months | Allograft bone | 34 | 14.1 (6) | p<0.001 | NR | NR | NR | NR | NA |
|  |  | 1 year | Allograft bone | 34 | 10.1 (4) | p<0.001 | NR | NR | NR | NR | NA |
|  |  | 2 years | Allograft bone | 34 | 9.2 (3.3) | p<0.001 | NR | NR | NR | NR | NA |

**Table H11. Complications; Infuse studies**

| Study ID | Study design | Timepoint of assessment | Intervention | Type of intra-operative complication | Number (%) of intraoperative events | p-value for difference between study arms |
| --- | --- | --- | --- | --- | --- | --- |
| Wang 2006 [33] | Retro-spective cohort | Mean 5.5 months (Range 1.0-12.0) | Infuse + SPIRE plate | NA | 0/21 (0) | NR |
|  |  | Mean 7.2 months (Range 6.5-12.6) | Infuse + open BPS | NA | 0/3 (0) | NR |
|  |  | Mean 4.9 months (Range 1.0-13.4) | Infuse + MAST BPS | NA | 0/8 (0) | NR |
| Chatha 2014 [28] | Prospective case series | NA | Infuse | Lacerations to the veins | 9/286 (3.1) | NR |
| Kalb 2016 [30] | Retro-spective case series | Mean: 11 months (Range 1-75) | Infuse | Durotomy: 2  Great vessel lesion: 1  Bowel laceration: 1 | 4/231 (1.8) | NR |
| Kleeman 2001 [31] | Prospective case series | Peri-operative | Infuse | Bowel injury: 1  Vascular injury: 1 | 2/21 (9.0) | NR |

Abbreviations: ALIF, anterior lumbar interbody fusion; BPS, bilateral pedicle screw; ICBG, iliac crest bone graft; MAST, minimum access spinal technology; NA, not applicable; NR, not reported; RCT, randomised controlled trial

**Table H12. Complications; Other interventions**

| Study ID | Study design | Timepoint of assessment | Intervention | Number (%) of intraoperative events | Type of intra-operative complication | p-value for difference between study arms |
| --- | --- | --- | --- | --- | --- | --- |
| Choi 2005 [36] | Retrospective case series | 35 months (SD 8.9, range 30–42) | Allograft bone | 1/22 (4.5) | Iliolumbar vein tear: 1 | NR |
| Hironaka 2013 [38] | Retrospective case series | Mean: 76 months (range 26–146.6) | Autogenous vertebral spur and apacerum powder | 1/142 (0.7) | Liquorrhea due to a dural tear injury: 1 | NR |
| Lee 2017 [39] | Retrospective case series | Mean 23.81 (17.37) | Allograft demineralised bone matrix | 1/26 (3.8) | Iliac vein injury: 1 | NR |
| Strube 2012 [42] | Prospective case series | Mean 41 months | Allograft bone | 2/34 (5.8) | Iliac vein injury: 1  Sympathetic nervous system injury: 1 | NR |

Abbreviations: ALIF, anterior lumbar interbody fusion; DVT, deep vein thrombosis; ICBG, iliac crest bone graft; NA, not applicable; NR, not reported; PP, per protocol; RCT, randomised controlled trial

**Table H13. Long term complications; Infuse studies**

| Study ID | Study design | Timepoint of assessment | Complication description | Intervention | Number (%) of long-term complications | p-value for difference between study arms |
| --- | --- | --- | --- | --- | --- | --- |
| Kalb 2016 [30] | Retro-spective case series | Mean: 11 months (Range 1-75) | Post-operative complications | Infuse | 28/231 (12.0) | NA |
| Malham 2014 [32] | Pro-spective case series | Median 12 months (Range 6 to 24) | Major complications^1^ | Infuse | 5/86 (5.8) | NA |

Abbreviations: ALIF, Anterior lumbar interbody fusion; ICBG, Iliac crest bone graft; ITT, Intention to treat; NA, Not applicable; NR, Not reported; PP, Per protocol; RCT, Randomised controlled trial

Notes: Blue text indicates YHEC calculated values

1. Complications included pseudo-obstruction, pleural effusion, pneumonia, and retrograde ejaculation

**Table H14. Long term complications; other interventions**

| **Study ID** | **Study design** | **Complication description** | **Timepoint of assessment** | **Intervention** | **Number (%) of long term complications** | **p-value for difference between study arms** |
| --- | --- | --- | --- | --- | --- | --- |
| Choi 2005 [36] | Retrospective case series | Complications^1^ | 35 months (SD 8.9, range 30–42) | Allograft bone | 1/22 (4.5) | NA |
| Hironaka 2013 [38] | Retrospective case series | Complication^2^ | Mean: 76 months (range 26–146.6) | Autogenous vertebral spur and apacerum powder | 4/142 (2.8) | NA |
| Lee 2017 [39] | Retrospective case series | Postoperative complications^3^ | Mean 23.81 (17.37) | Allograft bone | 4/26 (15.4) | NA |
| Pinson 2017 [40] | Retrospective case series | Likely retrograde ejaculation | 3 years | Allograft bone | 6/64 (9.5) | NA |
| Xu 2023 [43] | Retrospective case series | Complications^4^ | 2 years | Allograft bone | 0/34 (0) | NA |

Abbreviations: ALIF, anterior lumbar interbody fusion; ICBG, iliac crest bone graft; ITT, intention to treat; NA, not applicable; NR, not reported; PP, per protocol; RCT, randomised controlled trial

Notes:

1. A patient sustained an iliolumbar vein tear during dissection, with intraoperative blood loss of approximately 600 mL, which required an immediate intraoperative ligation with hemoclip.

2. Included wound infection, liquorrhea due to a dural tear injury, vertebral body fractures needing a posterior pedicle screw fixation, and a misplaced cage that required revision.

3. 1 ASD required operation; 1 persistent radiating pain; 1 recurrent LBP; 1 iliac vein injury

4. Complications such as surgical site infection, neurovascular injury, transmission of infectious diseases and rejection reaction to the allograft
